# Identification of new triazoloquinoxaline amine derivatives with potent modulatory effects against Toll-like receptor 7 through pharmacophore-based virtual screening and molecular docking approaches

**DOI:** 10.1371/journal.pone.0336701

**Published:** 2025-12-29

**Authors:** Nasrin Saberi Harooni, Mohammad Reza Naimi-Jamal, Sajjad Gharaghani, Asal Katebi, Soheila Ajdary, Azar Tahghighi

**Affiliations:** 1 Medicinal Chemistry Laboratory, Clinical Research Department, Pasteur Institute of Iran, Tehran, Iran; 2 Department of Chemistry, Iran University of Science and Technology, Tehran, Iran; 3 Laboratory of Bioinformatics and Drug Design (LBD), Institute of Biochemistry and Biophysics, University of Tehran, Tehran, Iran; 4 Department of Immunology, Pasteur Institute of Iran, Tehran, Iran; University of Sahiwal, PAKISTAN

## Abstract

Toll-like receptor 7 (TLR7) plays a key role in the signaling pathways involved in immunity by recognizing pathogen-associated molecular patterns. Due to its broad effectiveness in various diseases and the limited number of drugs on the market that act on this receptor, it remains an ideal target for pharmaceutical scientists. To find novel TLR7 ligands, chemical feature-based pharmacophore models were prepared for TLR7 using Pharmit. This model was used for virtual screening in related databases, and the identified compounds were ranked using the Autodock docking method and minimum binding affinity. Among the identified compounds, the triazoloquinoxaline amine scaffold was selected as the lead structure, and its different derivatives were designed and docked again. Finally, two new series of [1,2,4]triazolo[4,3-a]quinoxalin-4-amine and 1-methyl-[1,2,4]triazolo[4,3-a]quinoxalin-4-amine derivatives were selected and synthesized using green chemistry routes. The cytotoxicity of the synthetic compounds was assessed using a macrophage cell line, and non-toxic compounds were then selected for evaluating cytokine stimulation. Ultimately, compound **7f** emerged as the most promising candidate for boosting the immune system based on fold change assessment compared to the control group. It exhibited the highest production of interleukin-1 (IL-1), tumor necrosis factor (TNF-α), and interferon beta (IFN-β) cytokines. Interestingly, the results of molecular docking aligned with the biological findings.

## 1 Introduction

Toll-like receptors (TLRs) are a crucial component of the innate immune system, known as pattern recognition receptors (PRRs). They recognize pathogen-associated molecular patterns (PAMPs) and damage-associated molecular patterns (DAMPs). TLRs serve as the first line of defense, protecting tissues from pathogens such as bacteria, fungi, protozoan parasites, and viruses. Additionally, by recognizing damaged or dying cells, they initiate inflammatory responses necessary for tissue repair [1–4]. These membrane glycoproteins, comprising thirteen types, are expressed on both the cell surface and intracellular organelles such as endosomes and lysosomes. Cell-surface TLRs (TLR1, TLR4–6, and TLR10) mainly bind to lipoproteins, proteins, and lipids, while some intracellular TLRs (TLR3, TLR7–9, and TLR13) detect nucleic acids, and others (TLR11 and TLR12) recognize microbial components derived from endolysosomal degradation [5]. Most TLRs signal through a specific cytoplasmic adaptor protein called Myeloid Differentiation Factor 88 (MyD88), which is ubiquitously expressed and plays a key role in intracellular signaling [6]. TLR signaling pathways can be either MyD88-dependent or MyD88-independent. The MyD88-dependent pathway leads to the production of various inflammatory cytokines, while the MyD88-independent pathway activates interferon regulatory factor (IRF), resulting in the synthesis of type 1 interferons (IFNs) [7].

Based on this, a limited number of TLRs can recognize a wide range of disease-causing pathogens and DMAPs through pattern recognition mechanisms [8]. TLRs are expressed in various cell types, including non-immune cells, such as endothelial cells, and innate immune cells such as macrophages, eosinophils, mast cells, neutrophils, basophils, natural killer (NK) cells, innate lymphoid cells, dendritic cells, and platelets. They are also present in adaptive immunity cells, including T and B cells, as well as in innate immune cells of the central nervous system, like microglia and astrocytes. Therefore, TLRs play a crucial role in both the innate and acquired immune systems and are essential for host defense [9]. Modulating TLR signaling pathways through the use of agonists, antagonists, or inhibitors while maintaining the integrity of innate immunity poses a significant challenge in the treatment of many diseases [10]. Due to their critical role in initiating and shaping adaptive immune responses against diverse antigens, TLRs have also emerged as key targets for the development of novel vaccine adjuvant aimed at enhancing immunity against a broad spectrum of pathogens [11,12].

**Fig 1:**
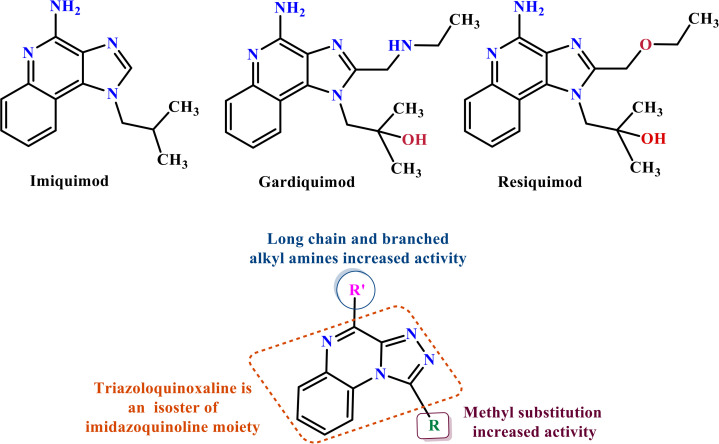
Imiquimod, gardiquimod, and resiquimod with an imidazoquinoline core, compared with the designed compounds containing a triazoloquinoxaline core.

Among them, TLR7, an endosomal pattern recognition receptor, is a key member expressed primarily in hematopoietic cells such as plasmacytoid dendritic cells (pDCs) and B cells [13]. This recognizes exogenous single-stranded RNA (ssRNA), thereby activating downstream innate immune signaling pathways [14,15], which makes it a promising therapeutic target for multiple diseases [13,16,17]. Nucleic acid-like structures, in addition to ssRNA, serve as well-known ligands for TLR7. These include small interfering RNA (siRNA) and nucleoside analogues such as imiquimod (IMQ), resiquimod (R848) and gardiquimod (GAR), all containing an imidazoquinoline structure (Fig 1) [18]. The primary molecular mechanism of TLR7 involves the MyD88-dependent pathway. Upon ligand binding in PDCs, TLR7 activates MyD88, which recruits IL-1 receptor-associated kinase (IRAK). IRAK subsequently activates TNF receptor-associated factor 6 (TRAF6), which then triggers mitogen-activated protein kinases (MAPKs) and nuclear factor kappa B (NF-κB). Furthermore, IRAK-1 regulates IFN-α production via the IRF7 pathway. Concurrently, TIR domain-containing protein TRIF-related adaptor molecule (TRAM) may modulate TLR7-induced RANTES and IFN-β production [19]. These signaling cascades culminate in the production of multiple inflammatory cytokines, such as IL-1β, IL-6, IL-12, TNF-α, chemokines and type-I interferons (IFN-α/β), all of which contribute to the amplification of inflammatory responses [20,21]. Consequently, modulation of the immune response through TLR7 agonists or antagonists hold significant therapeutic potential. TLR7 agonists activate innate immune cells, thereby promoting both humoral and cellular immunity and inducing antiviral and antitumor effects. Conversely, TLR7 antagonists may be beneficial in the management of autoimmune disorders, and other TLR7-associated clinical disorders [22].

Imidazoquinoline-based TLR7 agonists, such as IMQ, GAR, and R848, are a limited but important class of immunomodulatory drugs available on the pharmaceutical market. They exhibit potential therapeutic utility for superficial basal cell carcinoma, actinic keratosis, and genital/anogenital warts. Recently, these agents have attracted attention for their antiviral and antitumor immunomodulatory properties [23–28], as well as their potential use as vaccine adjuvants for various types of cancer and infectious diseases [29,30]. Unfortunately, the broad expression profiles of TLR7/8, the poor pharmacokinetic properties of imidazoquinoline-based TLR7s, and the toxicities associated with systemic administration remain major barriers that limit their clinical applications [29]. Given the limited number and shortcomings of current drugs, the discovery of new, efficient, safe, and cost-effective alternative TLR7 modulators has attracted significant scientific interest.

Today, in silico design, commonly utilized in the initial phases of drug discovery, has the potential to significantly expedite the transformation of research concepts into tangible results while effectively reducing the costs and resources required [31]. This in silico strategy is a valuable gift from mathematicians and computer scientists to pharmacists and biologists, enabling the study of biological processes and the prediction of the therapeutic potential of new bioactive compounds through computer simulations using theoretical computational tools [31].

Medicinal chemists have creatively employed in silico tools in combination with chemical techniques and methodologies in the field of drug discovery. To date, several groups of small-molecule modulators of TLR7, including imidazoquinolines [32,33], nucleotide analogs [34], pyrazolopyrimidines [35], quinazolinediamines [36], dihydropteridinone [37], chromenoimidazolone [38], and (triﬂuoromethyl)quinazolin-4-amine [38], have been developed using in silico studies for the management and treatment of TLR7-associated disorders.

On the other hand, green chemistry is another gift that chemists have introduced to the field of pharmaceutical science. It is a science-based philosophy aimed at replacing conventional drug synthesis, processing, and manufacturing techniques with cost-effective, sustainable, environmentally friendly, and economically profitable alternative methods [39]. Today, green chemistry is widely applied as a practical and beneficial methodology in the manufacturing of pharmaceuticals and fine chemicals. Subsequently, medicinal chemists synthesized the various bioactive compounds identified through in silico studies by applying green chemistry principles [40,41].

In this study, we leveraged the benefits of in silico studies and green chemistry techniques to design and synthesize of new compounds targeting TLR7. Two new structures of N-alkyl/aryl-[1,2,4]triazolo[4,3-a]quinoxalin-4-amine and N-(alkyl/aryl)-1-methyl-[1,2,4]triazolo[4,3-a]quinoxalin-4-amine were selected via pharmacophore-based virtual screening and molecular docking studies (Fig 1). They were then synthesized by cyclization of 2-chloro-3-hydrazinoquinoxaline with triethyl orthoformate/orthoacetate, followed by nucleophilic substitution of alkyl amines or phenyl hydrazide on the triazole ring of the tricyclic scaffold. The cytotoxicity of triazoloquinoxaline amine derivatives was evaluated on a mouse macrophage cell line. Non-toxic compounds with the highest binding energy in docking studies were selected for the evaluation of cytokines stimulation.

## 2 Experimental

### 2.1 Materials and methods

All reagents were purchased from Merck and Sigma-Aldrich Company and used without further purification. Solvents were obtained from Merck (Darmstadt, Germany). Melting points of synthetic compounds were determined using a Barnstead Electrothermal 9300 apparatus. Their proton and carbon nuclear magnetic resonance (^1^H- and ^13^C-NMR) spectra were obtained in DMSO-d_6_ on a Bruker DRX-400 and Bruker FT-500 spectrometers, using tetramethylsilane as an internal reference. The mass spectra were recorded using an Agilent Technology (HP) mass spectrometer operating at an ionization potential of 70 eV. High-resolution mass spectra (HRMS-ESI) were run on a Waters LCT Premier XE^TM^ TOF (Time of Flight) mass spectrometer. FTIR spectra were recorded as KBr pellets on a Nicolet Fourier Transform Infrared spectrophotometer. Elemental analyses were performed using the CHN rapid elemental analyzer (Thermo Finnigan, FlashEA 1112 series, Costech, Italy) to determine C, H, and N. The acceptable results were within ±0.4% of the theoretical values. Thin layer chromatography (TLC), pre-coated Merck silica gel 60 F_254_ plates, was used to monitor the progress of the reactions. Imidazoquinoline compound (R848), a TLR7/8 ligand, was purchased from InvivoGen (San Diego, CA, USA). Cell culture reagents were purchased as follows: RPMI-1640 medium from Biowest (France), penicillin and streptomycin from Gibco (Invitrogen, Paisley, UK), and L-glutamine and fetal bovine serum (FBS) from Sigma (Vienna, Austria) and Gibco (NY, USA), respectively. RNX- Plus Solution for RNA isolation was purchased from Sinaclon (Tehran, Iran). cDNA synthesis was carried out using the Easy™ cDNA synthesis kit (Parstous Biotechnology, Mashhad, Iran).

### 2.2 Pharmacophore modeling, virtual screening and molecular docking

Pharmacophore modeling was conducted using the Pharmit server (http://pharmit.csb.pitt.edu/) to generate pharmacophores and subsequently their screening against chemical compound repositories including CHEMBL (https://www.ebi.ac.uk/chembl/), and Zinc databases (https://zinc.docking.org/). CHEMBL manually curates a database of bioactive molecules with drug-like properties. The ZINC database provides 3D molecules in several formats compatible with most docking programs. The ligand-based pharmacophore model based on the modeled structure of the TLR7 complex with IMQ (PDB code: 5ZSF) and TLR7 complex with GAR (PDB code: 5ZSG) was developed using the Pharmit web server in order to identify new TLR7 ligands [42,43]. The molecular docking-based virtual screening method was performed by using AutoDock VINA implemented in the PyRx 0.8 tool. To validate the docking results, the ligand from the crystallographic structure was docked in a similar manner. The RMSD between the docked ligand and the crystallographic ligand was calculated. The RMSD value of 0.996 Å was considered acceptable.

### 2.3 Chemistry

Refer to Scheme 1.

**Scheme 1:**
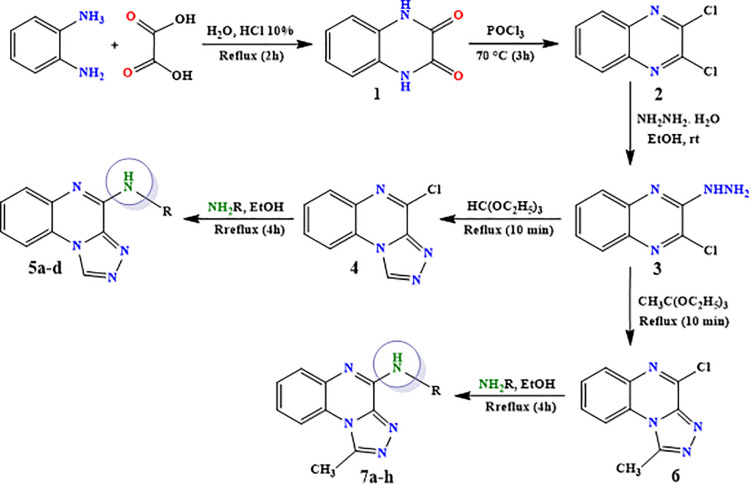
Synthesis route of intermediates and final compounds 5a-d and 7a-h.

#### 2.3.1 Procedure for the synthesis of compound 1.

A mixture of benzene-1,2-diamine (1 g), oxalic acid (1.5 g), and 3 mL of 10% HCl in 3 mL of H_2_O was stirred at 100 °C for 2 hours. The mixture was cooled and then filtered to obtain 1,4 dihydroquinoxaline-2,3-dione (**1**) as a white solid. Yield: 95%; m.p.: 300 °C (Lit. ≤ 300 °C) [44].

#### 2.3.2 Procedure for the synthesis of compound 2.

To a stirred solution of quinoxaline-2,3(1H,4H)-dione (1 g), POCl_3_ (20 mL) was added and refluxed at 100 °C for 26 hours. After completion of the reaction, the mixture was distilled under vacuum and then recrystallized from ice-cold water [45]. A white solid, 2,3-dichloroquinoxaline, was obtained in excellent yield (99%); m.p.: 148−151 °C (Lit. 148−150 °C) [46]. FTIR (KBr, cm^-1^): 3007 (CH aromatic), 1600 & 1490 (C = C aromatic), 1582 (C = N), 750 (C-Cl); MS (m/z): 202 (M^+2^), 200 (M^+1^), 198 (M^+^).

#### 2.3.3 Procedure for the synthesis of compound 3.

2,3-Dichloroquinoxaline (**2**) 0.197 g, 1 mmol) was dissolved in 5 mL of ethanol and then hydrazine hydrate (0.48 mL, 1.5 mmol) was added. The reaction mixture was stirred overnight at room temperature. The resulting precipitate was filtered, washed with ethanol, and dried in air to give a yellow solid. Yield: 90%; m.p.: 182−184 °C (Lit. 181−182 °C) [47]. FTIR (KBr, cm^-1^): 3360.06 (NH_2_), 3350.26 (NH stretching), 3007.10 (CH aromatic), 1600.04 & 1490.00 (C = C aromatic), 1582.32 (NH bending), 749.90 (C-Cl); MS (m/z): 196.1 (M^+^^1^), 194.1 (M^+^).

#### 2.3.4 Procedure for the synthesis of compound 4.

A mixture of 2-chloro-3-hydrazinoquinoxaline (**3**) (1 g, 5 mmol) and 20 mL of triethyl orthoformate was stirred under reflux for 10 minutes, then cooled to room temperature. The solid was washed with ether and dried at room temperature. This compound was obtained as a yellow solid [48]. Yield: 88%; ^1^H-NMR (500 MHz, DMSO-d6) δ ppm: 7.70 (1H, t, *J* = 8.06 Hz, Ar), 7.85 (1H, t, *J* = 8.06 Hz, Ar), 8.05 (1H, d, *J* = 8.06 Hz, Ar) 8.45 (1H, d, *J* = 8.06 Hz, Ar), 10.03 (1H, s, CH triazole).

#### 2.3.5 Procedure for the synthesis of compound 6.

A mixture of 2-chloro-3-hydrazinoquinoxaline (1 g, 5 mmol) and 20 mL of triethyl orthoacetate was stirred at reflux for 10 minutes, then cooled to room temperature. The solid washed with ether and dried at room temperature. This compound was obtained as a yellow solid. Yield: 93%; m.p.: 157–161°C (Lit. 158–160 °C) [48].

#### 2.3.6 Procedure for the synthesis of compound 5a-d and 7a-h.

A mixture of 4-chloro[1,2,4]triazolo[4,3-a]quinoxaline (**4**) or 4-chloro-1-methyl-[1,2,4]triazolo[4,3-a]quinoxaline (**6**) (2 mmol) and different aliphatic amines (2.3 mmol) was refluxed in ethanol (15 mL) for 4 ~ 5 hours. Then the solvent was then evaporated under reduced pressure. The resulting precipitate was filtered, dried, and crystallized from absolute ethanol to obtain the corresponding target compounds **5a-d** or **7a-h**. All spectra of the final compounds (^1^H-NMR, ^13^C-NMR, FTIR, CHN analysis, and HRMS-ESI) are shown in S3 Fig.

**2.3.6.1 Compound 5a: N-Isopropyl-[1,2,4]triazolo[4,3-a]quinoxalin-4-amine**: Yellow powder; m.p.: 130−133 °C; ^1^H-NMR (400 MHz, DMSO- d6) δ ppm: 1.35 (3H, d, *J* = 7.6 Hz, -CH_3_), 3.25 (1H, m, *J* = 7.6 Hz, -CH_2_-), 7.25 (1H, t, *J* = 8.1 Hz, Ar), 7.35 (1H, t, *J* = 8.1 Hz, Ar), 8.00 (1H, d, *J* = 8.1 Hz, Ar), 8.20 (1H, d, *J* = 8.1 Hz, Ar), 7.99 (1H, s, -NH-), 10.00 (1H, s, CH triazole); MS (m/z): 227.2 (M^+^); HRMS-ESI(+) (m/z): calc. for C_12_H_13_N_5_ [M + H]^+^ 228.1205, found 228.1243; FTIR (KBr, cm^-1^): 3432.20 (NH), 3007.05 (CH aromatic), 3194.3 (CH aliphatic), 1652.90 & 1511.05 (C = C aromatic), 1582.45 (C = N triazole).

**2.3.6.2 Compound 5b: N-Butyl-[1,2,4]triazolo[4,3-a]quinoxalin-4-amine**: Faint yellow; m.p.: 150−153 °C; ^1^H-NMR (500 MHz, DMSO-d_6_) δ ppm: 0.99 (3H, t, *J* = 5.8 Hz, -CH_3_), 1.40 (2H, m, *J* = 5.8 Hz, -CH_2_-), 1.60 (2H, m, *J* = 5.8 Hz, -CH_2_-), 3.6 (2H, m, *J* = 5.8 Hz, -CH_2_-), 7.31 (1H, t, *J* = 7.4 Hz, Ar), 7.51 (1H, t, *J* = 7.4 Hz, Ar), 7.60 (1H, d, *J* = 7.4 Hz, Ar), 8.22 (1H, d, *J* = 7.4 Hz, Ar), 8.33 (1H, d, *J* = 7.4 Hz, -NH-), 10.00 (1H, s, CH triazole); ^13^C-NMR (125 MHz, DMSO-d6) δ ppm: 13.36, 19.06, 28.90, 38.35, 116.24, 116.77, 120.36, 127.90, 128.09, 128.88, 130.22, 138.44, 145.11; MS (m/z): 241.1 (M^+^); HRMS-ESI(+) (m/z): calc. for C_13_H_15_N_5_ [M + H]^+^ 242.1361, found 242.1406; FTIR (KBr, cm^-1^): 3393.41 (NH), 3089.28 (CH aromatic), 2937.32 (CH aliphatic), 1634.02 & 1482.08 (C = C aromatic), 1558.10 (C = N triazole).

**2.3.6.3 Compound 5c: N-(4-Methylpentan-2-yl)-[1,2,4]triazolo[4,3-a]quinoxalin-4-amine:** Yellow powder; m.p.: 155−157 °C; ^1^H-NMR (400 MHz, DMSO-d6) δ ppm: 0.99 (3H, d, *J* = 6.5 Hz, -CH_3_), 1.25 (3H, d, *J* = 6.5 Hz, -CH_3_), 1.35 (2H, m, *J* = 6.5 Hz, -CH_2_-), 1.65 (1H, m, *J* = 6.5 Hz, -CH-), 1.75 (1H, m, *J* = 6.5 Hz, -CH-), 7.30 (1H, t, *J* = 8.1 Hz, Ar), 7.45 (1H, t, *J* = 8.1 Hz, Ar), 7.58 (1H, d, *J* = 8.1 Hz, Ar), 8.02 (1H, d, *J* = 8.1 Hz, -NH-), 8.15 (1H, d, *J* = 8.1 Hz, Ar), 10.00 (1H, s, CH triazole); ^13^C-NMR (100 MHz, DMSO-d6) δ ppm: 18.27, 22.61, 23.74, 43.28, 44.97, 115.99, 121.60, 122.77, 125.81, 127.34, 137.18, 137.87, 138.16, 145.10; MS (m/z): 269.16 (M ^+^); FTIR (KBr, cm^-1^): 3412.02 (NH), 3119.15 (CH aromatic), 3266.05 (CH aliphatic), 1679.31 & 1532.36 (C = C aromatic), 1605.25 (C = N triazole). Anal. Calcd for C_15_H_19_N_5_: C, 66.89; H, 7.11; N, 26.00. Found: C, 66.39; H, 7.50; N, 26.11.

**2.3.6.4 Compound 5d: 4-(2-Phenylhydrazineyl)-[1,2,4]triazolo[4,3-a]quinoxaline:** Soft yellow; m.p.: 285−291 °C; ^1^H-NMR (400 MHz, DMSO-d6) δ ppm: 6.70 (1H,t, *J* = 8.0 Hz, Ar), 6.75 (1H, t, *J* = 8.0 Hz, Ar), 6.85 (1H, d, *J* = 8.0 Hz, Ar), 7.15 (5H, m, Ar), 7.55 (1H, d, *J* = 8.0 Hz, Ar), 8.00 (1H, d, -NH-), 9.60 (1H, d, -NH-), 9.95 (1H, s, CH triazole); MS (m/z): 276.2 (M^+^); FTIR (KBr, cm^-1^): 3373.1 (NH), 3059.7 (CH aromatic), 1511.1 (C-N hydrazine), 1667.8 & 1589.4 (C = C aromatic), 1354.3 (C = N triazole).

**2.3.6.5 Compound 7a: N-Ethyl-1-methyl-[1,2,4]triazolo[4,3-a]quinoxalin-4-amine:** Yellow powder; m.p.: 130−134 °C; ^1^H-NMR (500 MHz, DMSO-d6) δ ppm: 1.22 (3H, t, *J* = 7.2 Hz, -CH_3_), 3.00 (3H, s, -CH_3_ triazole), 3.50 (2H, m, *J* = 7.2 Hz, -CH_2_-), 7.20 (1H, t, *J* = 8.0 Hz, Ar), 7.52 (1H, t, *J* = 8.0 Hz, Ar), 7.60 (1H, d, *J* = 8.0 Hz, Ar), 8.20 (1H, d, *J* = 8.0 Hz, Ar), 8.20 (1H, d, *J* = 8.0 Hz, -NH-); ^13^C-NMR (125 MHz, DMSO-d6) δ ppm: 12.96, 14.52, 34.48, 124.79, 125.86, 127.88, 128.36, 130.63, 135.76, 138.34, 141.49, 148.59; MS (m/z): 227.2 (M^+^); FTIR (KBr, cm^-1^): 3529.02 (NH), 3059.30 (CH aromatic), 2903.52 (CH aliphatic), 1667.03 & 1511.05 (C = C aromatic), 1589.12 (C = N triazole).

**2.3.6.6 Compound 7b: 1-Methyl-N-propyl-[1,2,4]triazolo[4,3-a]quinoxalin-4-amine:** Yellow powder; m.p.: 154−155 °C; ^1^H-NMR (400 MHz, DMSO-d6) δ ppm: 0.99 (3H, t, *J* = 7.0 Hz, -CH_3_), 1.50 (2H, m, *J* = 7.0 Hz, -CH_2_-), 3.51 (2H, m, *J* = 7.0 Hz, -CH_2_-), 3.51 (1H, s, -CH_3_ triazole), 7.32 (1H, t, *J* = 8.1 Hz, Ar), 7.45 (1H, t, *J* = 8.1 Hz, Ar), 7.61 (1H, d, *J* = 8.1 Hz, Ar), 8.11 (1H, d, *J* = 7.0 Hz, -NH-), 8.15 (1H, d, *J* = 8.1 Hz, Ar); ^13^C-NMR (100 MHz, DMSO-d6) δ ppm: 11.36, 11.96, 20.86, 22.36,116.22, 123.27, 123.90, 126.63, 127.46, 138.14, 140.03, 146.33, 148.78; MS (m/z): 241.3 (M^+^); HRMS-ESI(+) (m/z): calc. for C_13_H_15_N_5_ [M + H]^+^ 242.1361, found 242.1400; FTIR (KBr, cm^-1^): 3544.20 (NH), 3393.32 (CH aromatic), 2937.05 (CH aliphatic), 1634.21 & 1482.30 (C = C aromatic), 1558.08 (C = N triazole).

**2.3.6.7 Compound 7c: N-Isopropyl-1-methyl-[1,2,4]triazolo[4,3-a]quinoxalin-4-amine:** White powder; m.p.: 184−186 °C; ^1^H-NMR (400 MHz, DMSO-d6) δ ppm: 1.29 (3H, d, *J* = 8 Hz, -CH_3_), 3.03 (3H, s, -CH_3_ triazole), 4.54 (1H, m, *J* = 8 Hz, -CH-), 7.30 (1H, t, *J* = 4 Hz, Ar), 7.45 (1H, t, *J* = 4 Hz, Ar), 7.60 (1H, d, *J* = 4 Hz, Ar), 7.92 (1H, d, *J* = 4 Hz, -NH-), 8.10 (1H, d, *J* = 4 Hz, Ar); ^13^C-NMR (100 MHz, DMSO-d6) δ ppm: 14.38, 20.28, 42.84, 115.61, 122.74, 126.12, 126.88, 131.67, 137.63, 139.48, 145.05,148.18; MS (m/z): 241.2 (M^+^); FTIR (KBr, cm^-1^): 3544 (NH), 3241.03 (CH aromatic), 2937.05 (CH aliphatic), 1634.30 & 1482.12 (C = C aromatic), 1558.29 (C = N triazole); Anal. Calcd for C_13_H_15_N_5_: C, 64.71; H, 6.27; N, 29.02. Found: C, 64.51; H, 6.47; N, 29.02.

**2.3.6.8 Compound 7d: N-(sec-Butyl)-1-methyl-[1,2,4]triazolo[4,3-a]quinoxalin-4-amine:** Yellow powder; m.p.: 152−156 °C; ^1^H-NMR (400 MHz, DMSO-d6) δ ppm: 0.90 (3H, d, *J* = 7.2 Hz, -CH_3_), 1.25 (2H, d, *J* = 7.2 Hz, -CH_2_-), 1.61 (2H, m, *J* = 7.2 Hz, -CH_2_-), 1.70 (1H, m, *J* = 7.2 Hz, -CH-), 3.03 (3H, s, -CH_3_ triazole), 7.3 (1H, t, *J* = 8.3 Hz, Ar), 7.45 (1H, t, *J* = 8.3 Hz, Ar), 7.63 (1H, d, *J* = 8.3 Hz, Ar), 7.85 (1H, d, *J* = 8.3 Hz, -NH-), 8.14 (1H, d, *J* = 8.3 Hz, Ar); ^13^C-NMR (100 MHz, DMSO-d6) δ ppm: 14.30, 17.52, 46.86, 47.99, 115.62, 122.68, 123.34, 126.11, 126.88, 137.66, 139.48,145.46,148.23; MS (m/z): 255.1 (M^+^); FTIR (KBr, cm^-1^): 3544.35 (NH), 3089.52 (CH aromatic), 2937.54 (CH aliphatic), 1634.23 & 1558.16 (C = C aromatic), 1634.25 (C = N triazole); Anal. Calcd for C_14_H_17_N_5_: C, 65.86; H, 6.71; N, 27.43. Found: C, 65.86; H, 6.51; N, 27.63.

**2.3.6.9 Compound 7e: N-Isobutyl-1-methyl-[1,2,4]triazolo[4,3-a]quinoxalin-4-amine:** Yellow powder; m.p.: 130−134 °C; ^1^H-NMR (400 MHz, DMSO-d6) δ ppm: 0.99 (3H, d, *J* = 6.7 Hz, -CH_3_ & -CH_3_), 2.00 (1H, m, *J* = 6.7 Hz, -CH-), 3.00 (3H, s, -CH_3_ triazole), 3.45 (2H, m, *J* = 6.7 Hz, -CH_2_-), 7.30 (1H, t, *J* = 8.3 Hz, Ar), 7.45 (1H, t, *J* = 8.3 Hz, Ar), 7.61 (1H, d, *J* = 8.3 Hz, Ar), 8.10 (1H, d, *J* = 8.3 Hz, Ar), 8.20 (1H, d, *J* = 8.3 Hz, -NH-); MS (m/z): 255.3 (M^+^); FTIR (KBr, cm^-1^): 3412.65 (NH), 3266.50 (CH aromatic), 2972.23 (CH aliphatic), 1605.23 & 1458.36 (C = C aromatic), 1532.30 (C = N triazole); Anal. Calcd for C_14_H_17_N_5_: C, 65.86; H, 6.71; N, 27.43. Found: C, 64.68; H, 7.71; N, 27.61.

**2.3.6.10 Compound 7f: 1-Methyl-N-(4-methylpentan-2-yl)-[1,2,4]triazolo[4,3-a]quinoxalin-4-amine:** Yellow powder; m.p.: 225−228 °C; ^1^H-NMR (400 MHz, DMSO-d6) δ ppm: 0.99 (6H, t, *J* = 6.5 Hz, -CH_3_), 1.33 (2H, m, *J *= 6.5 Hz, -CH_2_-), 1.46 (1H, m, *J *= 6.5 Hz, -CH-), 1.70 (2H, m, *J* = 6.5 Hz, -CH_3_), 3.01 (1H, s, -CH_3_ triazole), 3.20 (1H, m, *J* = 8 Hz, -CH-), 7.21 (1H, t, *J* = 8.3 Hz, Ar), 7.40 (1H, t, *J* = 8.3 Hz, Ar), 7.61 (1H, d, *J* = 8.3 Hz, Ar), 7.92 (1H, d, *J* = 8.3 Hz, Ar), 8.10 (1H, d, *J* = 6.5 Hz, -NH-); ^13^C-NMR (100 MHz, DMSO-d6) δ ppm: 21.68, 22.23, 22.74, 23.74, 43.23, 44.98, 115.53, 122.56, 123.27, 126.05, 126.82, 137.69, 139.44, 145.34,148.17; MS (m/z): 283.3 (M^+^); FTIR (KBr, cm^-1^): 3432.82 (NH), 3291.44 (CH aromatic), 2865.11 (CH aliphatic), 1652.25 & 1440.17 (C = C aromatic), 1582.43 (C = N triazole). Anal. Calcd for C_16_H_21_N_5_: C, 67.82; H, 7.47; N, 24.71. Found: C, 67.72; H, 7.47; N, 24.81.

**2.3.6.11 Compound 7g: N-Hexyl-1-methyl-[1,2,4]triazolo[4,3-a]quinoxalin-4-amine:** White powder; m.p.: 189−192 °C; ^1^H-NMR (400 MHz, DMSO-d6) δ ppm: 0.89 (3H, d, *J* = 6.8 Hz, -CH_3_), 1.30 (6H, m, *J* = 6.8 Hz, -CH_2_-), 1.65 (2H, m, *J* = 6.8 Hz, -CH_2_-), 3.03 (3H, s, -CH_3_ triazole), 7.30 (1H, t, *J* = 8.3 Hz, Ar), 7.45 (1H, t, *J* = 8.3 Hz, Ar), 7.60 (1H, d, *J* = 8.3 Hz, Ar), 8.10 (1H, d, *J* = 8.3 Hz, Ar), 8.16 (1H, t, *J* = 8.3 Hz, -NH-); ^13^C-NMR (100 MHz, DMSO-d6) δ ppm: 14.43, 14.96, 22.57, 26.65, 29.01, 31.51, 116.26, 123.24, 123.92, 126.65, 127.48, 138.19,140.05,146.32,148.78; MS (m/z): 283.3 (M^+^); HRMS-ESI(+) (m/z): calc. for C_16_H_21_N_5_ [M + H]^+^ 284.1831, found 284.1863; FTIR (KBr, cm^-1^): 3291.03 (NH), 3007.32 (CH aromatic), 2865.87 (CH aliphatic), 1652.90 & 1511.06 (C = C aromatic), 1582.12 (C = N triazole).

**2.3.6.12 Compound 7h: 1-Methyl-4-(2-phenylhydrazineyl)-[1,2,4]triazolo[4,3-a]quinoxaline:** Yellow powder; m.p.: 285–292 °C; ^1^H-NMR (400 MHz, DMSO-d6) δ ppm: 2.95 (3H, s, -CH_3_ triazole), 6.53 (1H, t, *J* = 8.3 Hz, Ar), 6.60 (1H, t, *J* = 8.3 Hz, Ar), 6.75 (1H, d, *J* = 8.3 Hz, Ar), 7.38 (5H, m, Ar), 8.16 (1H, d, *J* = 8.3 Hz, Ar), 9.53 (1H, s, -NH-), 10.01 (1H, s, -NH-); MS (m/z): 291.1 (M^+^); FTIR: 3149.3 (NH), 3007.5 (CH aromatic), 1723.8 & 1582.0 (C = C aromatic), 1369.2 (C = N triazole). Anal. Calcd for C_16_H_14_N_6_: C, 66.19; H, 4.86; N, 28.95. Found: C, 66.24; H, 4.76; N, 29.00.

### 2.4 Evaluation of cytotoxicity of triazoloquinoxaline amine derivatives

The MTT assay was applied to evaluate the cytotoxicity of triazoloquinoxaline amine derivatives on J774A.1 mouse macrophage cell line based on a previous method [49]. In brief, the cells (2 × 10^5^ cells per well) in 96-well plate incubated with various concentrations of the synthetic compounds (0.468–60 µg/ml) at 37 °C, 5% CO_2_ for 24 hours. Untreated cells were used as controls. Afterwards, 20 μl of MTT reagent (0.5 mg/mL) was added to each well and incubated for 3 hours. Subsequently, the medium was removed, and 100 μl of DMSO was added to each well. The plate was shaken for 10 minutes to dissolve the formazan crystals. The test was performed in triplicate and absorbance was measured at 570 nm using a microplate reader (BioTek ELx808 Absorbance Microplate Reader).

### 2.5 Evaluation of cytokines expression

The effects of triazoloquinoxaline amine derivatives on J774.1 were determined by qPCR as previously described [49]. Briefly, cells were cultured in 24 well plates at 37 °C and 5% CO_2_ for 24 hours. Then, the synthesized compounds were added to the wells at a concentration of 10 µg/ml and incubated at 37 °C and 5% CO_2_ for 24 hours. Afterward, the cells were washed, and total RNA was extracted using RNX-plus buffer according to the manufacturer’s guidelines. Reverse transcription was then performed using a cDNA synthesis kit Relative quantification was performed with a Rotor-Gene 6000 thermal cycler (Corbett Research, Mortlake, Victoria, Australia),) in a reaction volume of 10 μL containing 5 μL Real Q-PCR 2 × Master Mix, 10 pM from each forward and reverse primer, 1 μL cDNA, and 2 μL DEPC water (Sinaclon, Iran) to amplify IL-1, IL-6, TNFα, IFN-β, and Hprt (Hypoxanthine-guanine-phosphoribosyltransferase) as a reference gene. The sequences of primers are listed in Table 1. The thermal profile was as follows: 94 °C for 15 minutes, followed by 40 cycles of 95 °C for 15 seconds, and 62 °C for 20 seconds. Each set of reactions also included negative (no cDNA) and RT (nonspecific RNA) controls. The mRNA transcription levels of cytokine genes were determined by relative quantification using HPRT expression as the reference gene for normalization. The results were analyzed by the comparative threshold cycle methods (2^−ΔΔCt^) described by Livak and Schmittgen. Relative levels of gene transcription were quantified as the fold increase (mean + SD) in the treated cells vs. the untreated cells. All PCR reactions were performed in duplicates.

**Table 1:** Primer sequences used for qPCR.

Primers	Base sequences
IL-1β	F:5’-TGATGAGAATACCTGTT
	R: 5’-CTGCTGCGAGATTTGAAG
IL-6	F:5’-TGATGGATGCTACAAACTG
	R: 5’-TGTACTCCAGGTAGCTATGG
TNF-α	F:5’-CCTATGTCTCAGCCTCTTCT
	R: 5’-GGGAACTTCTCATCCCTTTG
HGPRT	F:5’-CTCAACTTTAACTGGAAAGAATGT
	R: 5’-GGGCTGTACTGCTTAACC

F: Forward; R: Reverse.

### 2.6 Statistical analysis

SPSS software (version 22) was applied for statistical analysis. Data were analyzed using one-way ANOVA, followed by Tukey post-hoc test. Results were reported as mean ± standard deviations (mean±SD), and p-values ≤ 0.05 were considered statistically significant.

## 3 Results

### 3.1 In silico studies

**Fig 2:**
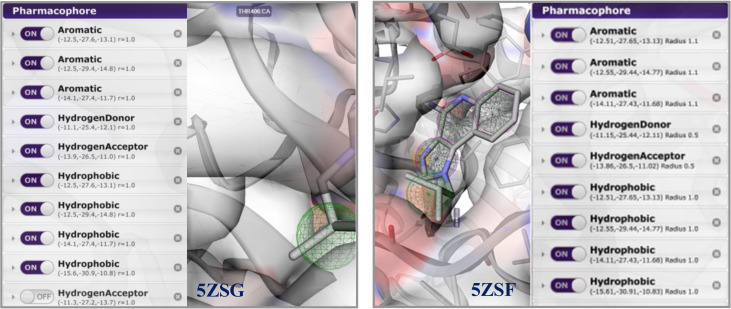
The pharmacophore model was constructed using Pharmit, based on the interactions between TLR7/GAR and TLR7/IMQ complexes (PDB code: 5ZSG and 5ZSF).

For the preparation and confirmation of new TLR7 compounds based on bioinformatics studies, the structure of the TLR7 complex with IMQ (PDB code: 5ZSF) was downloaded from the Protein Data Bank, and the pharmacophore model was built using Pharmit. The purpose of using this server was to evaluate the bioavailability and drug-likeness of lead compounds identified by filtering and prioritizing ligands based on their similarity to the pharmacophore model created on the Pharmit server. Several studies have described similar approaches for the development of pharmacophores using the Pharmit server [42,50]. The pharmacophore model included three aromatic features, one hydrogen bond donor (HBD), one hydrogen bond acceptor (HBA), and four hydrophobic interactions (Fig 2). This model was utilized for virtual screening in two databases ZINC and CHEMBL. Upon searching the ZINC database, 646 hits were obtained. By considering the exclusive shape, the number of compounds was reduced to 42. In the final stage, the results were refined using the Vina docking method and ranked according to the lowest binding affinity. Similarly, an initial search in the CHEMBL database yielded 1344 hits. After considering the exclusive shape, the number of compounds was reduced to 484. Following the application of Lipinski’s rule of five, 363 compounds remained. The final results were minimized through docking and ranked according to the best binding affinity.

The structure of the TLR7 complex with GAR (PDB code: 5ZSG) was downloaded from the PDB, and a pharmacophore model was constructed using Pharmit. This model was used for virtual screening in both the ZINC and CHEMBL databases. The process continued similarly to the route mentioned above, and finally, three compounds remained that were minimized through docking and ranked according to the lowest binding affinity. Finally, the [1,2,4]triazolo[4,3-a]quinoxaline amine scaffold was considered as the main core of the synthetic derivatives and designed with various substitutions on the triazole and quinoxaline rings (Fig 1). Before synthesis, molecular docking studies confirmed the lowest binding energy of these compounds to the active site of the Toll-like receptor 7. The results of the docking study introduced some of the most important amino acids shared in the active site of the TLR7 receptor in interaction with IMQ, R848, and the designed compounds, including Asp555, Thr586, leu557, Thr532, Tyr356, Val355, Phe408, and Ile585.

**Fig 3:**
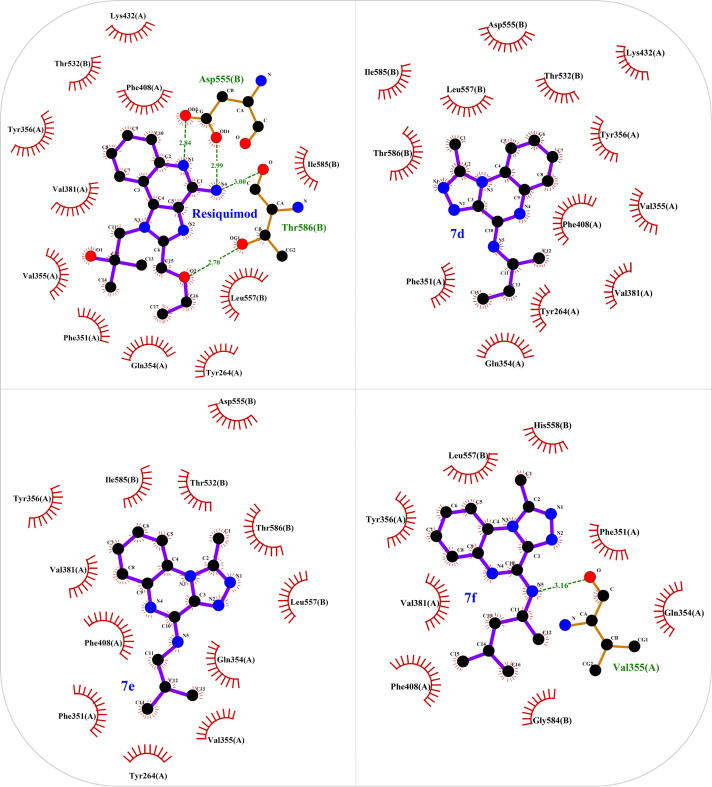
Two-dimensional interaction of selected compounds from the N-alkyl-1-methyl-[1,2,4]-triazole[3,4-a]-quinoxaline-4-amine series (7d-7f) and resiquimod with TLR7 protein based on docking studies.

As shown in Fig 3 and S1 Fig, the crystal structures of TLR7 in complex with the proposed compounds revealed important binding interactions that play a key role in receptor occupancy and biological activity. Accordingly, R848 and IMQ interact with amino acids Tyr264(A), Phe351(A), Gln354(A), Val355(A), Tyr356(A), Val381(A), Phe408(A), Lys432(A), Thr532(B), Asp555(B), Leu557(B), Ile585(B), and Thr586(B) in the receptor active site. The compounds selected based on docking studies also exhibited interactions similar to existing drugs with the active site of the receptor (Fig 3). For instance, compound **7f**, which showed low binding energy, interacted with Tyr264(A), Phe351(A), Gln354(A), Val355(A), Tyr356(A), Val381(A), Phe408(A), Lys432(A), Thr532(B), Leu557(B), Asp555(B), Ile585(B), and Thr586(B), exactly like R848 and IMQ (Fig 3 and S1 Fig).

### 3.2 Chemistry

In this study, two new series of compounds N-alkyl/aryl-[1,2,4]triazolo[4,3-a]quinoxalin-4-amine and N-(alkyl/aryl)-1-methyl-[1,2,4]triazolo[4,3-a]quinoxalin-4-amine, bearing alkyl or aryl amine substituents on the triazoloquinoxaline scaffold were synthesize in several steps. Firstly, 1,4-dihydroquinoxaline-2,3-dione was synthesized as the initial intermediate (**1**) by reacting benzene-1,2-diamine with oxalic acid. Subsequently, 2,3-dichloroquinoxaline (**2**) was prepared by chlorination of 1,4-dihydroquinoxaline-2,3-dione (**1**) in the presence of excess POCl_3_. Intermediate **2** then reacted with hydrazine hydrate to produce 2-chloro-3-hydrazinoquinoxaline (**3**), which, in the presence of triethyl orthoformate/ortho acetate, yielded the final intermediates **4** and **6**, respectively [51]. These intermediates subsequently underwent nucleophilic substitution reactions with alkyl amines or phenylhydrazine to produce the final compounds N-alkyl/aryl-[1,2,4]triazolo[4,3-a]quinoxalin-4-amine (**5a-d**) and N-(alkyl/aryl)-1-methyl-[1,2,4]triazolo[4,3-a]quinoxalin-4-amine (**7a-h**). The conditions of all reactions were optimized in terms of green solvent, temperature, and time to achieve the highest yield without the need for complex purification (Table 2). A variety of short-chain, long-chain, and branched aliphatic amines, as well as phenyl hydrazine, effectively reacted with chloroquinoxaline under green conditions. Laboratory results showed that most primary aliphatic amines bearing electron-donating groups reacted efficiently with quinoxaline, giving yields of ≥ 80% (Table 2). However, the nucleophilic attack of phenylhydrazine on chloroquinoxalines resulted in lower yields (≤ 70%) (Table 2). The structures of the synthesized compounds were confirmed by FTIR, nuclear magnetic resonance spectroscopy (H-NMR and C-NMR), mass spectrometry, HRMS-ESI, and CHN analysis.

**Table 2:** Chemical structure of compounds selected based on molecular docking studies, binding affinities, reaction times, and yields of the synthesized compounds.

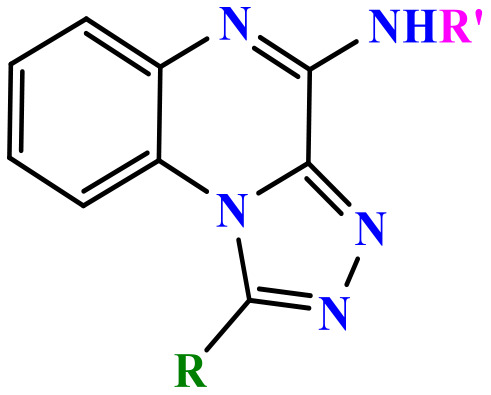					
Entry	R/R'	Product	Binding affinity* (Kcal/mol)	Time (h)	Yield (%)
5a	H/Iso-propylamine	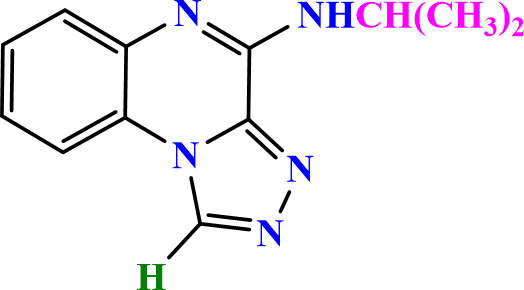	-7.8	4.25	80
5b	H/Buthylamine	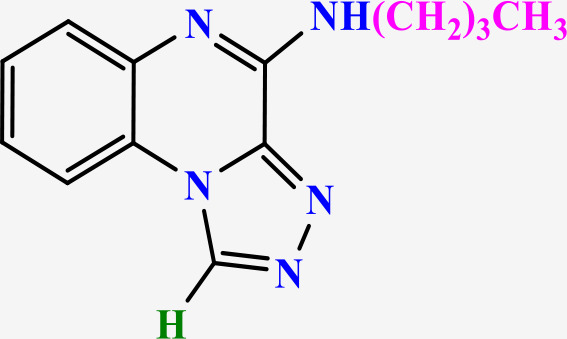	-7.4	4	85
5c	H/4-Methyl pentyl	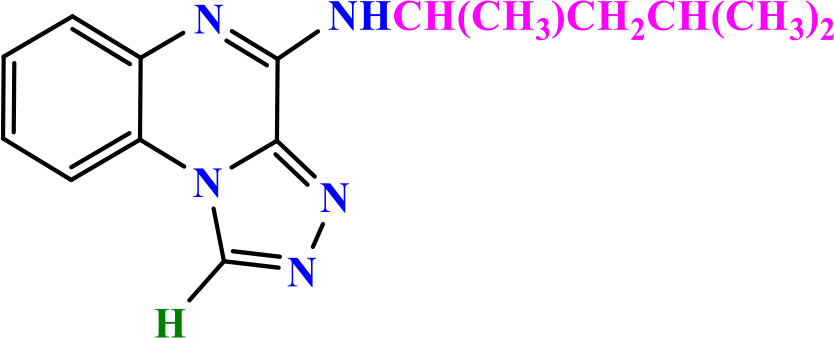	-8	4.15	80
5d	H/ phenylhydrazineyl	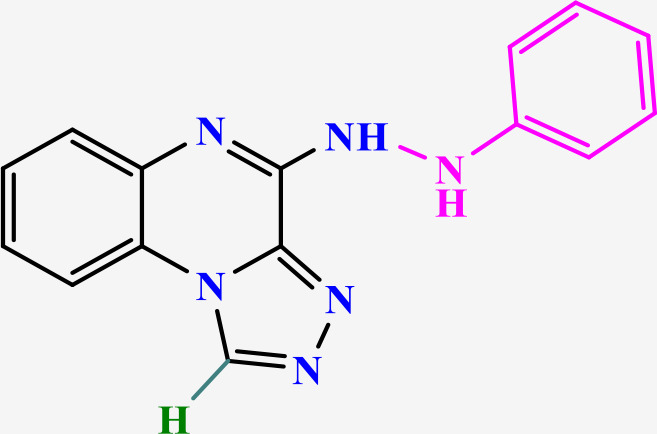	-8.8	5	60
7a	Me/Ethyl	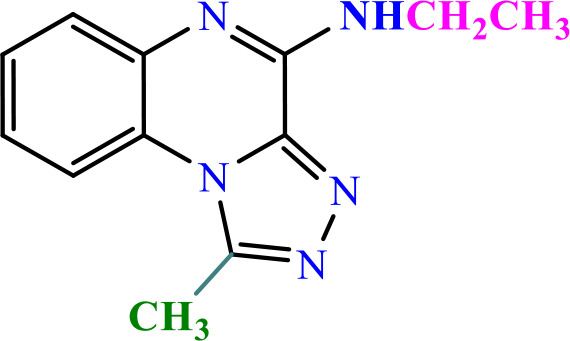	-7.9	4.15	80
7b	Me/Propyl	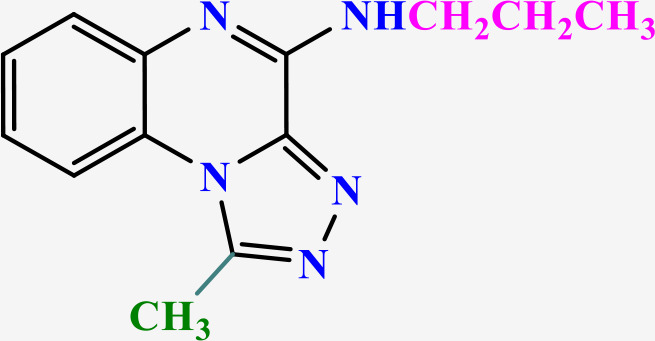	-7.9	4.15	75
7c	Me/Isopropyl	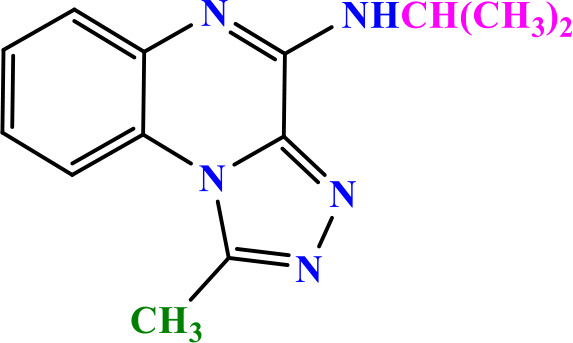	-8.3	4.15	80
7d	Me/sec-Butyl	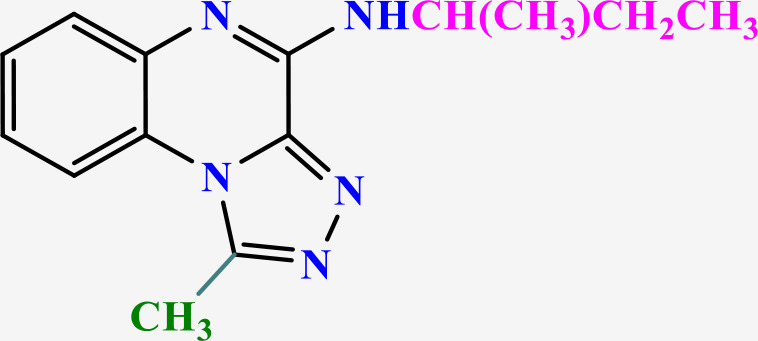	-8.2	4.15	73
7e	Me/Iso-Butyl	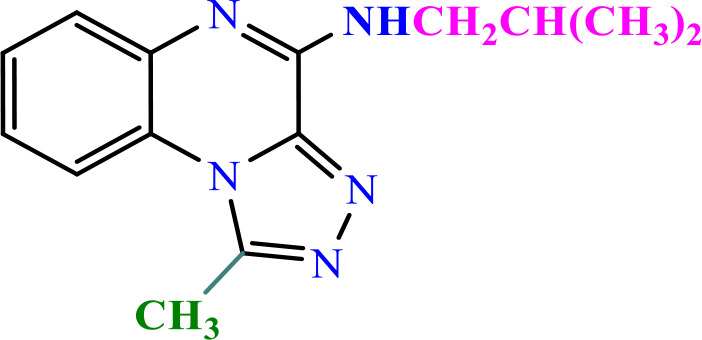	-8	4	80
7f	Me/4-Methyl pentyl	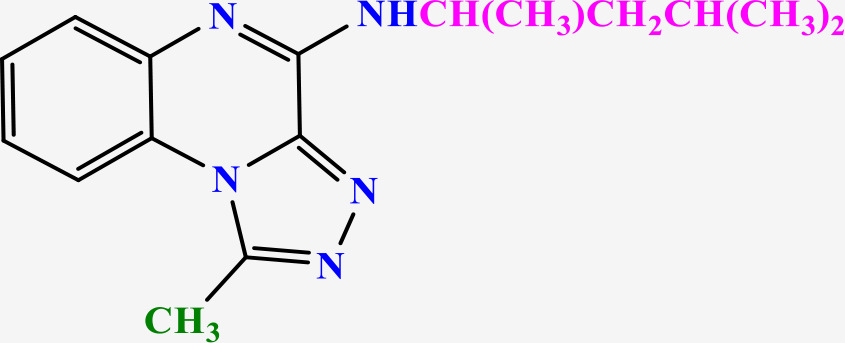	-8.1	4	85
7g	Me/Hexyl	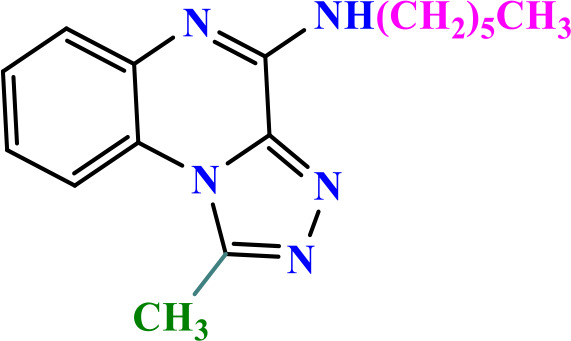	-7.8	4.15	85
7h	Me/ phenylhydrazineyl	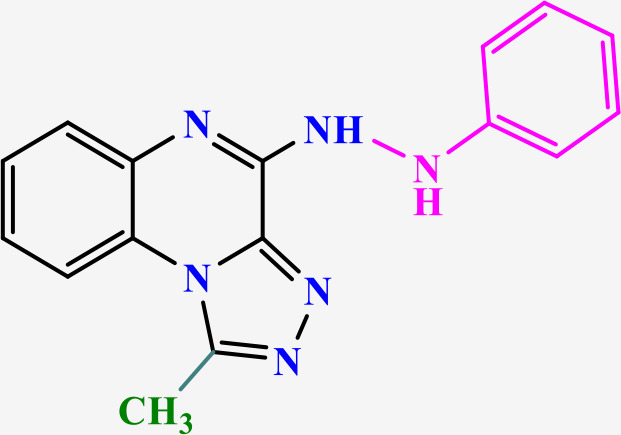	-9.1	4.30	70

*Binding affinity of resiquimod was equal −7.1 Kcal/mol.

### 3.3 The cytotoxicity of synthetic compounds

**Fig 4:**
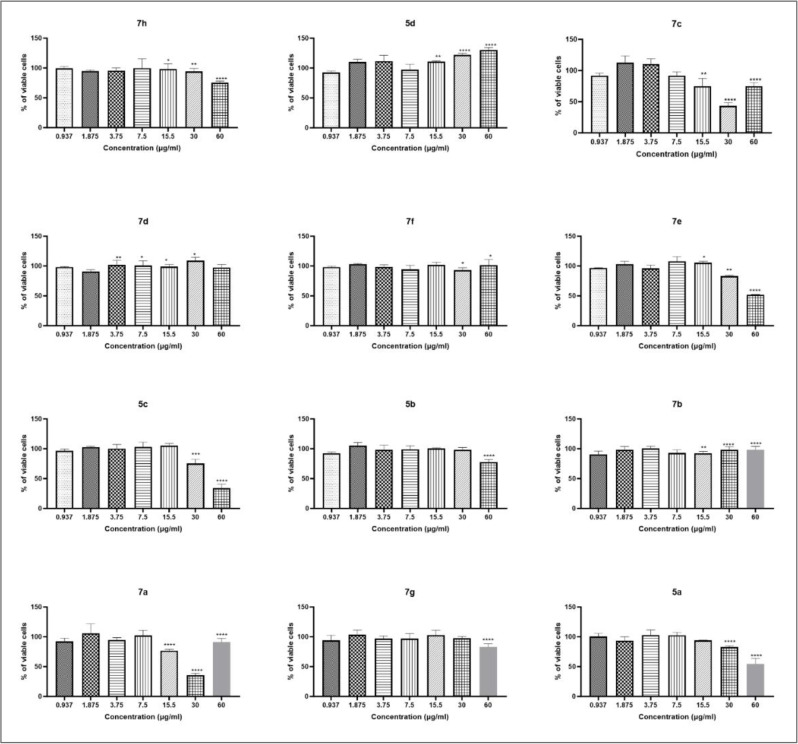
Evaluation of cytotoxicity of triazolo quinoxalineamine derivatives (5a-d & 7a-h). Cells were treated with different concentrations of the compounds for 24 h, and cell viability was determined by the MTT assay. Values represent mean+ SD (n = 3). ****, ***, ** and * represent *p <* 0.0001, 0.001, 0.01 and 0.05, respectively.

An MTT assay was employed to assess the cytotoxicity of the synthesized derivatives, which were selected based on docking studies, on the J774.1 macrophage cell line. Statistical analysis showed that cell viability did not significantly differ from the control group (cells without any DMSO) at any of the solvent concentrations used (S2 Fig). As shown in Fig 4, compounds **5a-c**, **7f**, and **7g** were non-toxic at concentrations below 30 µg/mL, while derivatives **7a, 7b**, and **7e** were non-toxic at concentrations below 15.5 µg/mL. Only compound **7d** exhibited very slight cytotoxicity at concentrations below 3.75 µg/mL. Interestingly, some synthetic compounds increased cell viability at higher concentrations, likely due to stimulatory effects on cell proliferation or metabolism. Viability percentages exceeding 100% have also been reported in similar studies, indicating enhanced cellular health. These findings align with previous biological assays, providing a foundation for further investigations into the molecular mechanisms underlying the activity of the synthetic compounds [52,53].

### 3.4 Relative gene transcription by QPCR

**Fig 5:**
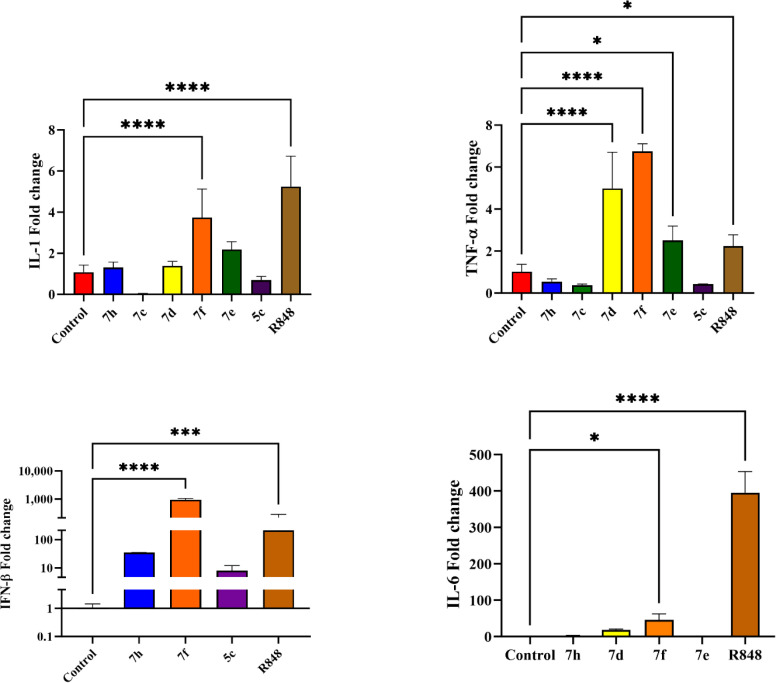
Release of cytokines TNF-α, IL-6, IL-1, and IFN-β from J774 macrophage cells exposed to synthetic compounds compared to the reference drug (R848). ****, ***, and * represent *p <* 0.0001, 0.001, and 0.05, respectively. Abbreviations; IL-1: Interleukin-1, TNF-α: Tumor necrosis factor, IFN-β: Interferonβ, IL-6: Interleukin-6, R848: Resiquimod.

Among the compounds that were non-toxic to the J774.1 macrophage cell line and displayed the highest binding energy in docking studies, six compounds were selected for evaluation of cytokines stimulation. As shown in Fig 5, the compound **7f**, which induced the highest production of IL-1, TNF-α, and IFN-β cytokines, has the potential to be a suitable candidate for immune system stimulation. Although compounds **7d** and **7e** ranked next, they also increased the expression of IL-1 and TNF-α. A noteworthy point is that compounds **7d**, **7f**, and **7e** increased TNF-α levels even more than R848. Another significant finding was the higher fold change in IFN-β for compound **7f** compared to R848. However, all tested compounds showed weak or no effect on IL-6 expression compared to R848. P*-*values < 0.05 were considered statistically significant in all tests.

## 4 Discussion

Two new structures, N-alkyl/aryl-[1,2,4]triazolo[4,3-a]quinoxalin-4-amine and N-(alkyl/aryl)-1-methyl-[1,2,4]triazolo[4,3-a]quinoxalin-4-amine, were synthesized, each featuring alkyl or aryl amine substituents on the triazoloquinoxaline scaffold. Prior to synthesis, molecular docking studies confirmed the lowest binding energy of these compounds with the active site of TLR7, a validated target for the development of potent and successful immune-modulating drug candidates. Key amino acids present in the active site of the TLR7 receptor include Asp555, Thr586, Leu557, Thr532, Tyr356, Val355, Phe408, and Ile585.

In general, quinoxaline derivatives have consistently attracted scientific interest due to their simple and cost-effective synthesis route, as well as their wide range of biological activities, such as anticancer [54,55], antimicrobial [56,57], antiparasitic [58], anti-inflammatory [59,60], anti-diabetic [61], and immunomodulatory [47] properties.

This study ultimately introduces the non-toxic triazoloquinoxaline derivative **7f**, which demonstrated low binding energy against TLR7 and the highest production of IL-1, TNF-α, and IFN-β cytokines, as a promising candidate for immune system stimulation. To some extent, it also increased IL-6 production. IL-1, IL-6, TNF-α, and IFN-β are among the most critical cytokines targeted in the development of new therapies. Accordingly, considerable efforts have been devoted to the development of new synthetic drugs capable of modulating these cytokines without causing undesirable side effects. The secretion of these cytokines by activating immune cells such as monocytes, macrophages, fibroblasts, and keratinocytes enhances immune responses, whereas insufficient cytokine secretion is associated with immune system suppression.

Various studies have demonstrated the effect of IMQ and R848 in inducing the innate immune system cytokines, including IFN-α/β, IL-1α/β, IL-1, IL-6, TNF-α, and others, in both *in vitro* and *in vivo* studies. By inducing these proinflammatory cytokines, these drugs indirectly stimulate both the innate and adaptive immune systems, showing significant antiviral and antitumor potential. Their main targets are macrophages, lymphocytes, and dendritic cells that express TLR7 and TLR8.

**Fig 6:**
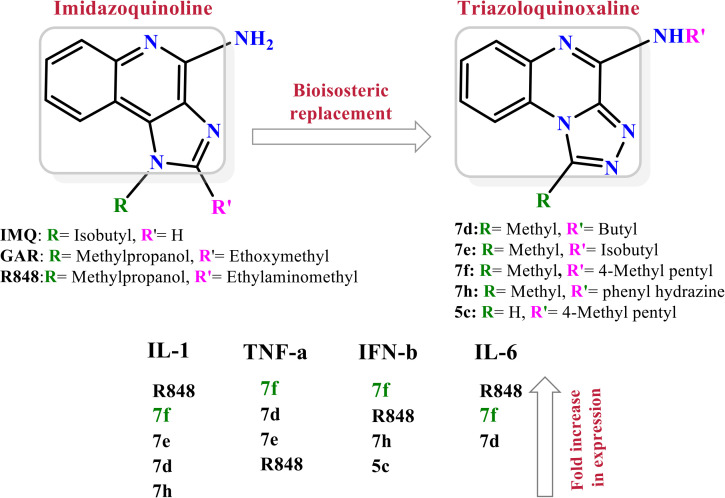
Comparison of the bioisosteric form of imidazoquinoline with triazolequinaxoline, and the structure- activity relationship of active synthetic compounds. Abbreviation; IMQ: imiquimod, R848: Resiquimod, and GAR: Gardiquimod.

Drugs targeting TLR7/TLR8 typically possess an imidazoquinoline structure, comprising an imidazole ring fused to a quinolinamide ring, with alkyl, ether and alcohol substitutions on the imidazole ring (Fig 1). Docking studies in this project revealed that replacing the imidazole ring with triazole and the quinolinamide with aminoquinoxaline improves the binding energy of the proposed compounds. In fact, imidazoquinoline and triazolequinoxaline are considered bioisosteres (Fig 6). Bioisosteric replacement typically results in new molecules with similar biological properties to the parent compound, while improving pharmacokinetics, potency, and function. Methyl substitution on the triazole ring, as well as alkylamine or phenylhydrazine substitutions on the quinoxaline ring, are other notable structural modifications compared to existing drugs. The strengths of this study include the simple synthesis routes, high yield, accessible reagents, easy purification, and cost-effective preparation of the proposed compounds based on docking studies.

Another strength of the triazoloquinaxoline derivatives **7d**-**f** synthesized in the present study is their ability to increase the expression of key proinflammatory cytokines, similar to imidazoquinoline drugs that target TLR7 (Fig 5). Among these, the effective compounds from series 7, with methyl substitution on the triazole ring and s-butyl, 4-methylpentyl, or isobutyl substitutions on the amine of the triazoloquinoxaline scaffold, with a binding energy of about −8.2 kcal/mol based on docking studies, were identified as the most potent stimulators of cytokine expression. In contrast, within series 5, which lacks substitution on the triazole ring, only compound **5c** showed a moderate fold change for IFN-β. Notably, compound **5c** also contains a 4-methylpentyl substitution on the amine of triazoloquinoxaline scaffold, similar to the potent compound **7f**. Therefore, it can be concluded that methyl substitution on the triazole ring and 4-methylpentyl substitution on the quinoxaline scaffold play critical roles in enhancing interaction with the TLR7 macromolecule target. As shown in the docking studies (Fig 6), the methyl substitution on the triazole ring in compounds of series 7 interacts with key amino acids in the active site of TLR7, including Tyr356A, Phe408A, Thr532B, Asp555B, Leu557B, Ile585B, and Thr586B (Fig 3 and S1 Fig). Compounds **5c** and **7f**, both bearing the 4-methyl pentyl substitution, exhibit common interactions with residues such as Phe351A, Gln354A, Val355A, Tyr356A, Phe408A, and Leu557B (Fig 3 and S1 Fig).

IL-1, one of the cytokines induced by triazoloquinoxaline in this study, belongs to the IL-1 superfamily, which comprises 11 structurally related cytokines secreted by various cell types, including macrophages, monocytes, dendritic cells, and epithelial cells, in response to defensive stimuli [62]. Among them, IL-1α and IL-1β, collectively referred to as IL-1, are the most prominent proinflammatory members. These cytokines are produced in response to different pathological conditions such as infections, tissue injury, photodamage, and other inflammatory stimuli. They play essential roles in the initiation and amplification of immune responses [63].

Both IL-1α and IL-1β bind to the interleukin-1 receptor type 1 (IL-1R1), forming a signaling complex that activates downstream pathways involved in both innate and adaptive immunity [64]. This interaction promotes the production of other inflammatory mediators such as IL-6, TNF-α, and IL-8, and facilitates the recruitment of immune cells including neutrophils, macrophages, T cells, and NK cells to sites of infection or tissue damage, thereby contributing to inflammation and tissue repair [63,65]. Due to its crucial role in inflammation, IL-1 is increasingly considered as an attractive therapeutic target for a broad spectrum of diseases, such as autoimmune and autoinflammatory diseases, infections, metabolic disorders, ischemic conditions, and malignant tumors [66].

TNF-α is another critical multifunctional cytokine that regulates host defense, immune responses, inflammation, and apoptosis. It induces inflammation by promoting gene transcription mainly through the NF-κB and AP-1 signaling pathways, leading to the expression of numerous proinflammatory genes. TNF-α plays a vital role in innate immunity in host defense against fungal, bacterial, viral, and parasitic pathogens, particularly against intracellular organisms like *Mycobacterium tuberculosis*, through the recruitment and activation of macrophages, NK cells, T cells, and antigen-presenting cells [67,68]. Notably, synthetic compounds **7d** and **7f** increased TNF-α expression more effectively than R848.

IFN-β, another cytokine upregulated by triazoloquinoxaline compounds in this study, belongs to the family of type I IFNs secreted by various cells, including macrophages, fibroblasts, endothelial cells, osteoblasts, and DCs [69,70]. Upon binding to interferon receptors (IFNAR), IFN-β initiates a signaling cascade via the Janus kinase/signal transducer and activator of transcription (JAK/STAT) pathway. This leads to the phosphorylation of STAT1 and STAT2, which then form a complex with IRF9 to create Interferon-stimulated gene factor 3 (ISGF3). This complex binds to IFN-stimulated response elements (ISREs), thereby promoting the transcription of ISGs [71]. The resulting ISGs produce antiviral, antibacterial, antitumor, immunomodulatory, and anti-inflammatory proteins that activate immune cells and enhance cytokine expression in response to pathogens [72,73].

Interestingly, IFN-β influence both innate and adaptive immune responses, increasing pathogen clearance and promoting protective immunity against cancers and infections. IFN-β was approved by the U.S. Food and Drug Administration (FDA) in 1993 as the first immunomodulatory treatment for multiple sclerosis [74]. One study showed that IFN-β reduced LPS-stimulated IL-12 secretion while increased IL-10 secretion in Galectin-1-deficient macrophages, without significantly influencing TNF-α and IL-6 levels, suggesting a potential role in resolving inflammation and limiting excessive inflammatory activity [75].

The ability of triazoloquinoxaline derivatives to stimulate the immune system without inducing cytotoxicity in macrophages, even at high concentrations, makes them promising candidates for applications in skin malignancies, viral infections, cancer, and as adjuvants. Several studies have shown the synergistic effects of proinflammatory cytokines in the treatment of various diseases [76,77]. It is assumed that IL-1, TNFα, and IFN-β may also synergistically promote immune responses, thereby improving treatment outcomes, especially in cancer and infectious diseases. Furthermore, the combination therapy of new triazoloquinoxaline amine derivatives with other therapeutic agents is recommended. This approach is inspired by previous studies that have introduced combination therapy of IMQ with various treatments, including anticancer drugs [78–80], antibodies [81], treatment for skin disorders [82,83], antiparasitic agents [84], and antifungal medications [85], aimed to enhancing IMQ efficacy while reducing side effects.

## 5 Conclusion

A new series of imidazoquinoline analogs targeting TLR7, featuring a main core structure of methyl[1,2,4]triazolo[4,3-a]quinoxaline with branched alkyl amine substitutions on the triazole ring, exhibited strong binding affinity to the active site of TLR7 based on molecular docking studies. These derivatives were synthesized using green chemistry and sustainable processing methods, employing simple and cost-effective procedures. This approach eliminates the need for complex purification steps and results in high yields, making it potentially suitable for industrial-scale production. The non-toxic triazoloquinoxaline derivatives effectively stimulated IL-1, TNF-α, and IFN-β secretion in an *in vitro* macrophage cell model. Compound **7f**, which contains a 4-methylpentanyl amine moiety on the quinoxaline scaffold of methyl[1,2,4]triazolo[4,3-a]quinoxaline, showed particularly promising results and could be considered a viable alternative to imidazoquinolines for TLR7 targeting. The findings of this study may be of interest to the pharmaceutical industry and could serve as a starting point for the development of novel, potent and safe drugs based on the triazoloquinoxaline scaffold. However, further studies are needed to fully validate and substantiate these results, which should be included in future research.

## Supporting information

S1 Fig Two-dimensional interactions of selected compounds with the TLR7 protein based on docking studies. (PDF)

S2 Fig Cells exposed to different concentrations of DMSO in the MTT assay. (PDF)

S3 Fig Spectral Information of the compounds, including ^1^H-NMR, ^13^C-NMR, FT-IR, CHN analysis, HRMS-ESI. (PDF)
