# Supplementary figures and images for: Identification of new triazoloquinoxaline amine derivatives with potent modulatory effects against Toll-like receptor 7 through pharmacophore-based virtual screening and molecular docking approaches
Source: PLoS One. 2025 Dec 29;20(12):e0336701. doi: 10.1371/journal.pone.0336701 (PMC12747431; doi:10.1371/journal.pone.0336701)

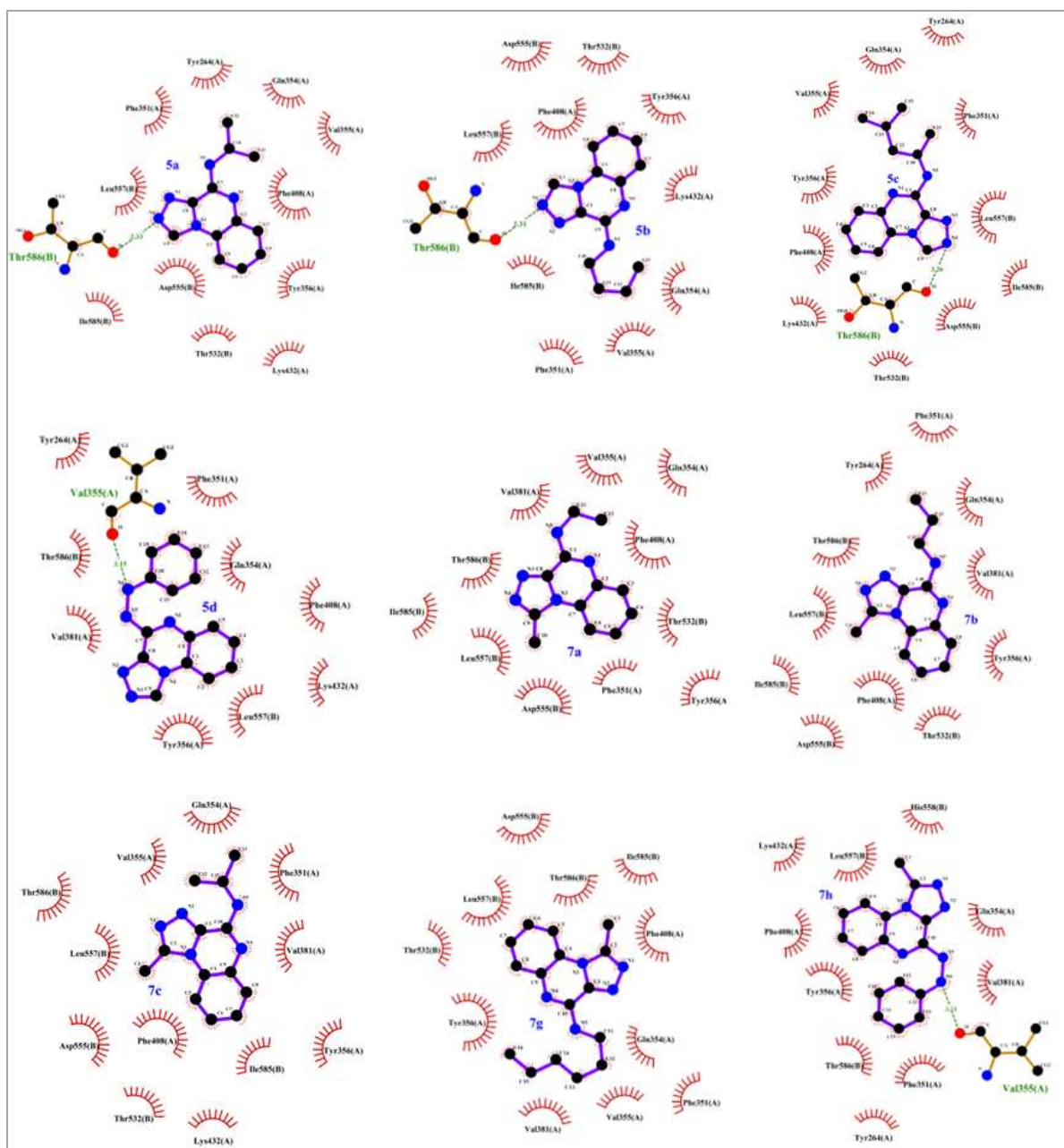

Supplement: S1 Fig — (PDF) [file pone.0336701.s001.pdf]

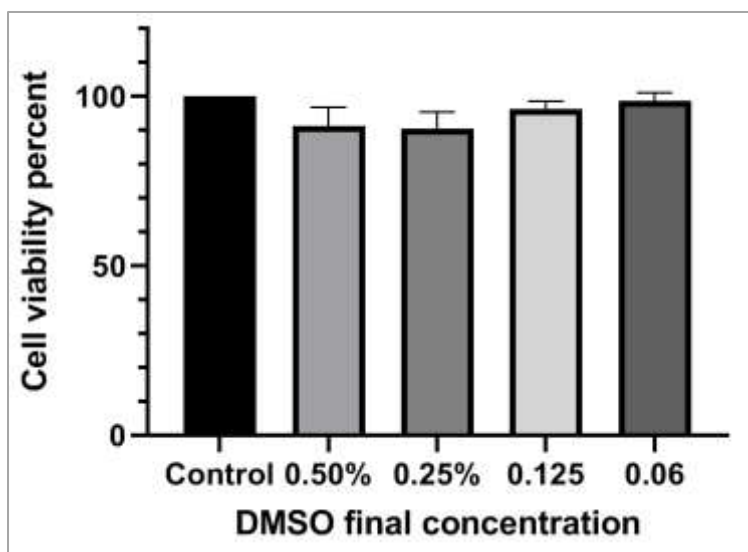

Supplement: S2 Fig — (PDF) [file pone.0336701.s002.pdf]
