# Supplementary material for: Identification of new triazoloquinoxaline amine derivatives with potent modulatory effects against Toll-like receptor 7 through pharmacophore-based virtual screening and molecular docking approaches
Source: PLoS One. 2025 Dec 29;20(12):e0336701. doi: 10.1371/journal.pone.0336701 (PMC12747431; doi:10.1371/journal.pone.0336701)
Supplement: S3 Fig — (PDF) [file pone.0336701.s003.pdf]

N-butyl-[1,2,4]triazolo[4,3-a]quinoxalin-4-amine:

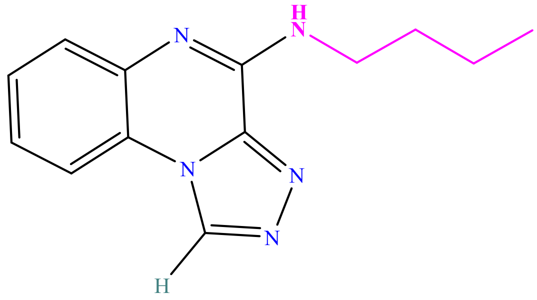

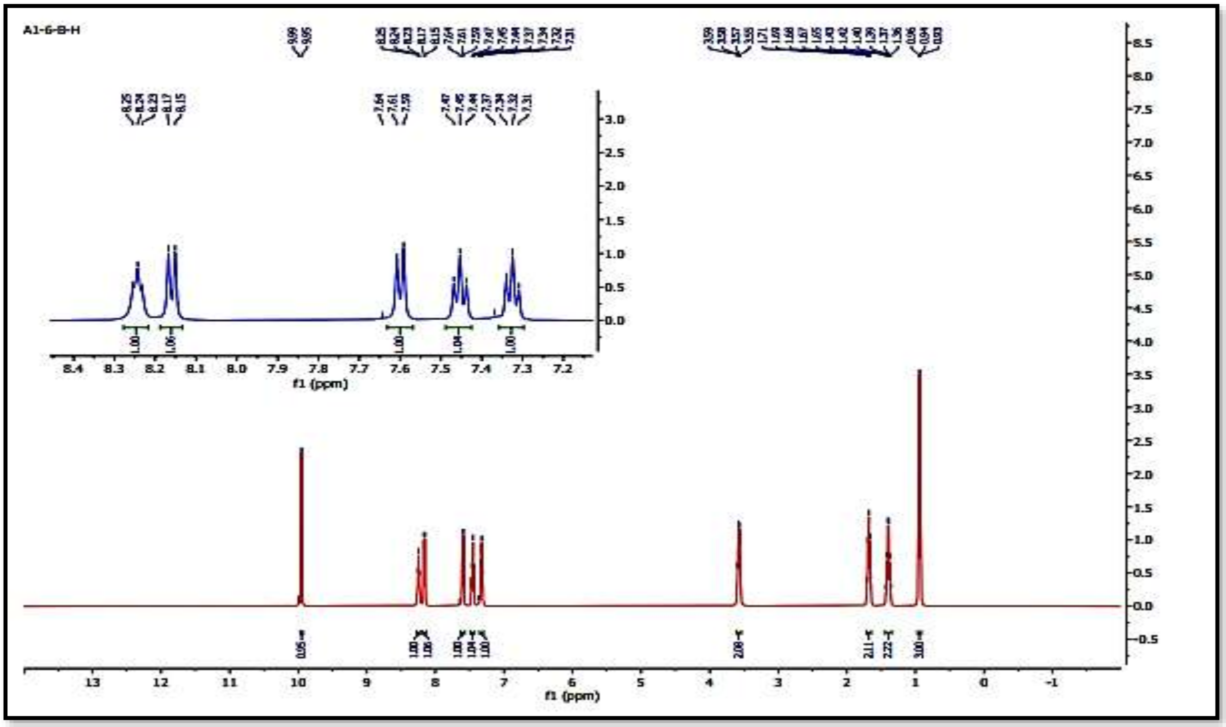

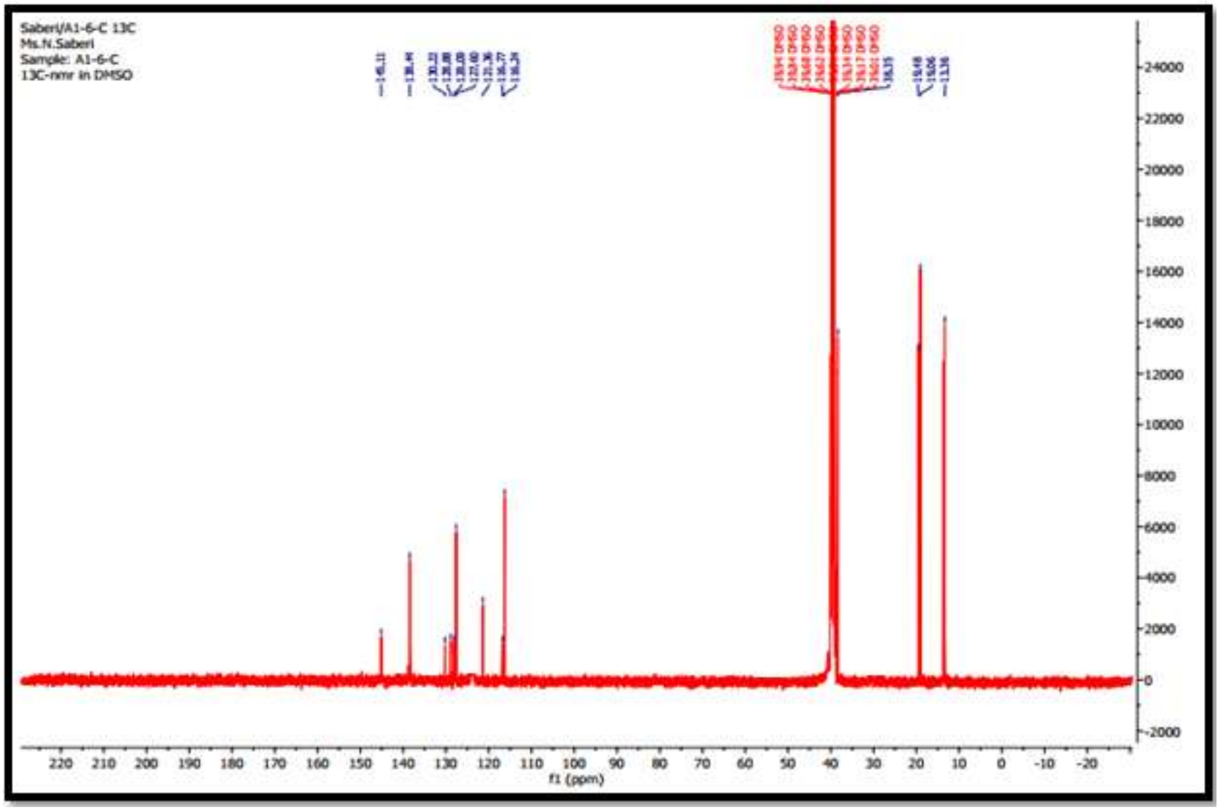
Saberi/A1-6-C 13C
Ms.N.Saberi
Sample: A1-6-C
13C-nmr in DMSO
f1 (ppm)

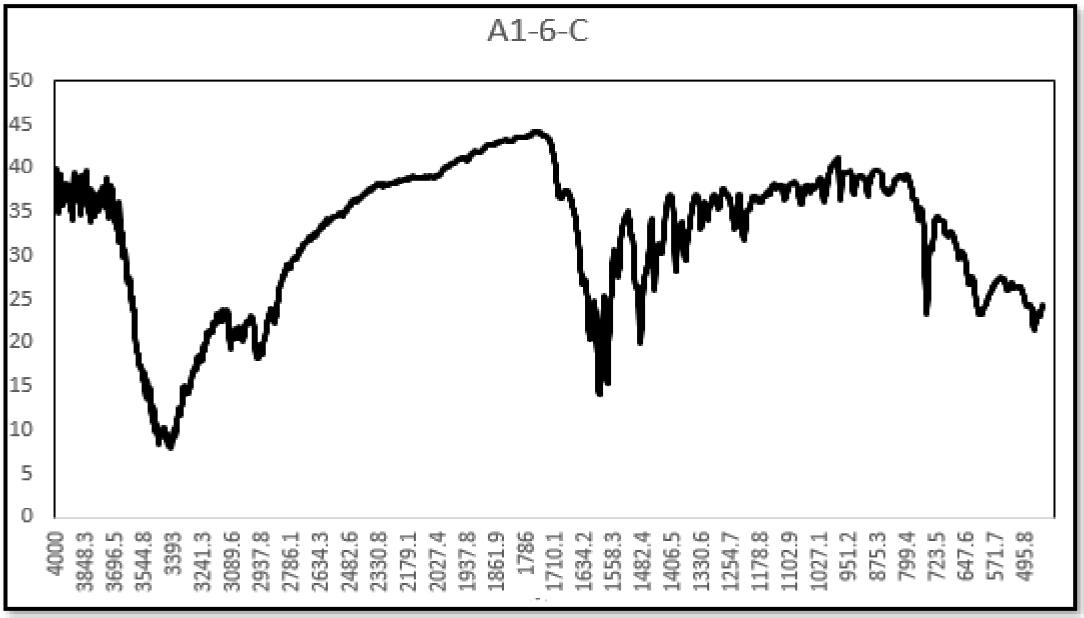
A1-6-C

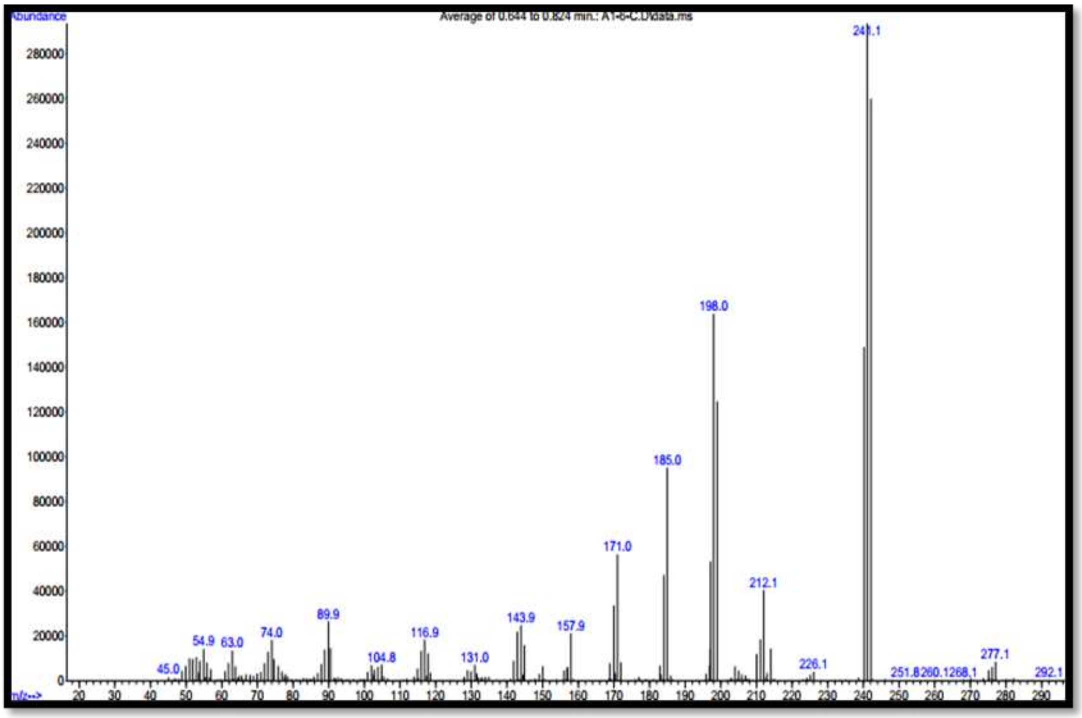

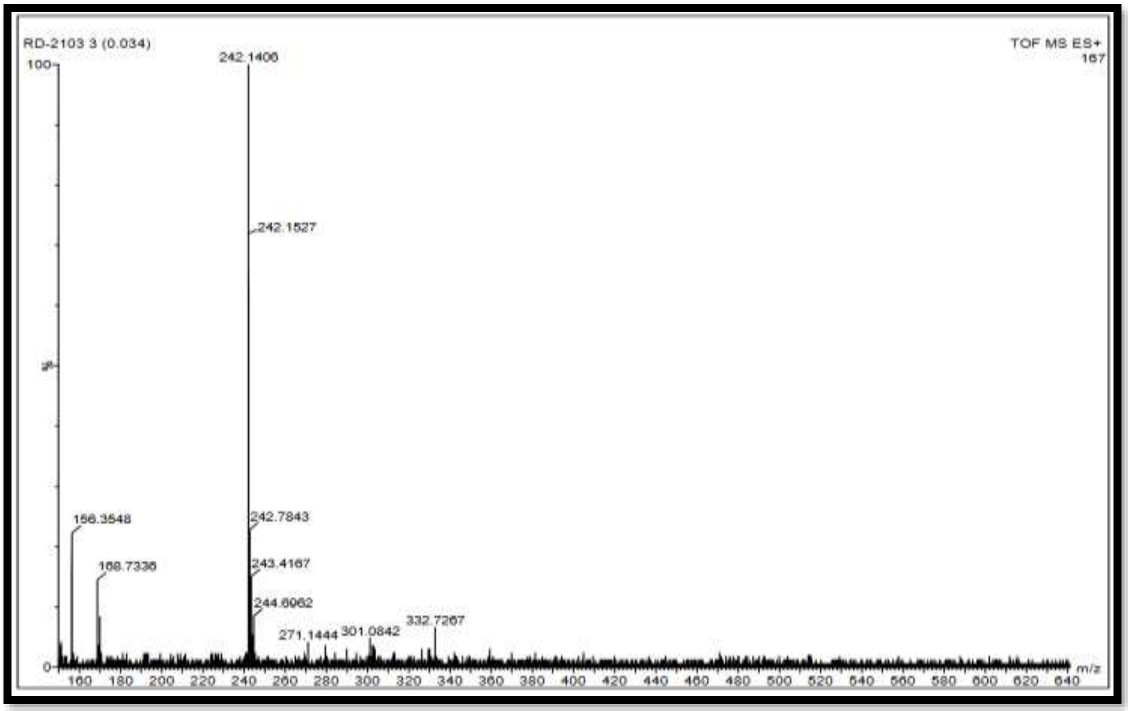

N-isopropyl-[1,2,4]triazolo[4,3-a]quinoxalin-4-amine:

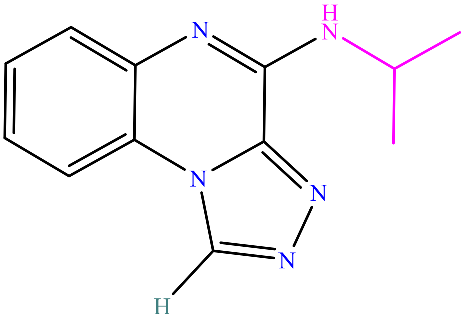

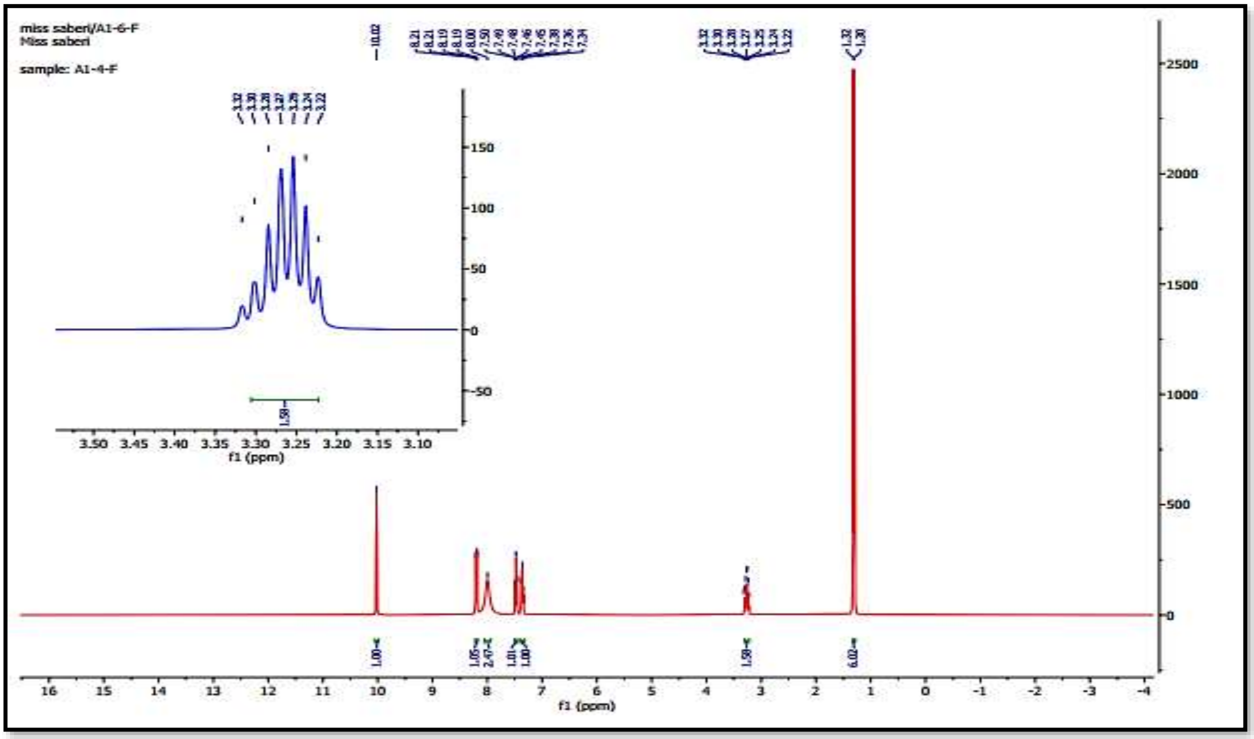

miss saberi/A1-6-F
Miss saberi
sample: A1-4-F
10.02
1.32
1.30
f1 (ppm)

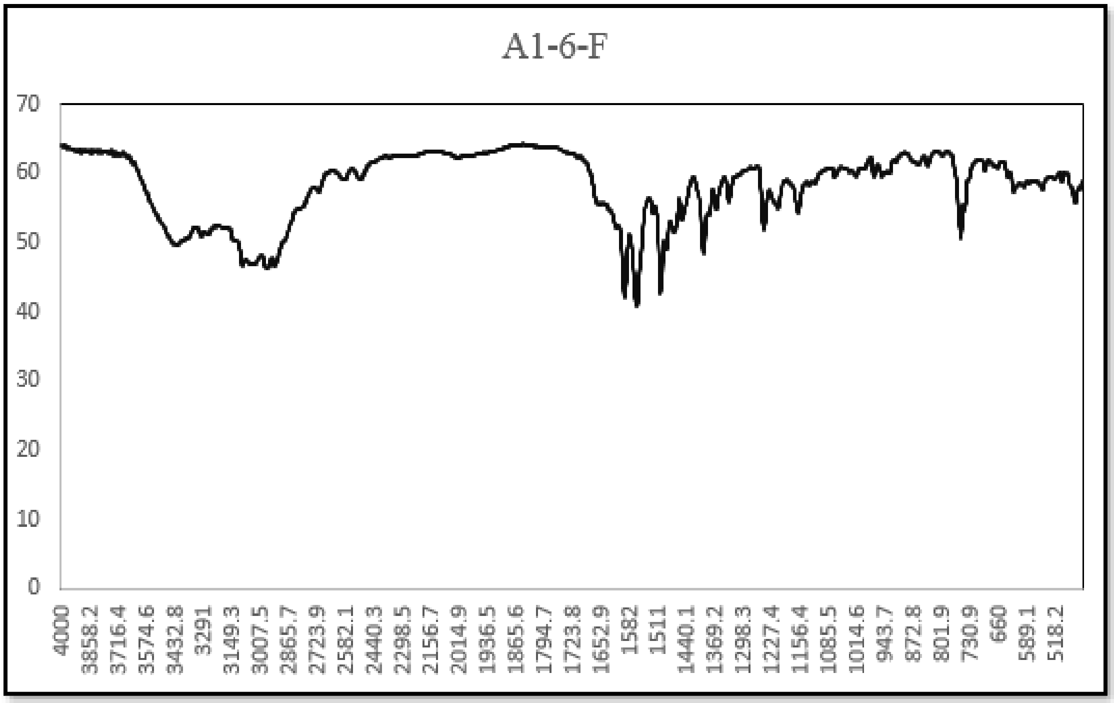
A1-6-F
70
60
50
40
30
20
10
0
4000
3858.2
3716.4
3574.6
3432.8
3291
3149.3
3007.5
2865.7
2723.9
2582.1
2440.3
2298.5
2156.7
2014.9
1936.5
1865.6
1794.7
1723.8
1652.9
1582
1511
1440.1
1369.2
1298.3
1227.4
1156.4
1085.5
1014.6
943.7
872.8
801.9
730.9
660
589.1
518.2

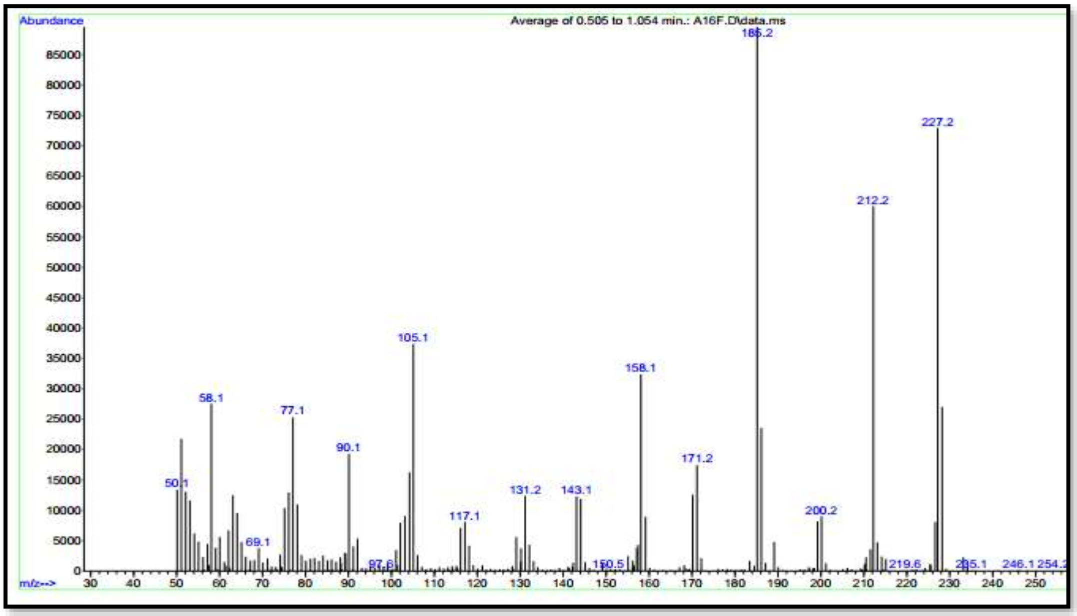
Abundance
Average of 0.505 to 1.054 min.: A16F.D\data.ms
185.2
227.2
212.2
105.1
158.1
58.1
77.1
90.1
171.2
50.1
131.2
143.1
117.1
200.2
69.1
97.6
150.5
219.6
246.1
254.2
m/z-->

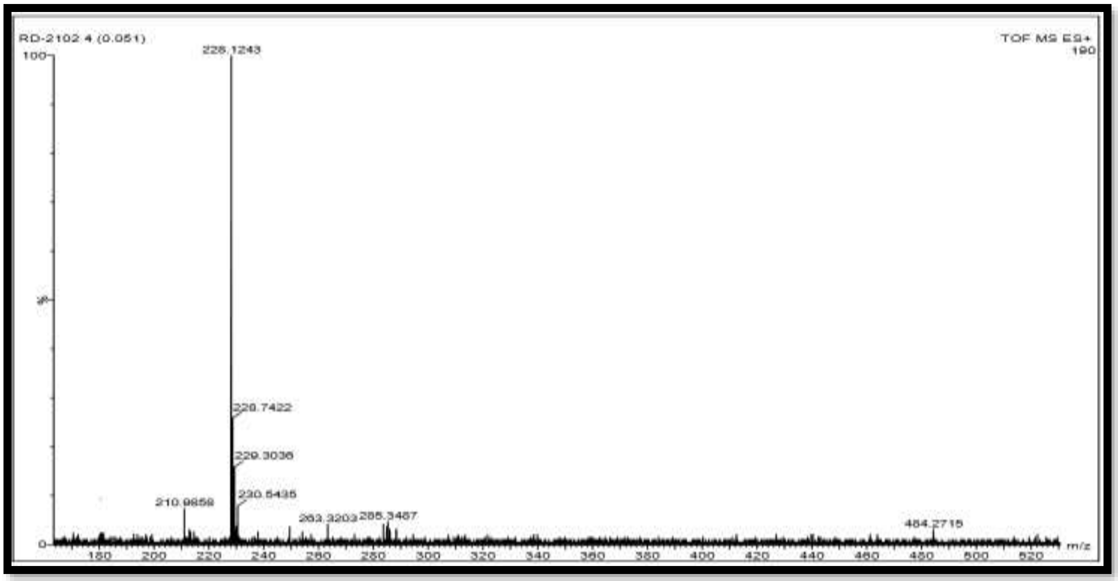

N-(sec-butyl)-1-methyl-[1,2,4]triazolo[4,3-a]quinoxalin-4-amine:

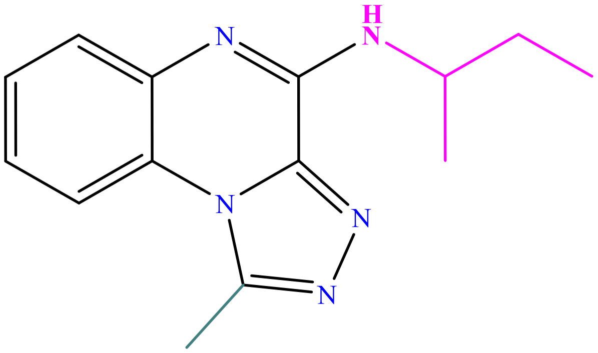

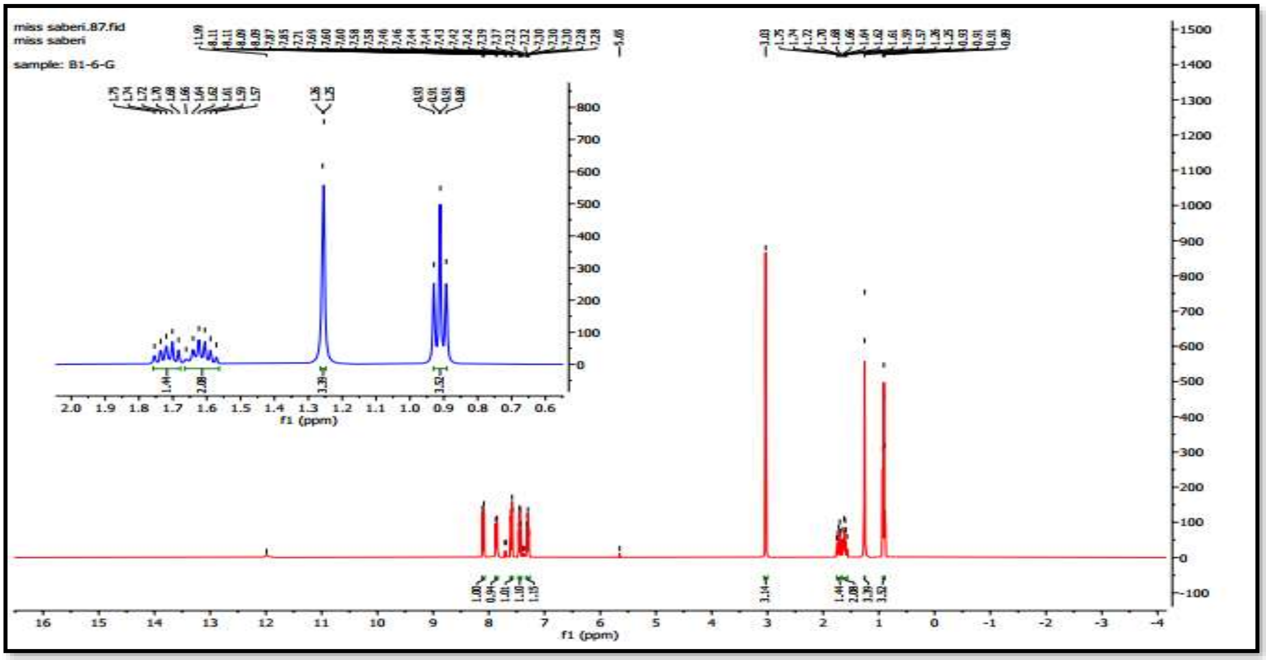
miss saberi.87.fid
miss saberi
sample: B1-6-G
f1 (ppm)

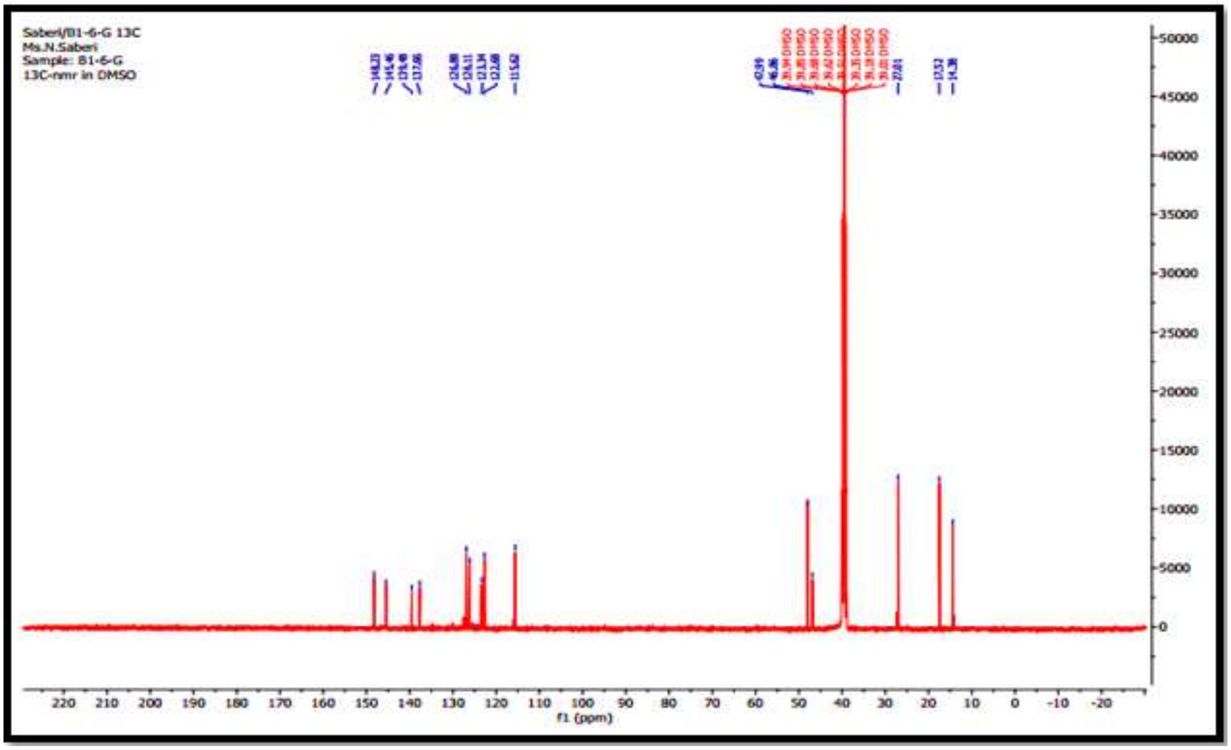
Saberi/B1-6-G 13C
Ms.N.Saberi
Sample: B1-6-G
13C-nmr in DMSO
f1 (ppm)

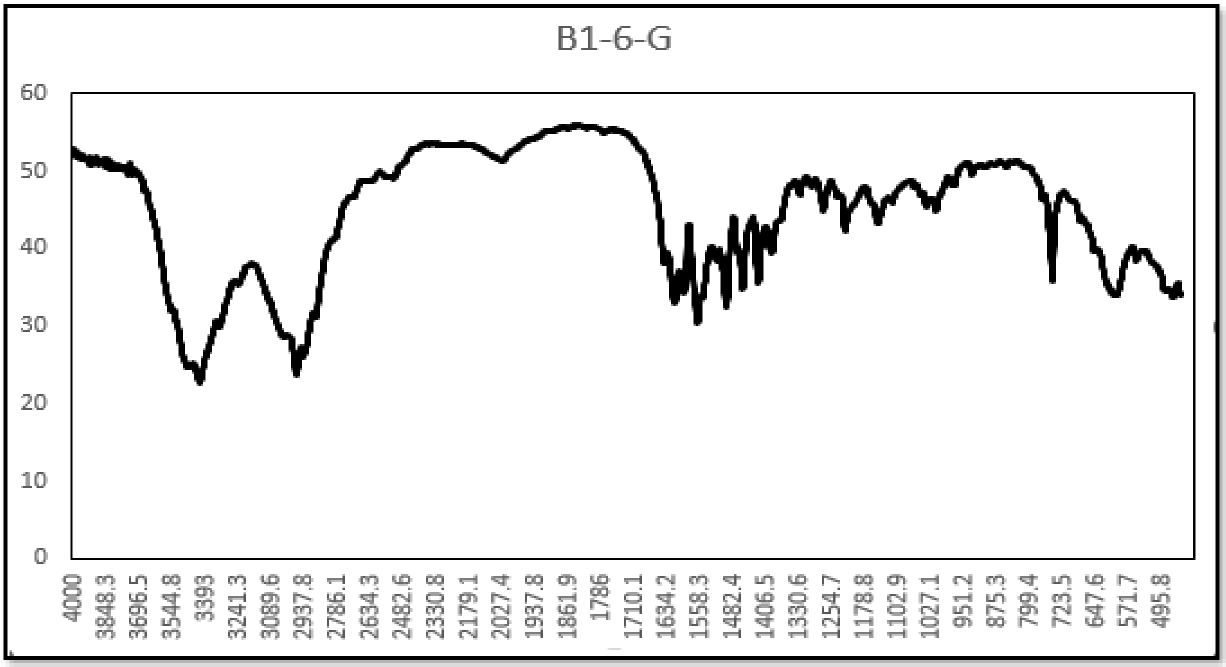
B1-6-G
60
50
40
30
20
10
0
4000
3848.3
3696.5
3544.8
3393
3241.3
3089.6
2937.8
2786.1
2634.3
2482.6
2330.8
2179.1
2027.4
1937.8
1861.9
1786
1710.1
1634.2
1558.3
1482.4
1406.5
1330.6
1254.7
1178.8
1102.9
1027.1
951.2
875.3
799.4
723.5
647.6
571.7
495.8

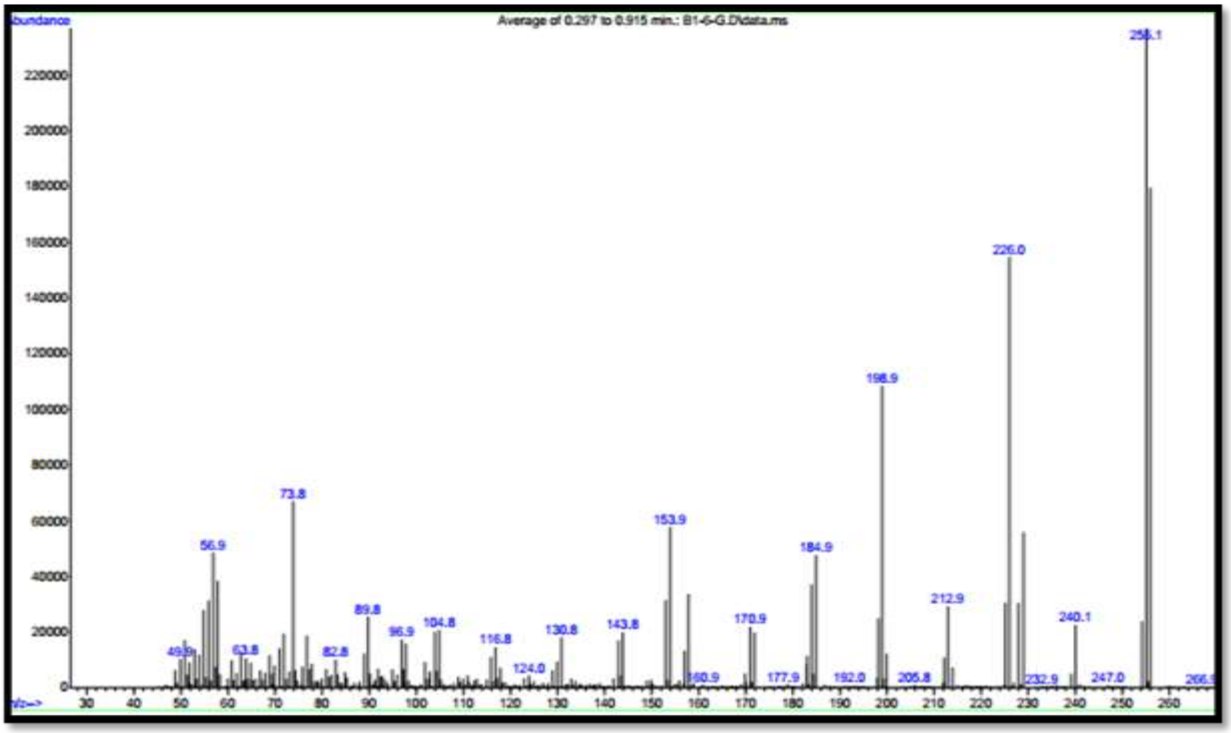
Abundance
Average of 0.297 to 0.915 min.: B1-6-G.D\data.ms
220000
200000
180000
160000
140000
120000
100000
80000
60000
40000
20000
0
m/z-->
226.0
198.9
73.8
153.9
56.9
184.9
212.9
89.8
170.9
240.1
143.8
104.8
96.9
130.8
116.8
49.9
63.8
82.8
124.0
160.9
177.9
192.0
205.8
232.9
247.0

Result Table (ESTD - E:\results\140406\140406-Saberi\14040605-Saberi-B16G - INT7 - 1)

	Reten. Time [min]	Response	Weight [mg]	Weight [%]	Peak Type	Element Name	Carbon Response Ratio
1	1.083	3642.029	1.413	27.63	Ordnr	N	???
2	1.780	22233.251	3.368	65.86	Ordnr	C	???
3	8.497	4649.446	0.333	6.51	Ordnr	H	???
	Total		5.114	100.00			

N-hexyl-1-methyl-[1,2,4]triazolo[4,3-a]quinoxalin-4-amine:

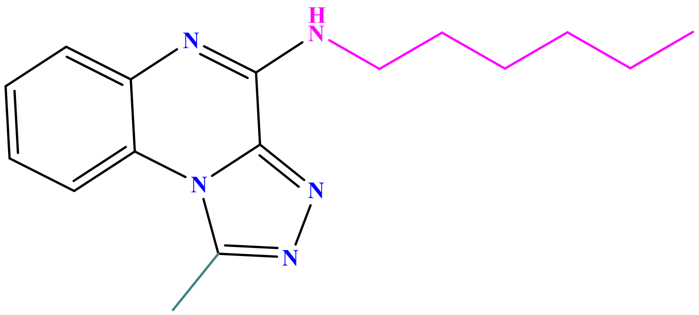

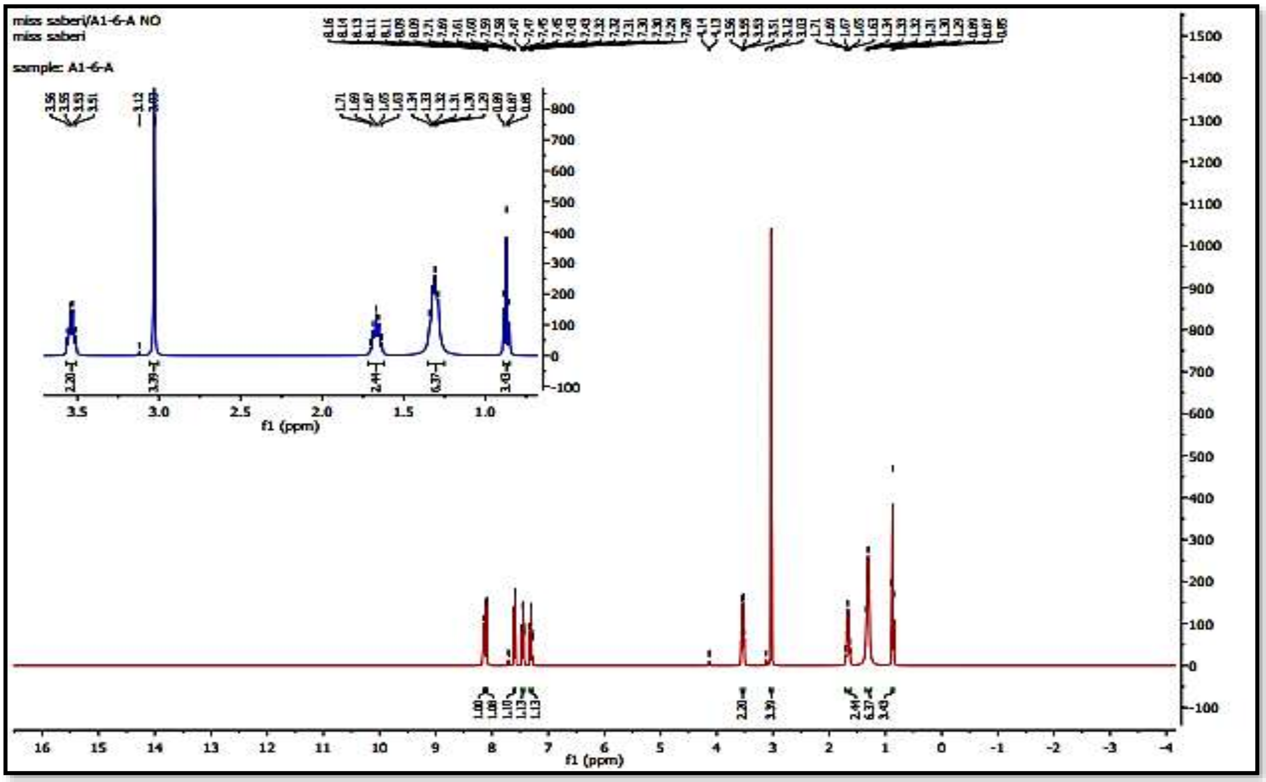

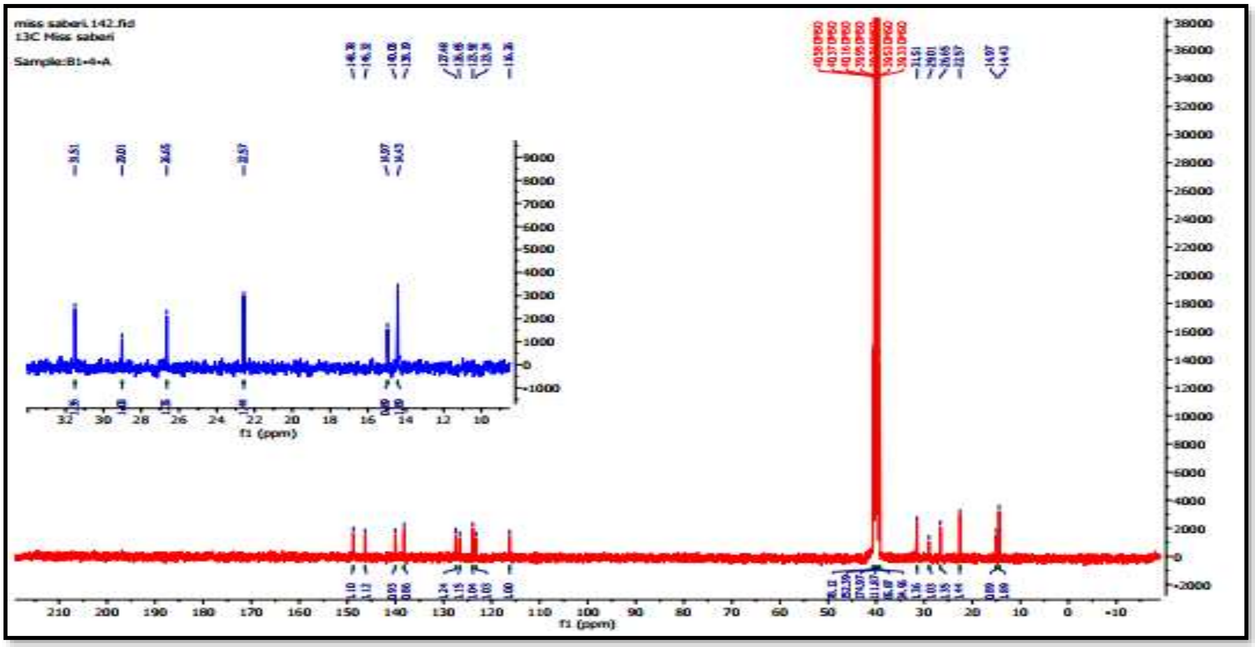

miss saberi.142.fid
13C Miss saberi
Sample:B1-4-A
f1 (ppm)

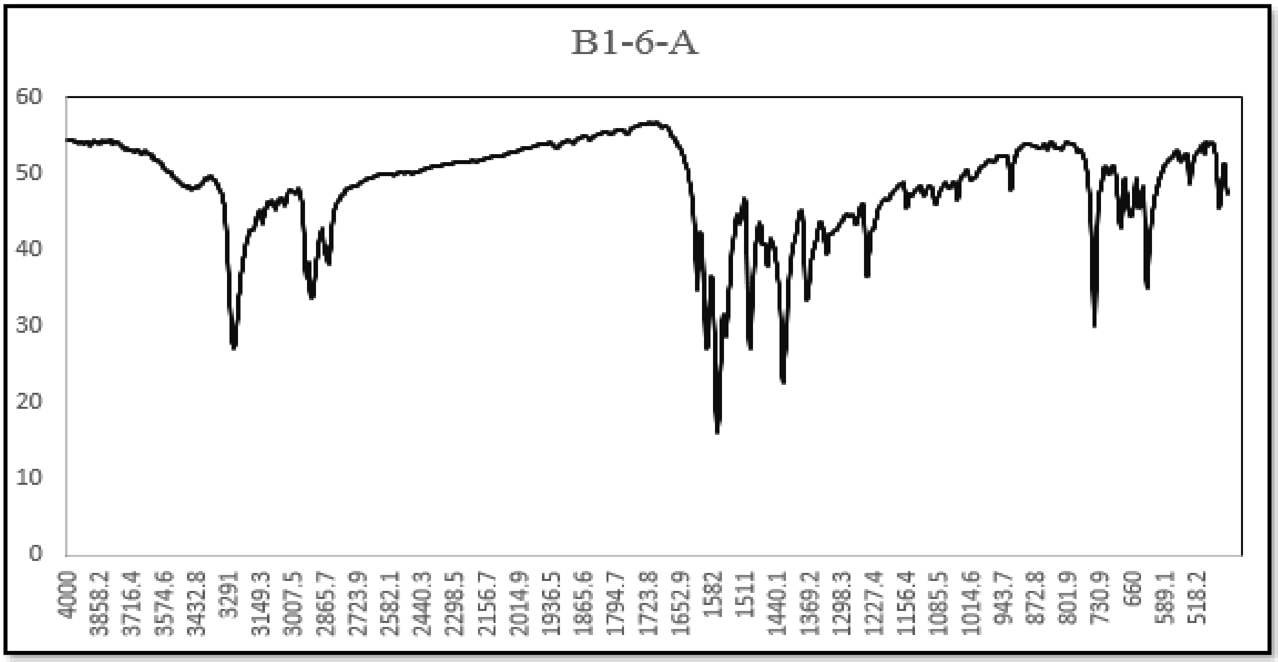

B1-6-A

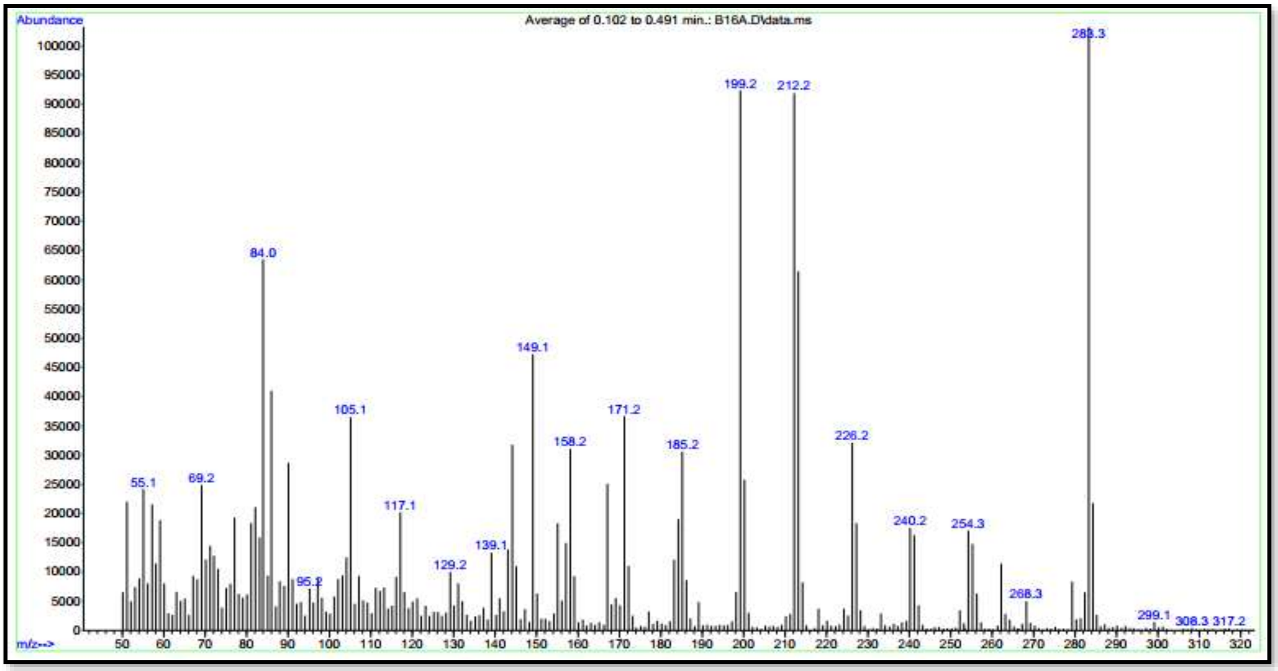

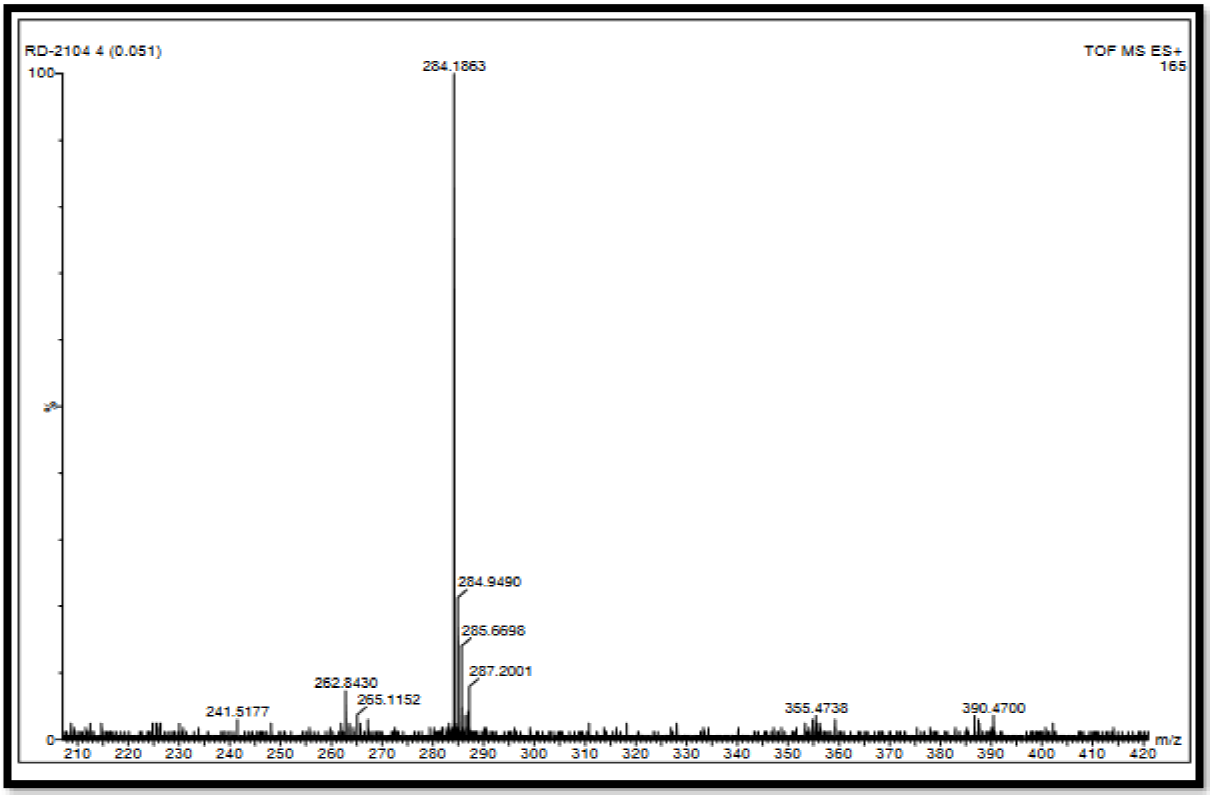

N-ethyl-1-methyl-[1,2,4]triazolo[4,3-a]quinoxalin-4-amine:

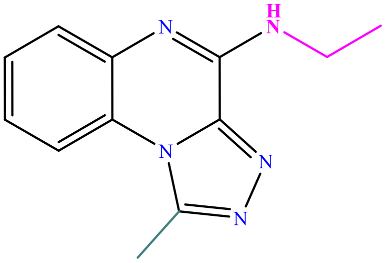

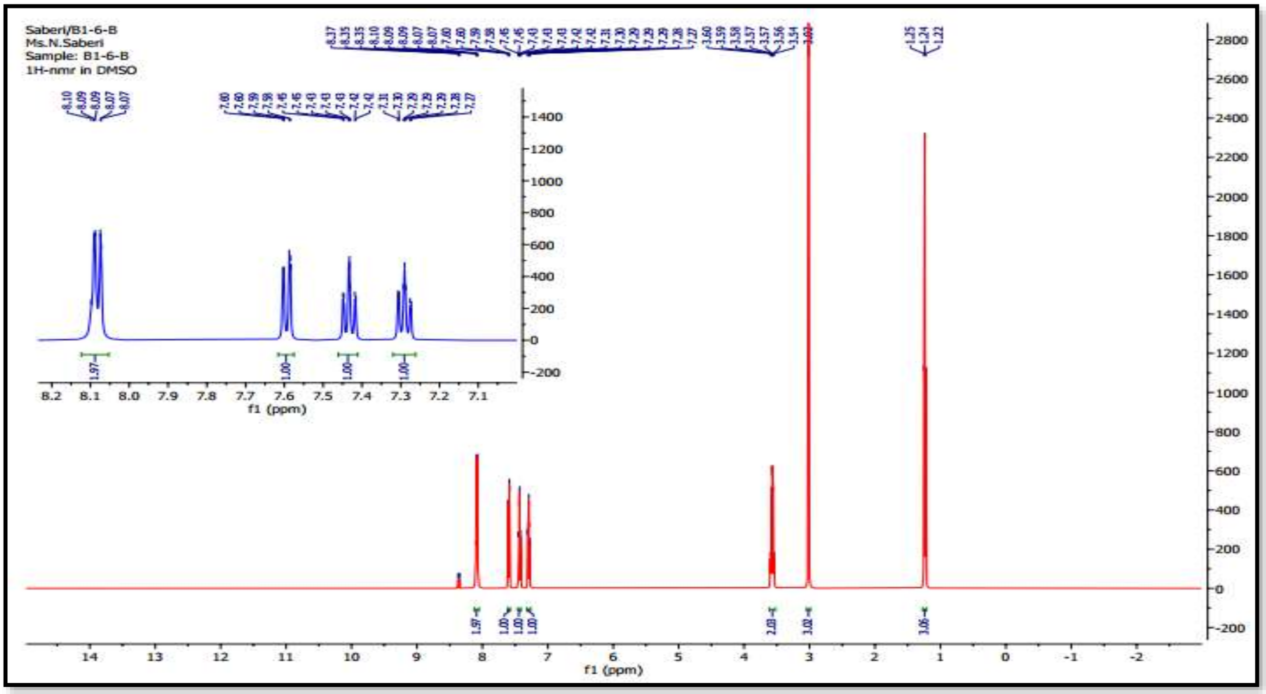
Saberi/B1-6-B
Ms.N.Saberi
Sample: B1-6-B
1H-nmr in DMSO
f1 (ppm)
f1 (ppm)

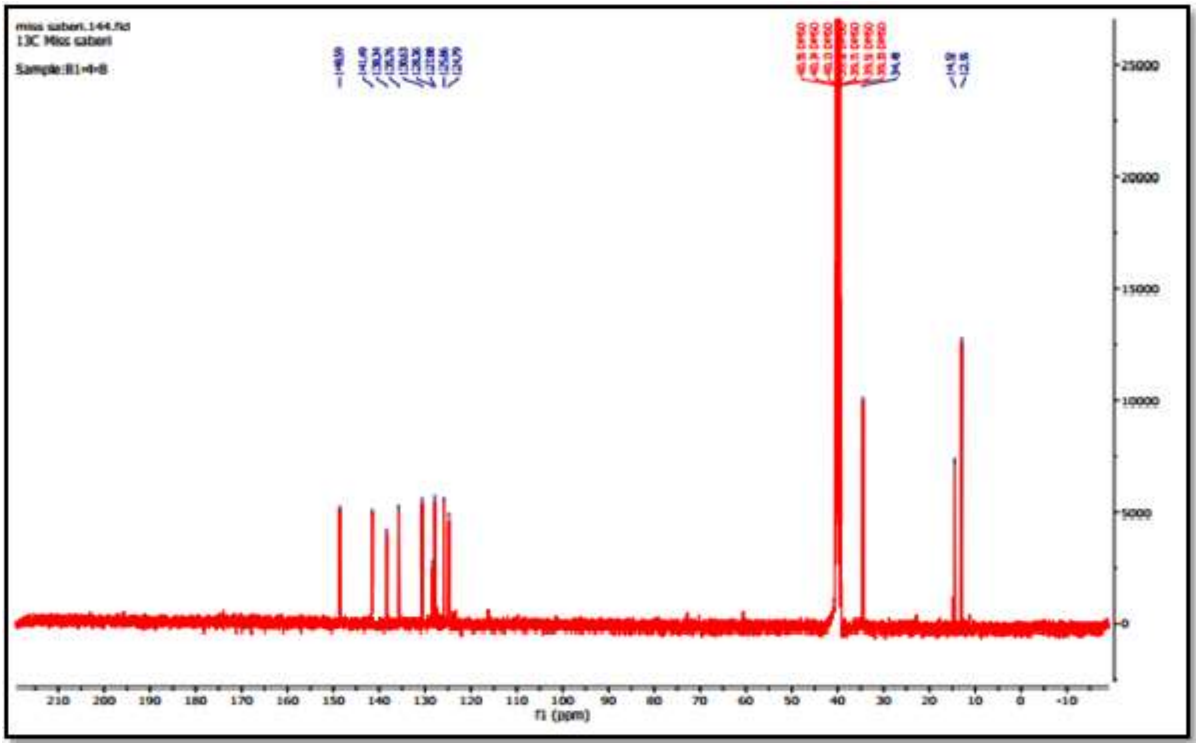
13C Miss saberi
f1 (ppm)

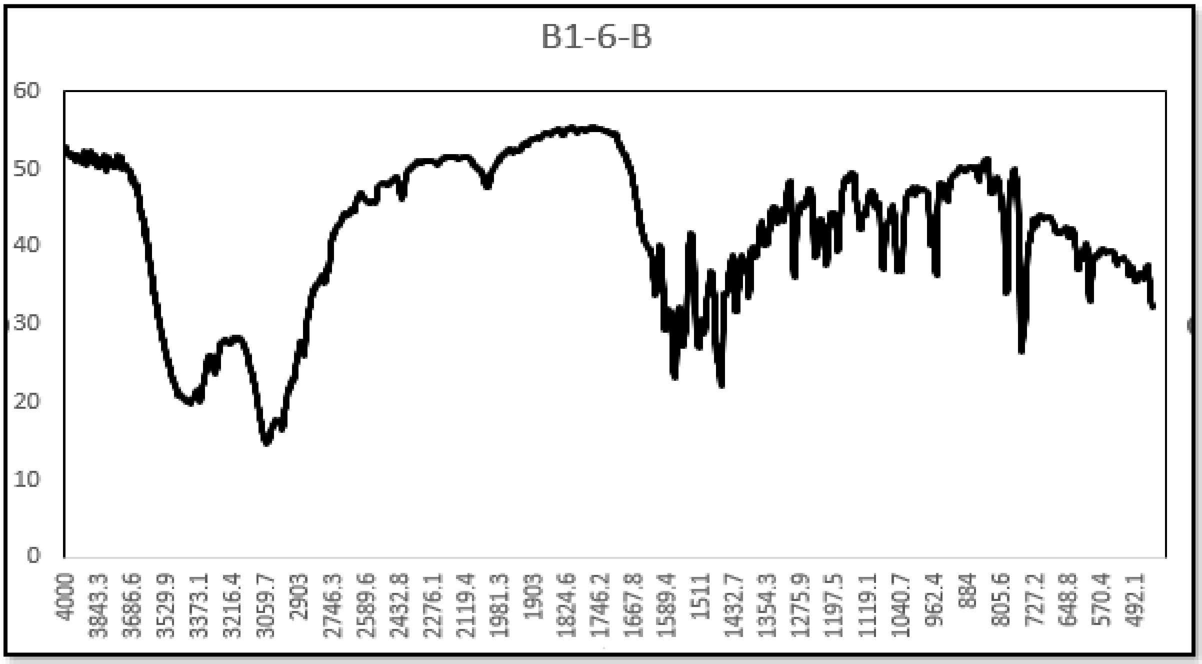
B1-6-B
60
50
40
30
20
10
0
4000
3843.3
3686.6
3529.9
3373.1
3216.4
3059.7
2903
2746.3
2589.6
2432.8
2276.1
2119.4
1981.3
1903
1824.6
1746.2
1667.8
1589.4
1511
1432.7
1354.3
1275.9
1197.5
1119.1
1040.7
962.4
884
805.6
727.2
648.8
570.4
492.1

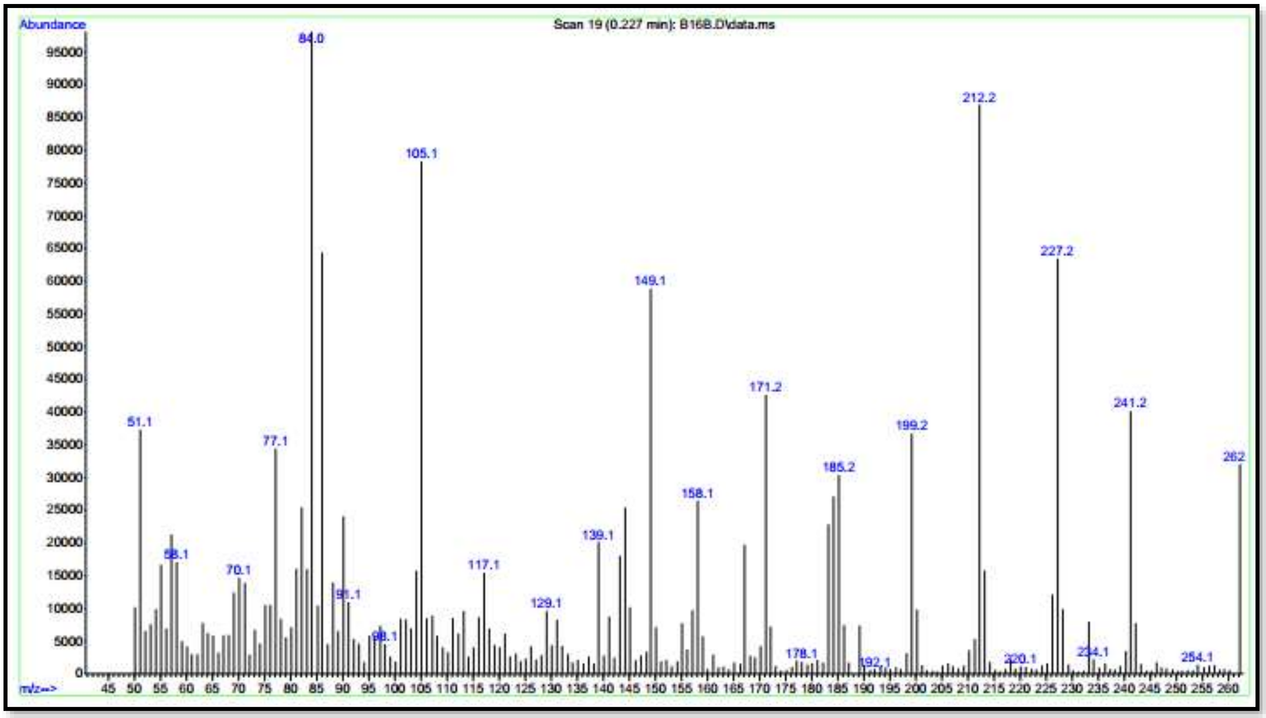

1-Methyl-N-propyl-[1,2,4]triazolo[4,3-a]quinoxalin-4-amine:

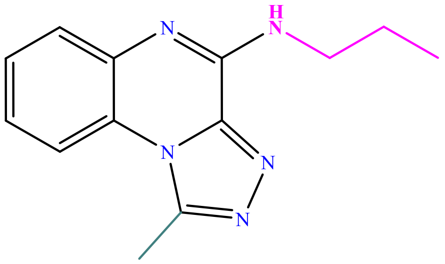

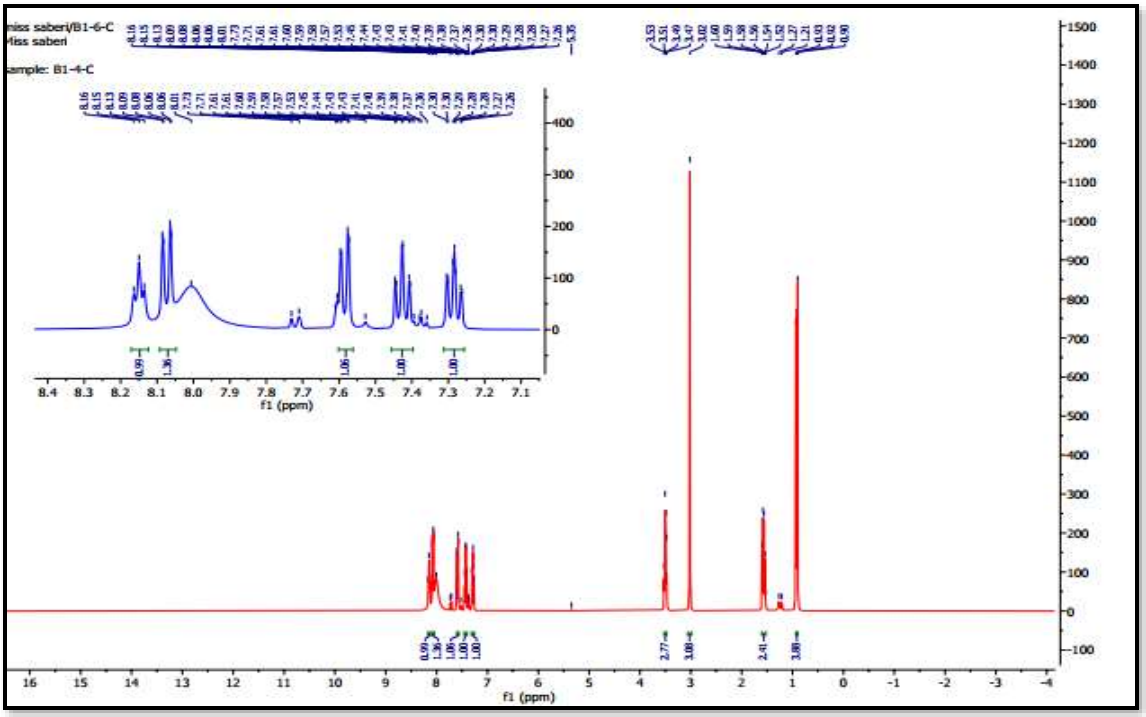
miss saberi/B1-6-C
Miss saberi
Sample: B1-4-C
f1 (ppm)

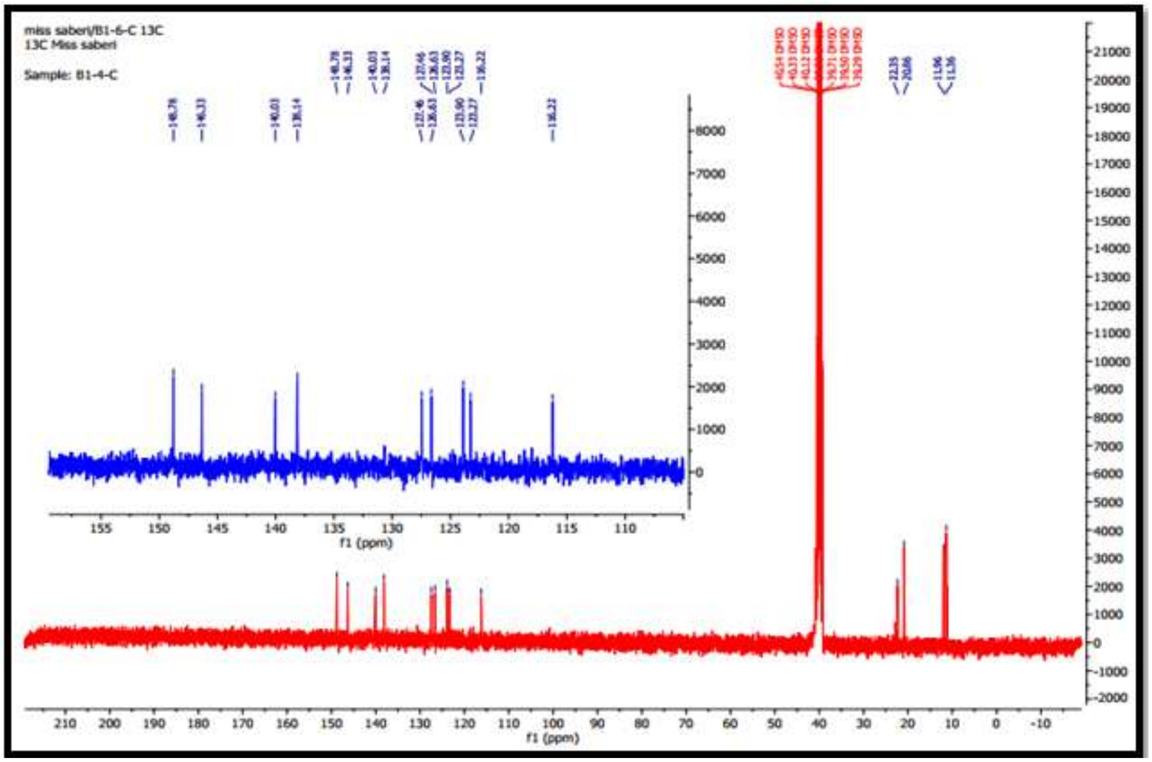
miss saberi/B1-6-C 13C
13C Miss saberi
Sample: B1-4-C
f1 (ppm)

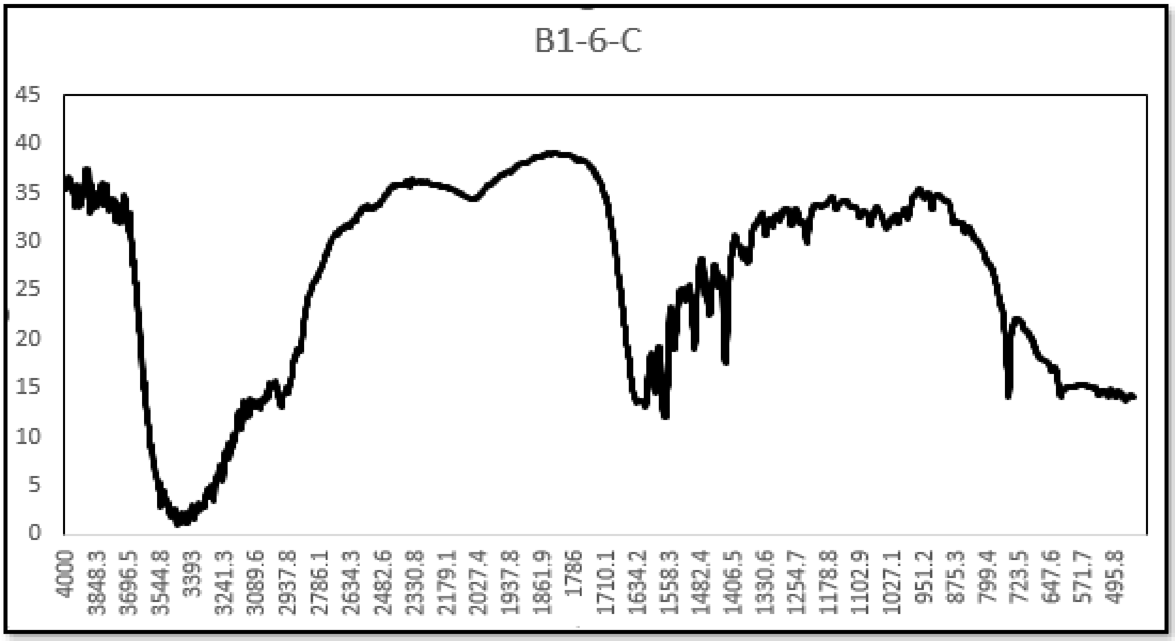
B1-6-C
45
40
35
30
25
20
15
10
5
0
4000
3848.3
3696.5
3544.8
3393
3241.3
3089.6
2937.8
2786.1
2634.3
2482.6
2330.8
2179.1
2027.4
1937.8
1861.9
1786
1710.1
1634.2
1558.3
1482.4
1406.5
1330.6
1254.7
1178.8
1102.9
1027.1
951.2
875.3
799.4
723.5
647.6
571.7
495.8

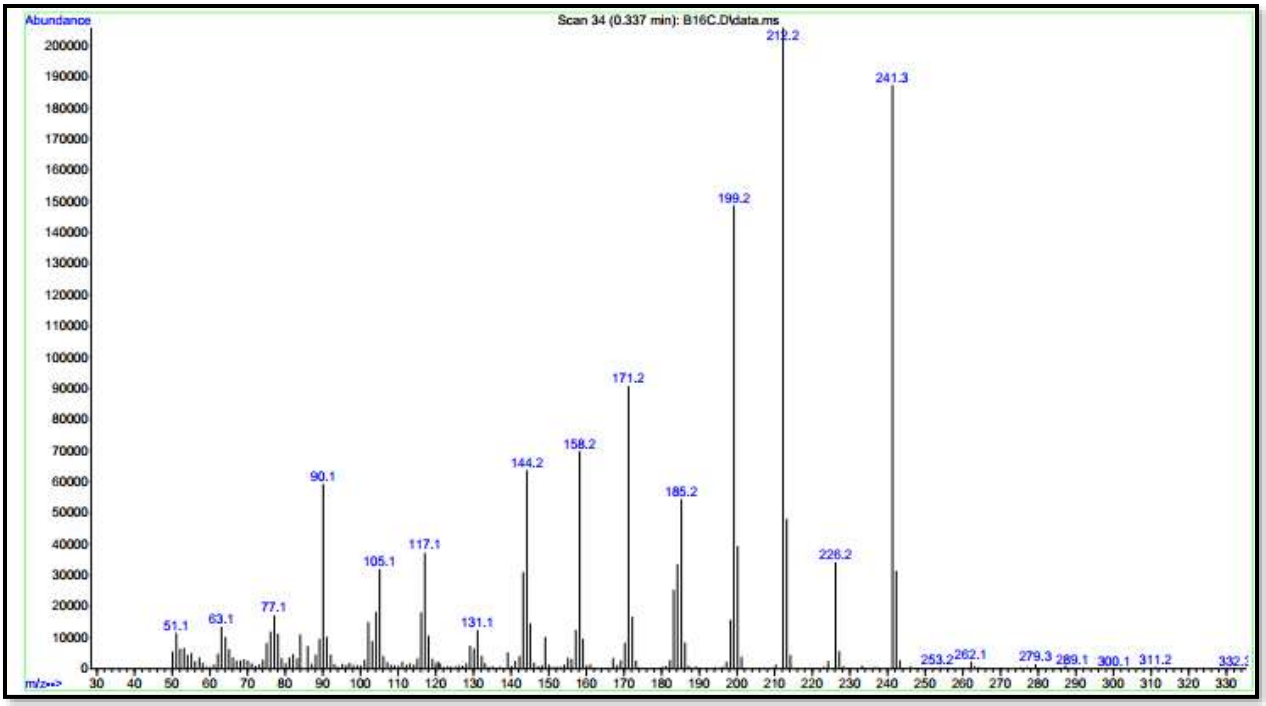
Abundance
Scan 34 (0.337 min): B16C.D\data.ms
212.2
241.3
199.2
171.2
158.2
144.2
185.2
90.1
117.1
105.1
226.2
77.1
63.1
51.1
131.1
253.2
262.1
279.3
289.1
300.1
311.2
m/z-->

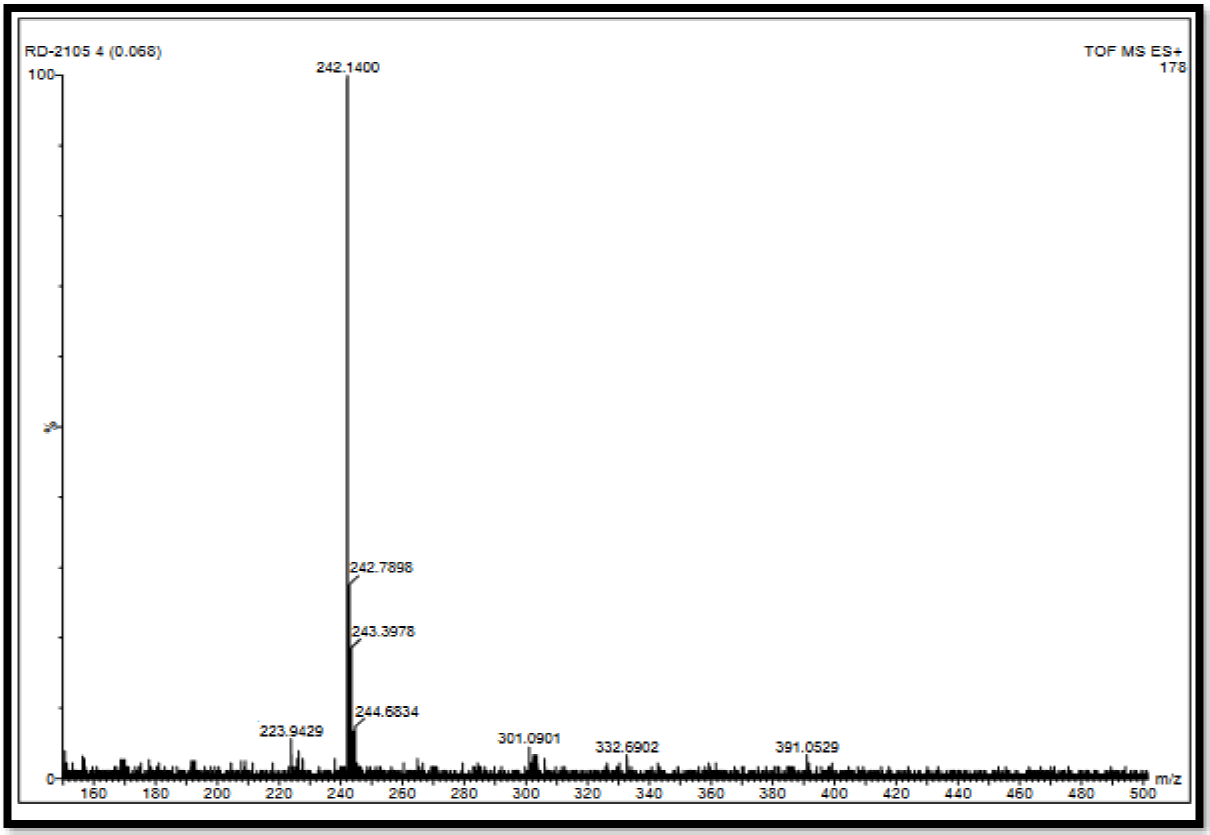

N-isobutyl-1-methyl-[1,2,4]triazolo[4,3-a]quinoxalin-4-amine:

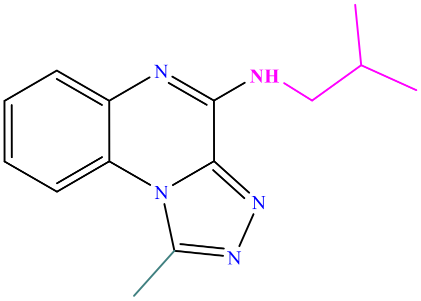

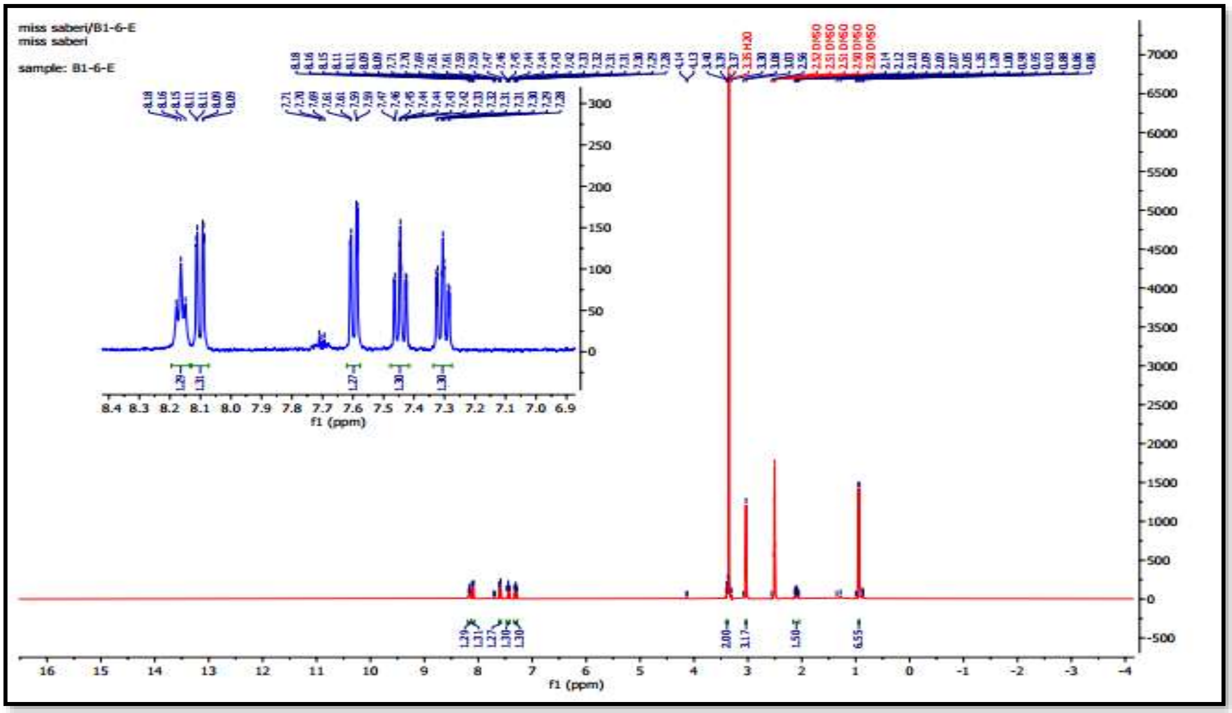

miss saberi/B1-6-E
miss saberi
sample: B1-6-E
f1 (ppm)

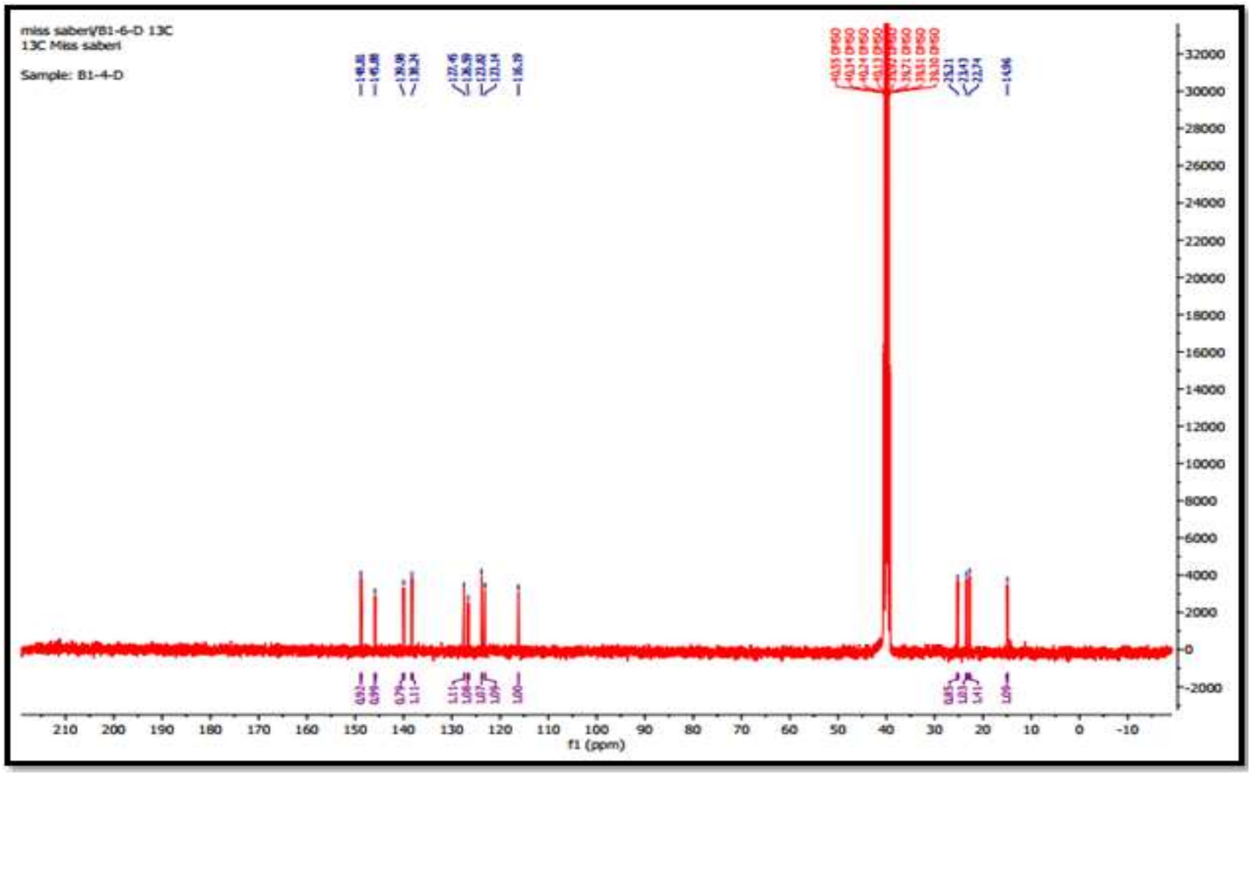

miss saberi/B1-6-D 13C
13C Miss saberi
Sample: B1-4-D
f1 (ppm)

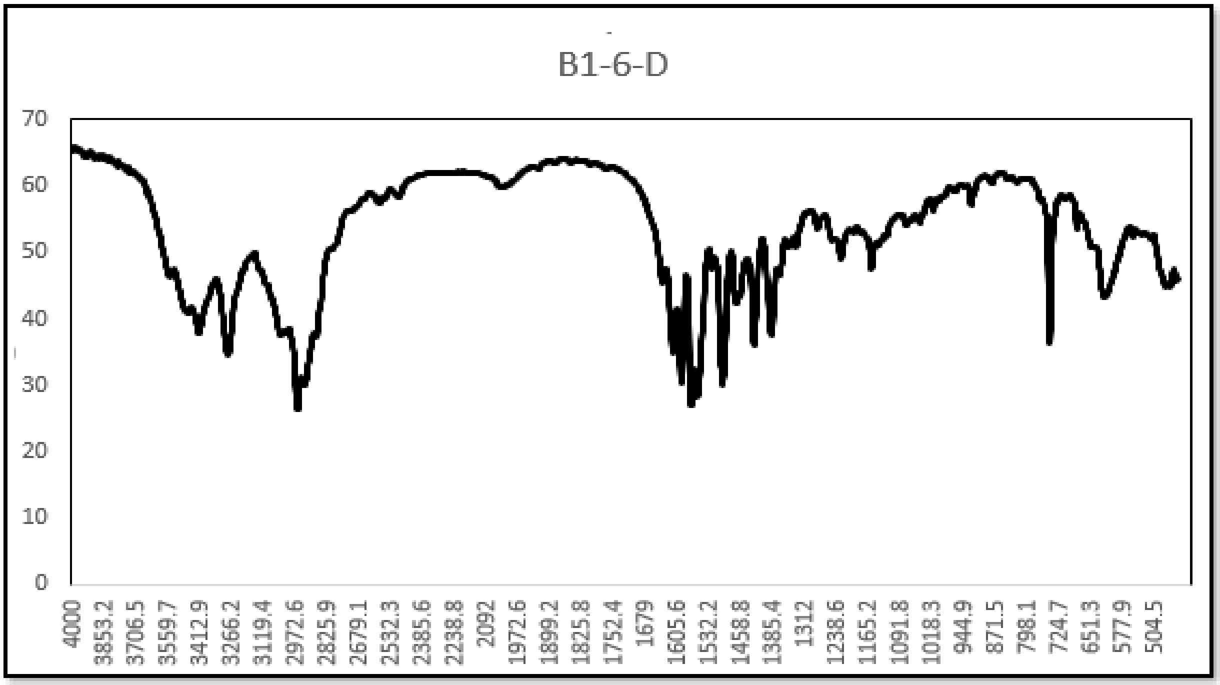
B1-6-D
70
60
50
40
30
20
10
0
4000
3853.2
3706.5
3559.7
3412.9
3266.2
3119.4
2972.6
2825.9
2679.1
2532.3
2385.6
2238.8
2092
1972.6
1899.2
1825.8
1752.4
1679
1605.6
1532.2
1458.8
1385.4
1312
1238.6
1165.2
1091.8
1018.3
944.9
871.5
798.1
724.7
651.3
577.9
504.5

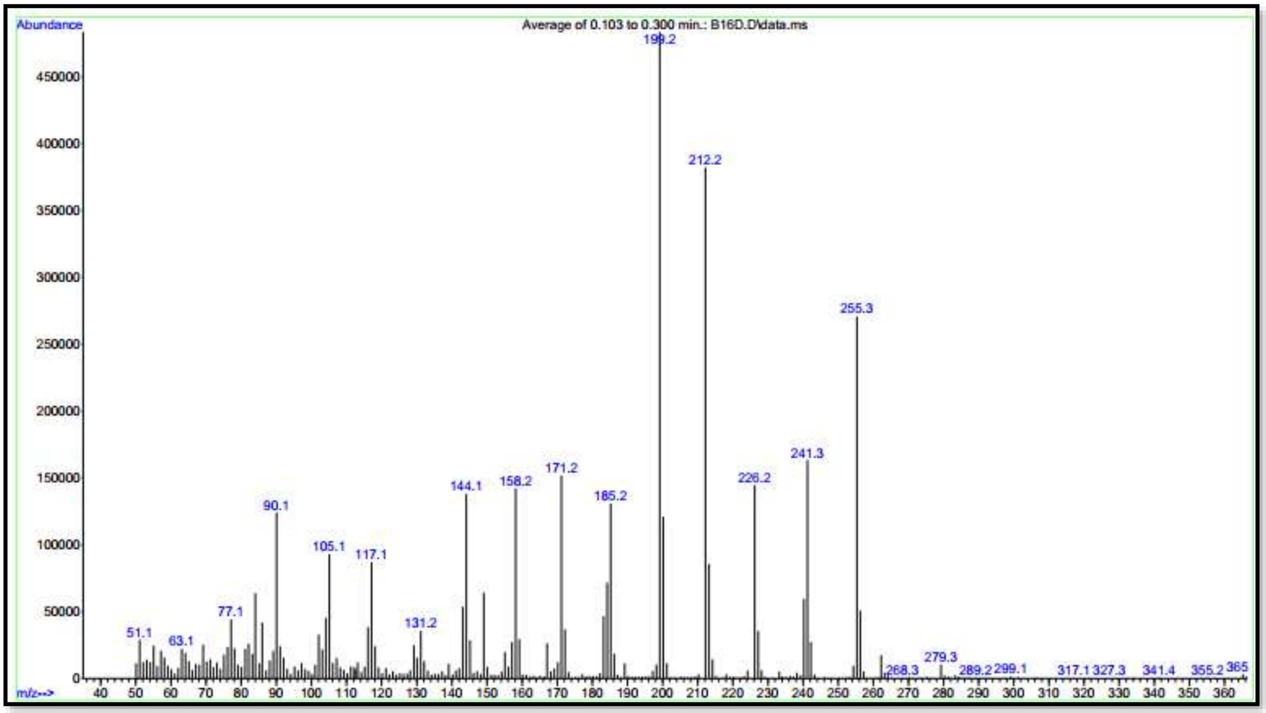
Abundance
Average of 0.103 to 0.300 min.: B16D.D\data.ms
199.2
212.2
255.3
241.3
226.2
171.2
158.2
144.1
185.2
90.1
105.1
117.1
77.1
131.2
51.1
63.1
268.3
279.3
289.2
299.1
317.1
327.3
341.4
355.2
365
m/z-->

Result Table (ESTD - E:\results\140406\140406-Saberi\14040612-Saberi-B16D - INT7 - 1)

	Reten. Time [min]	Response	Weight [mg]	Weight [%]	Peak Type	Element Name	Carbon Response Ratio
1	1.090	2237.339	0.996	27.61	Ordnr	N	???
2	1.853	15613.270	2.334	64.68	Ordnr	C	???
3	10.350	8460.221	0.278	7.71	Ordnr	H	???
	Total		3.608	100.00			

1-Methyl-N-(4-methylpentan-2-yl)-[1,2,4]triazolo[4,3-a]quinoxalin-4-amine:

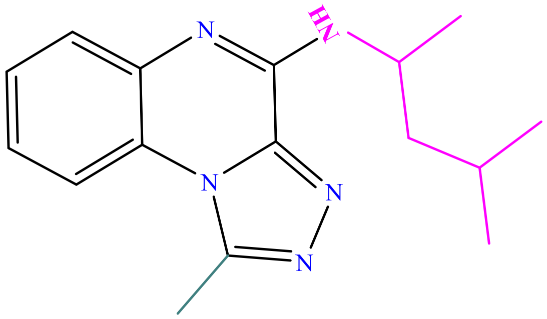

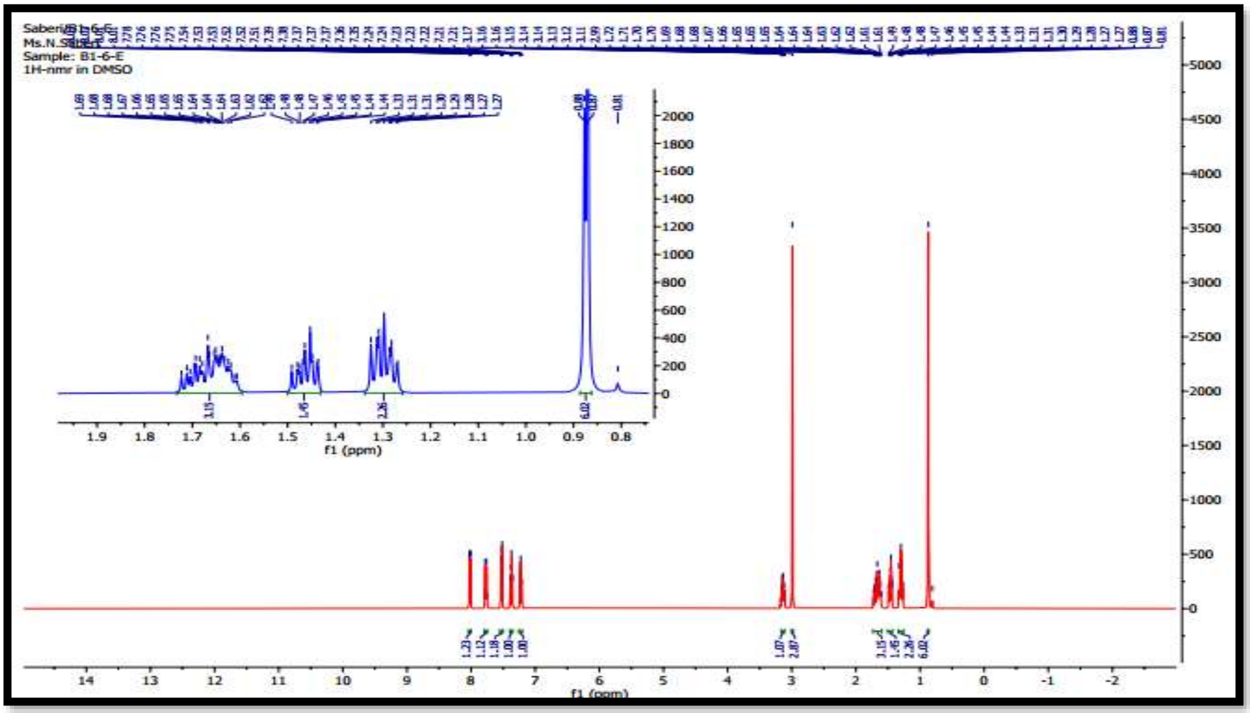

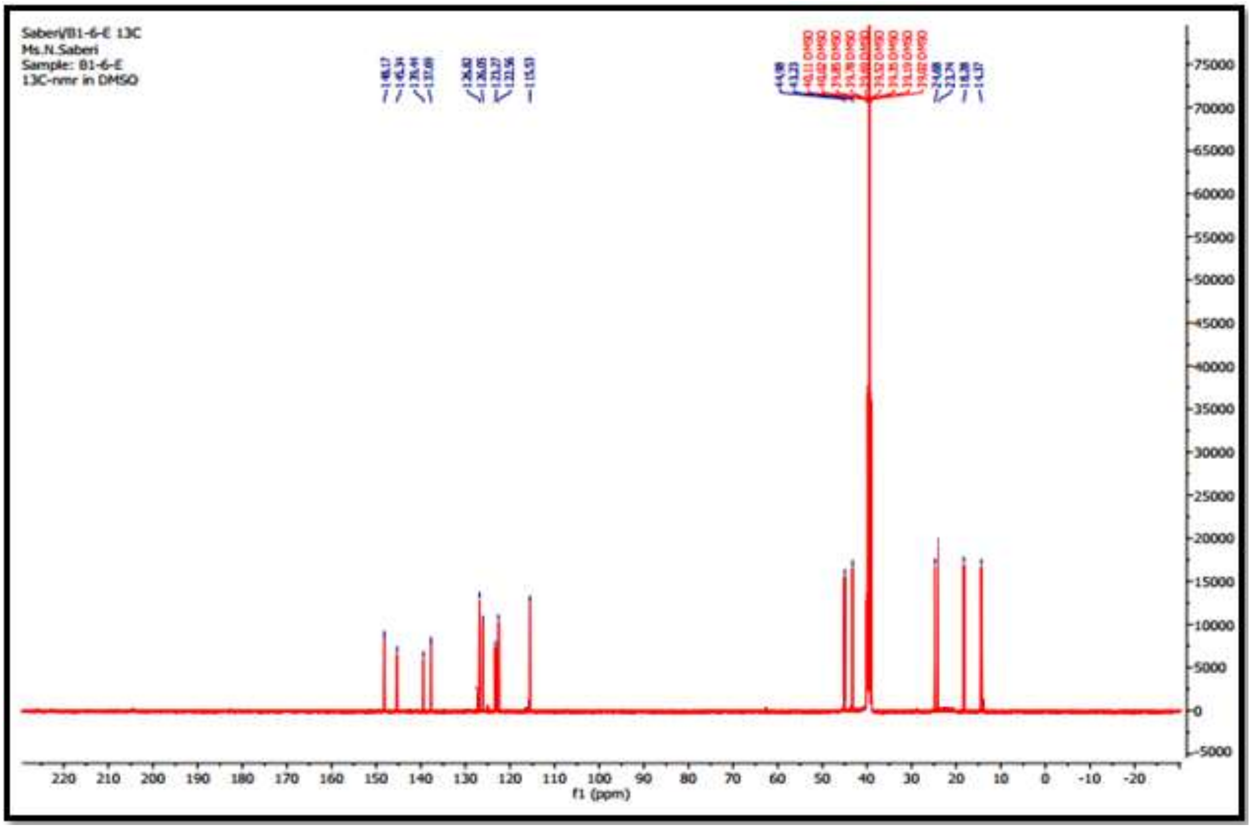

Saberi/B1-6-E 13C
Ms.N.Saberi
Sample: B1-6-E
13C-nmr in DMSO
f1 (ppm)

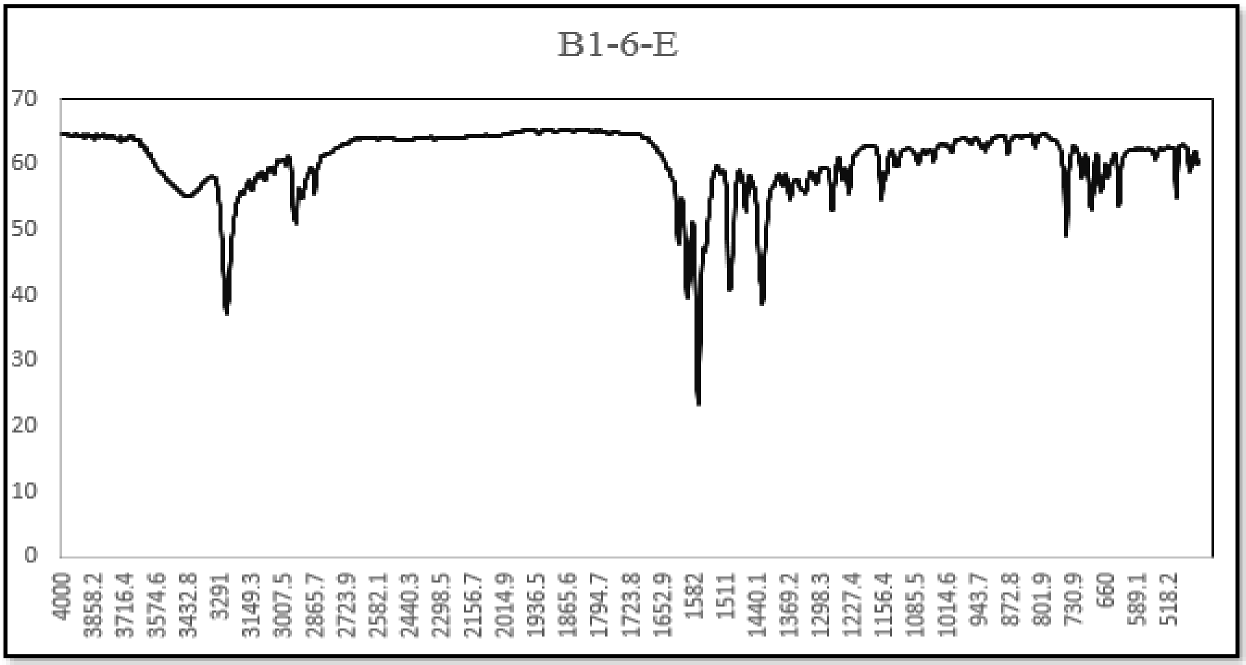

B1-6-E

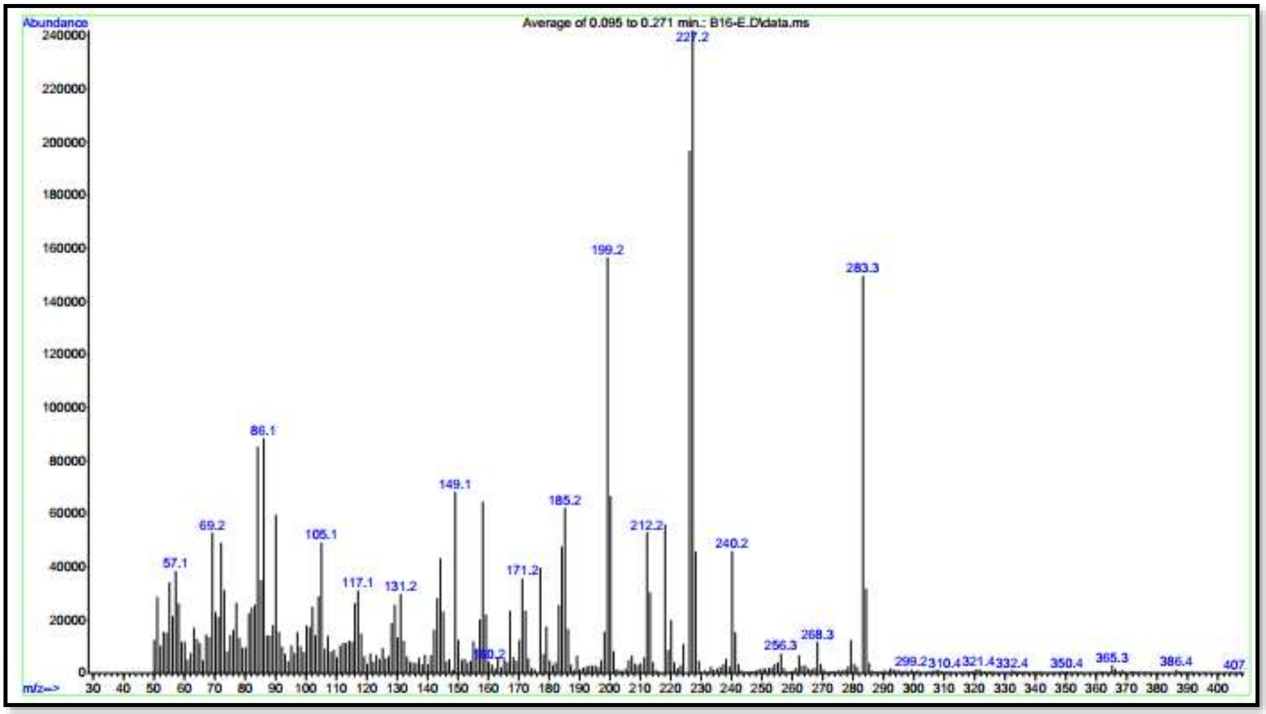

Result Table (ESTD - E:\RESULTS\140406\140406-SABERI\14040605-SABERI-B16E - INT7 - 1)

	Reten. Time [min]	Response	Weight [mg]	Weight [%]	Peak Type	Element Name	Carbon Response Ratio
1	1.087	3038.837	1.165	24.81	Ordnr	N	???
2	1.793	20855.027	3.180	67.72	Ordnr	C	???
3	10.970	8281.157	0.351	7.47	Ordnr	H	???
	Total		4.696	100.00			

N-isopropyl-1-methyl-[1,2,4]triazolo[4,3-a]quinoxalin-4-amine:

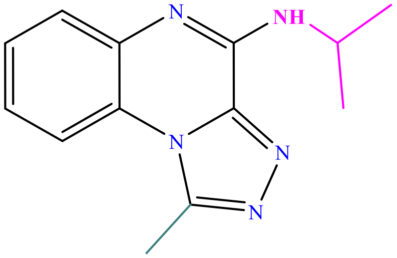

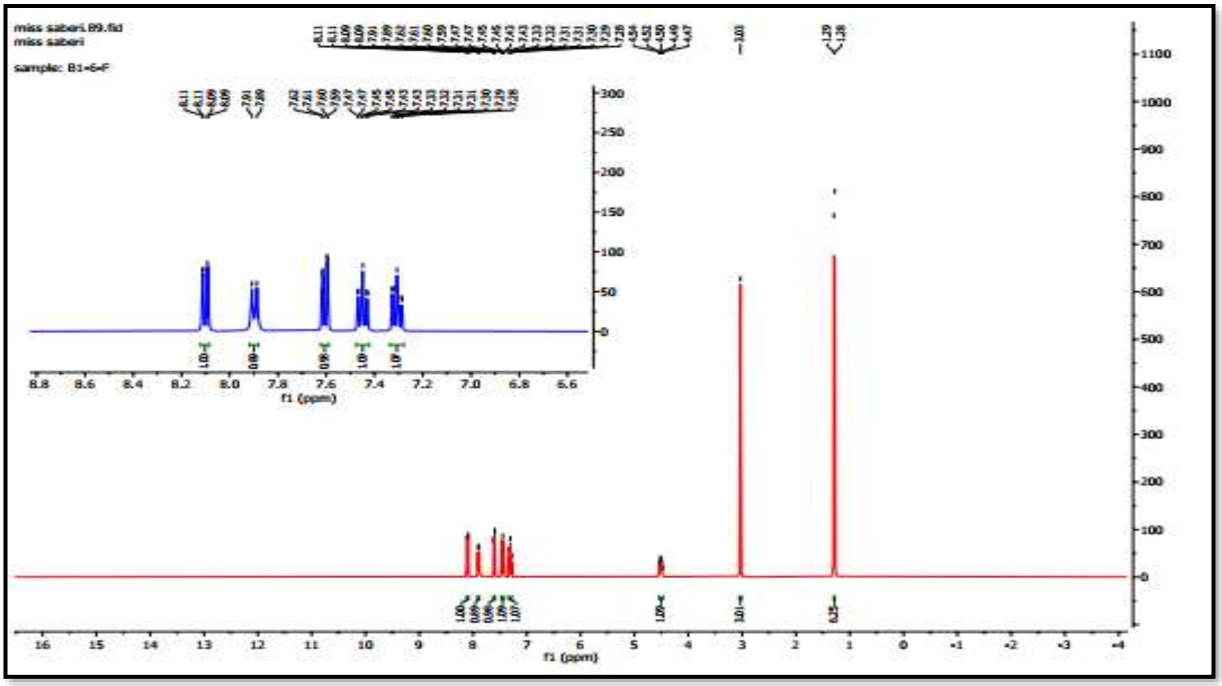
miss saberi.B9.fid
miss saberi
sample: B1-6-F
f1 (ppm)

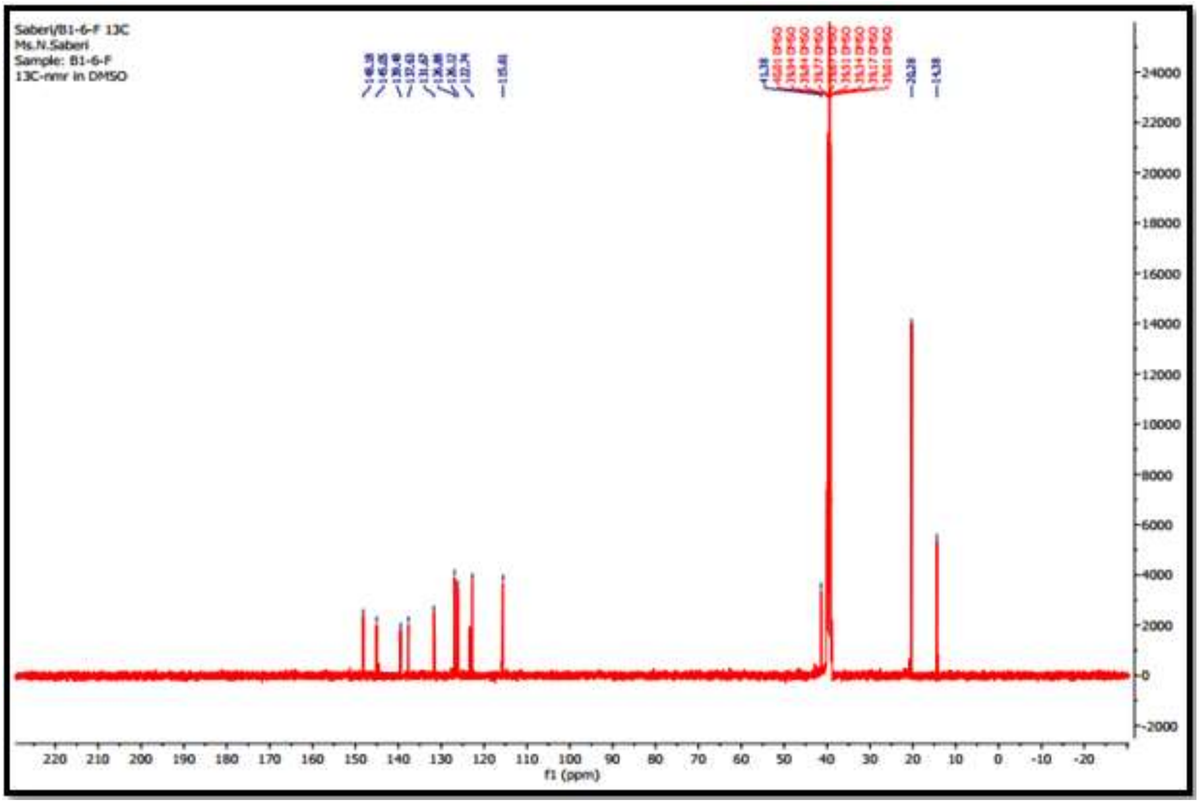
Saberi/B1-6-F 13C
Ms.N.Saberi
Sample: B1-6-F
13C-nmr in DMSO
f1 (ppm)

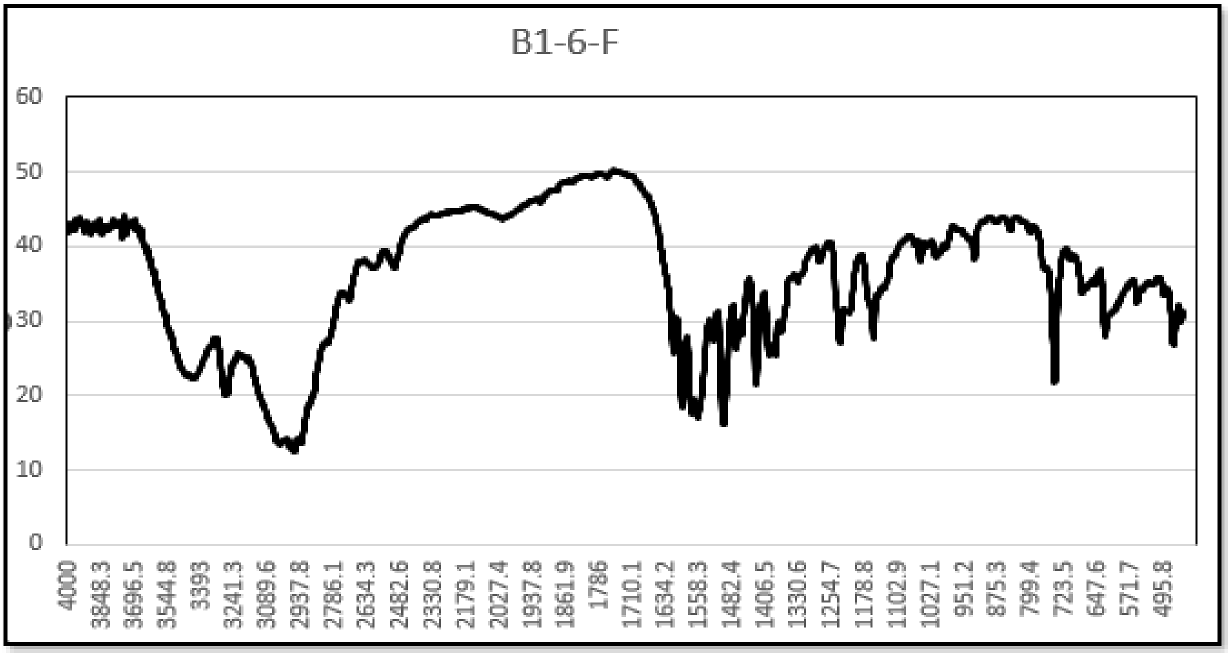

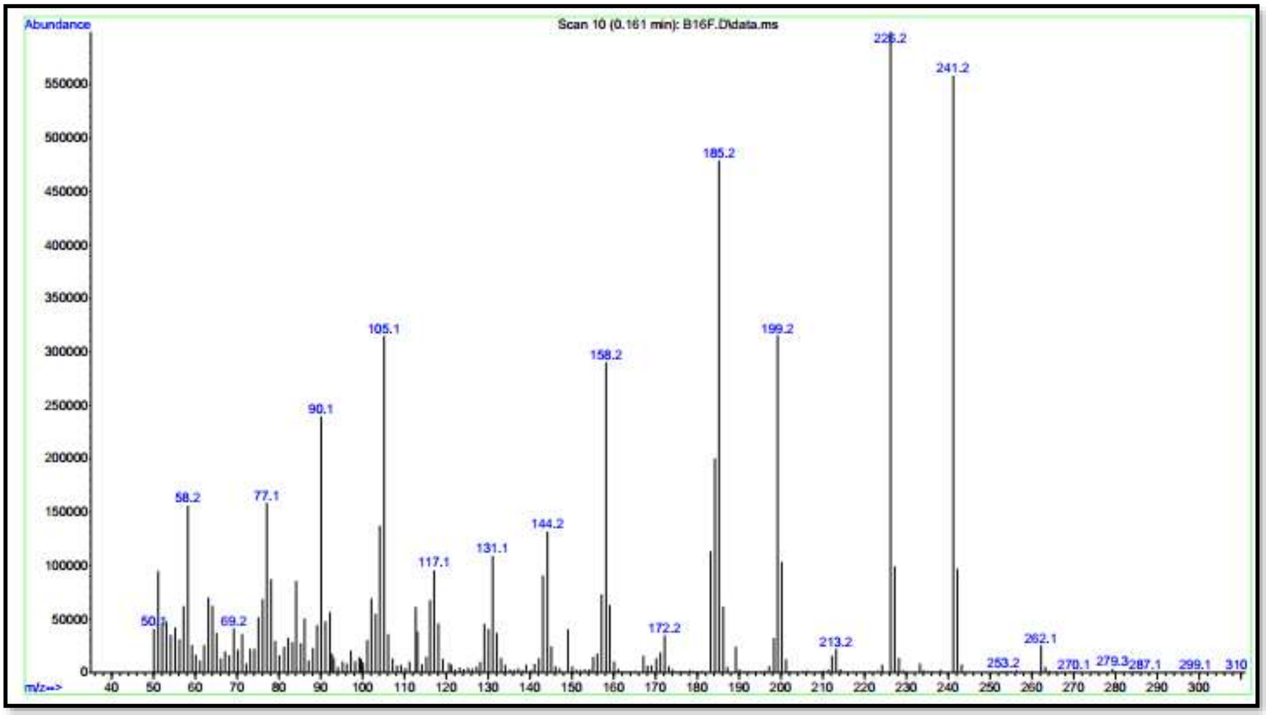

Result Table (ESTD - E:\results\140406\140406-Saberi\14040605-Saberi-B16F - INT7 - 1)

	Reten. Time [min]	Response	Weight [mg]	Weight [%]	Peak Type	Element Name	Carbon Response Ratio
1	1.087	3254.115	1.318	29.02	Ordnr	N	???
2	1.820	20702.690	2.931	64.51	Ordnr	C	???
3	8.497	5706.965	0.294	6.47	Ordnr	H	???
	Total		4.543	100.00			

N-(4-methylpentan-2-yl)-[1,2,4]triazolo[4,3-a]quinoxalin-4-amine:

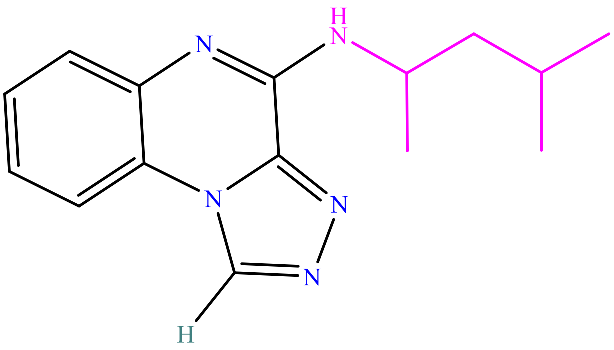

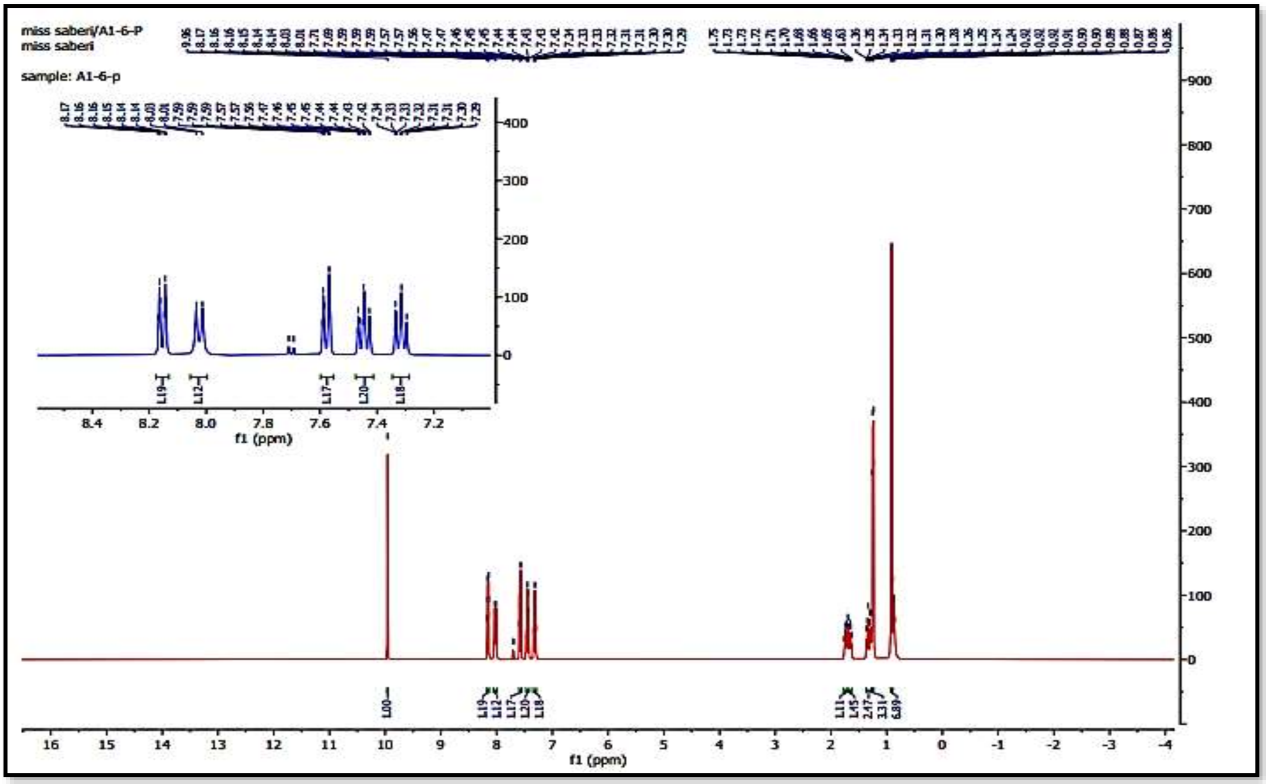

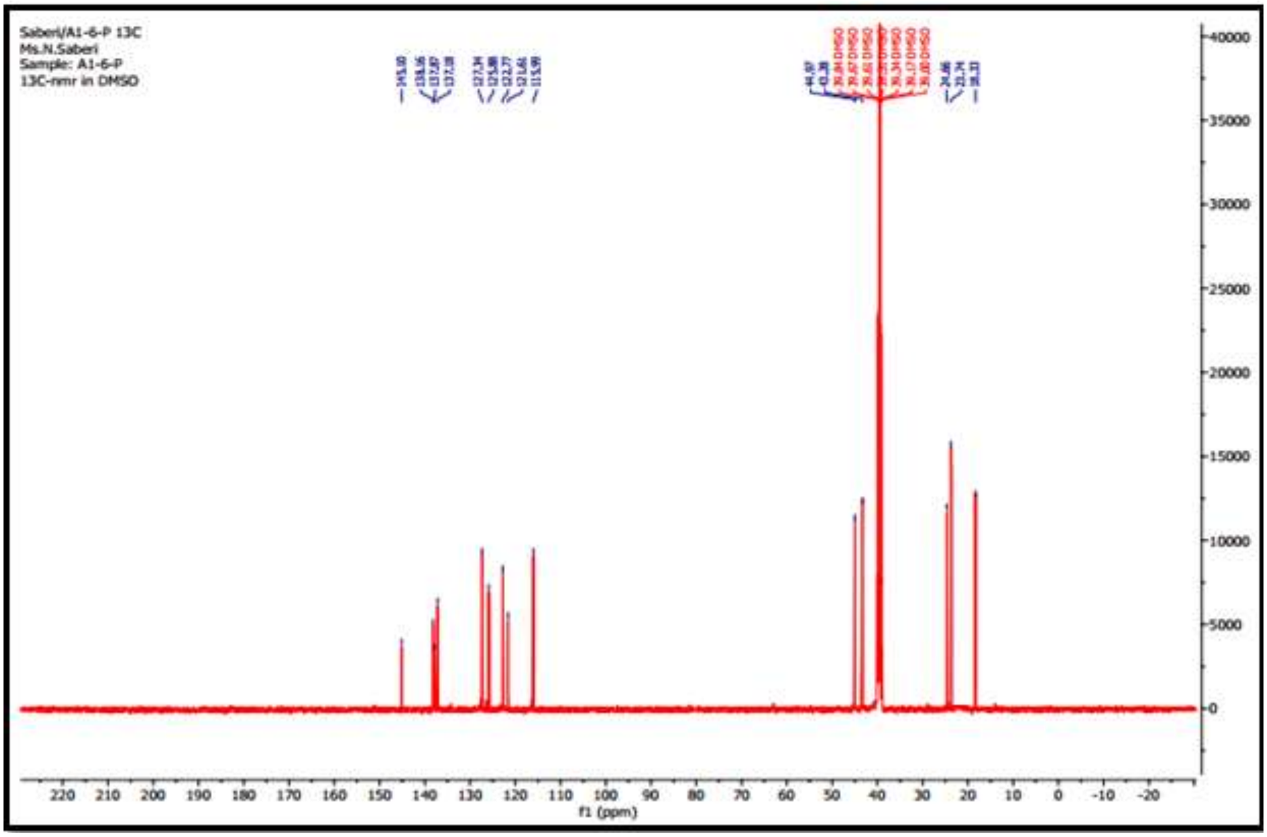
Saberi/A1-6-P 13C
Ms.N.Saberi
Sample: A1-6-P
13C-nmr in DMSO
f1 (ppm)

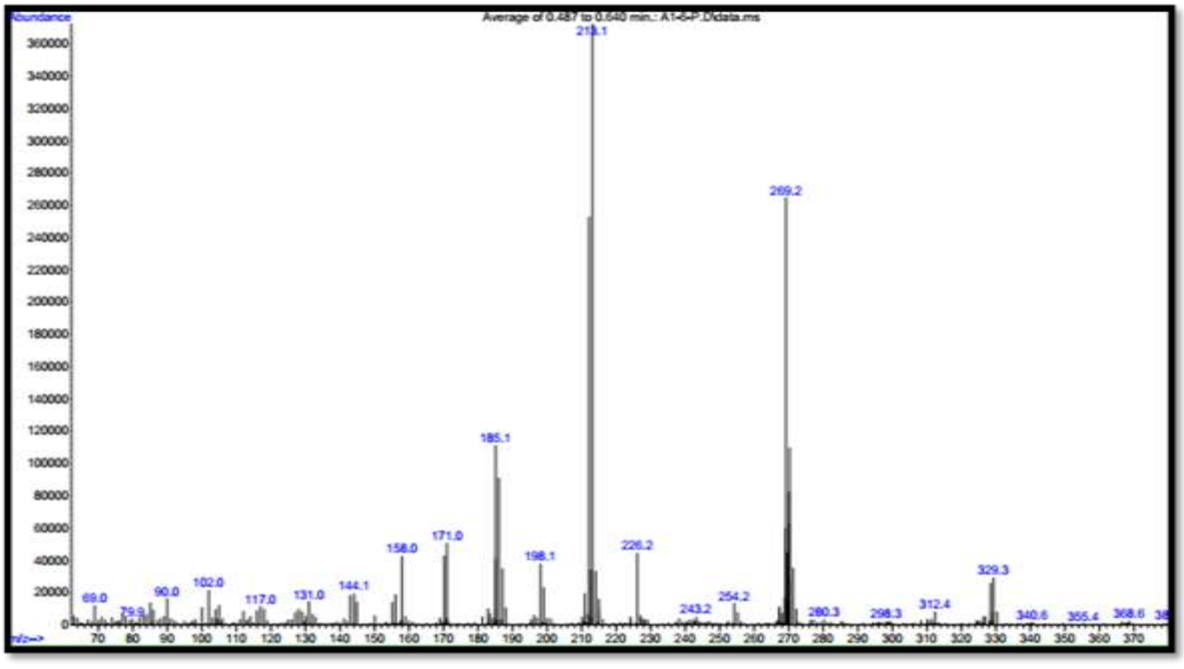
Abundance
Average of 0.487 to 0.640 min.: A1-6-P.D\data.ms
269.2
185.1
158.0
171.0
198.1
226.2
69.0
79.9
90.0
102.0
117.0
131.0
144.1
243.2
254.2
280.3
298.3
312.4
329.3
340.6
355.4
368.6
m/z-->

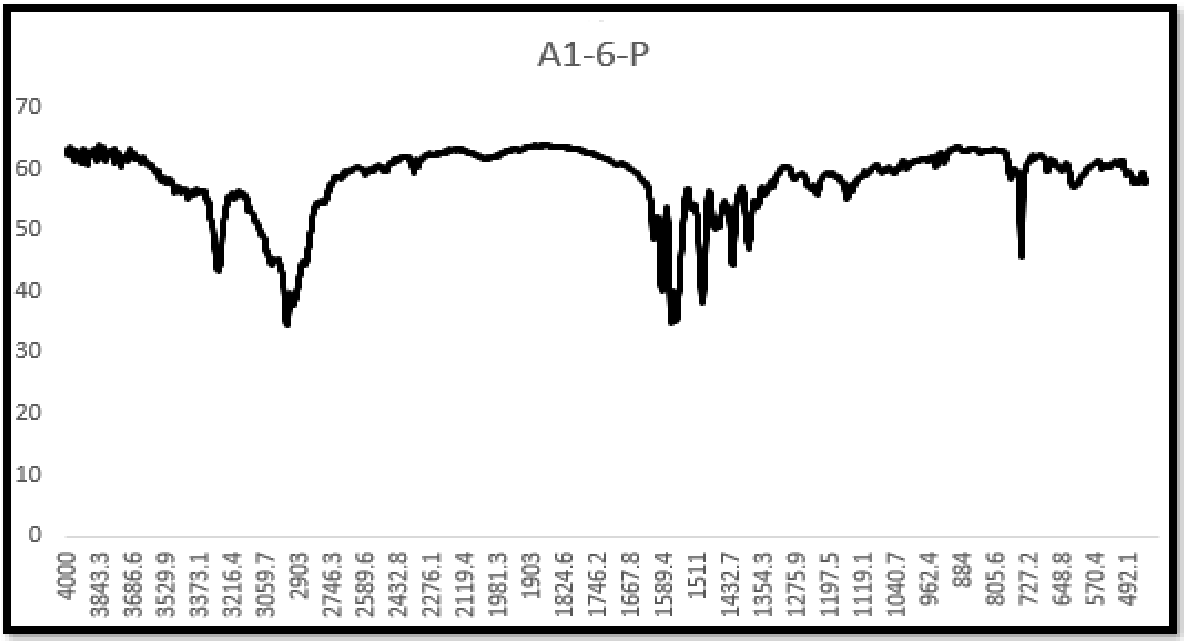

Result Table (ESTD - E:\results\140406\140406-Saberi\14040612-Saberi-A16P - INT7 - 1)

	Reten. Time [min]	Response	Weight [mg]	Weight [%]	Peak Type	Element Name	Carbon Response Ratio
1	1.073	3397.886	1.360	26.11	Ordnr	N	???
2	1.773	22259.955	3.458	66.39	Ordnr	C	???
3	11.190	8669.863	0.391	7.50	Ordnr	H	???
	Total		5.209	100.00			

4-(2-phenylhydrazineyl)-[1,2,4]triazolo[4,3-a]quinoxaline:

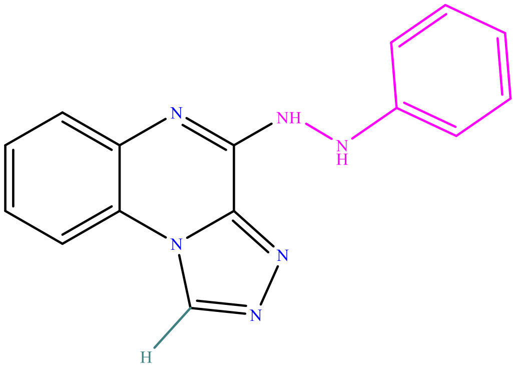

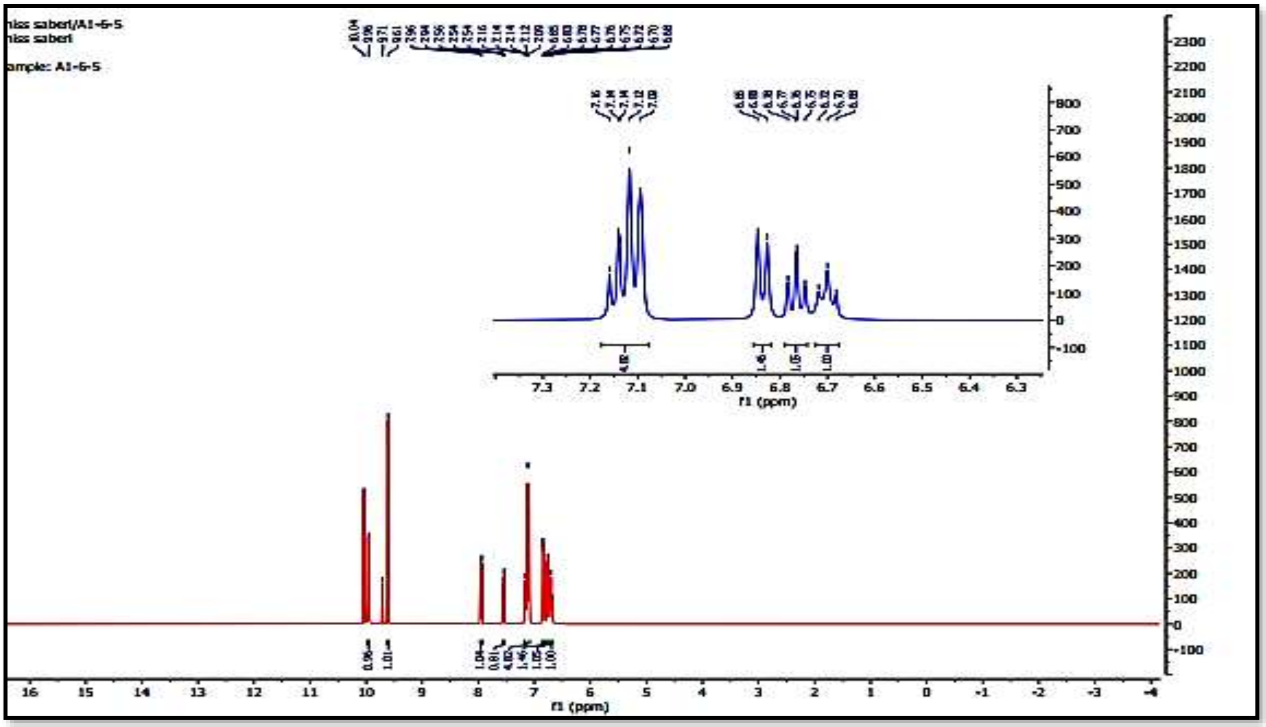

ample: A1-6-S
f1 (ppm)

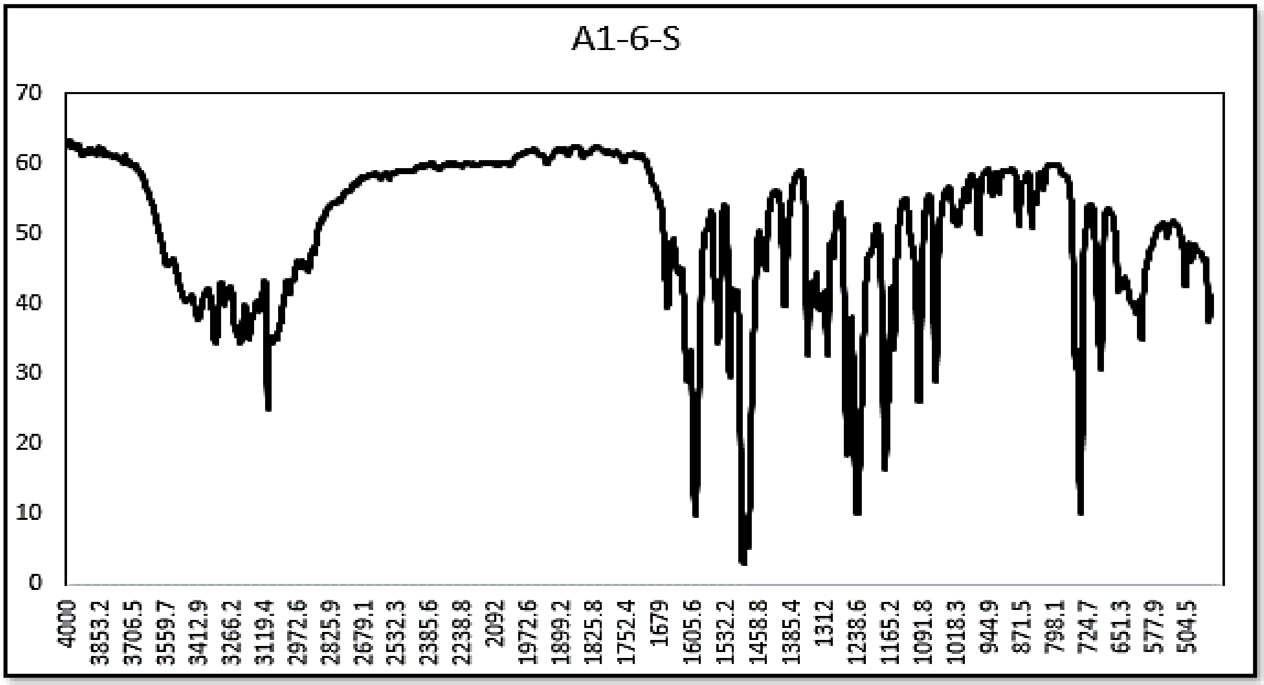

A1-6-S

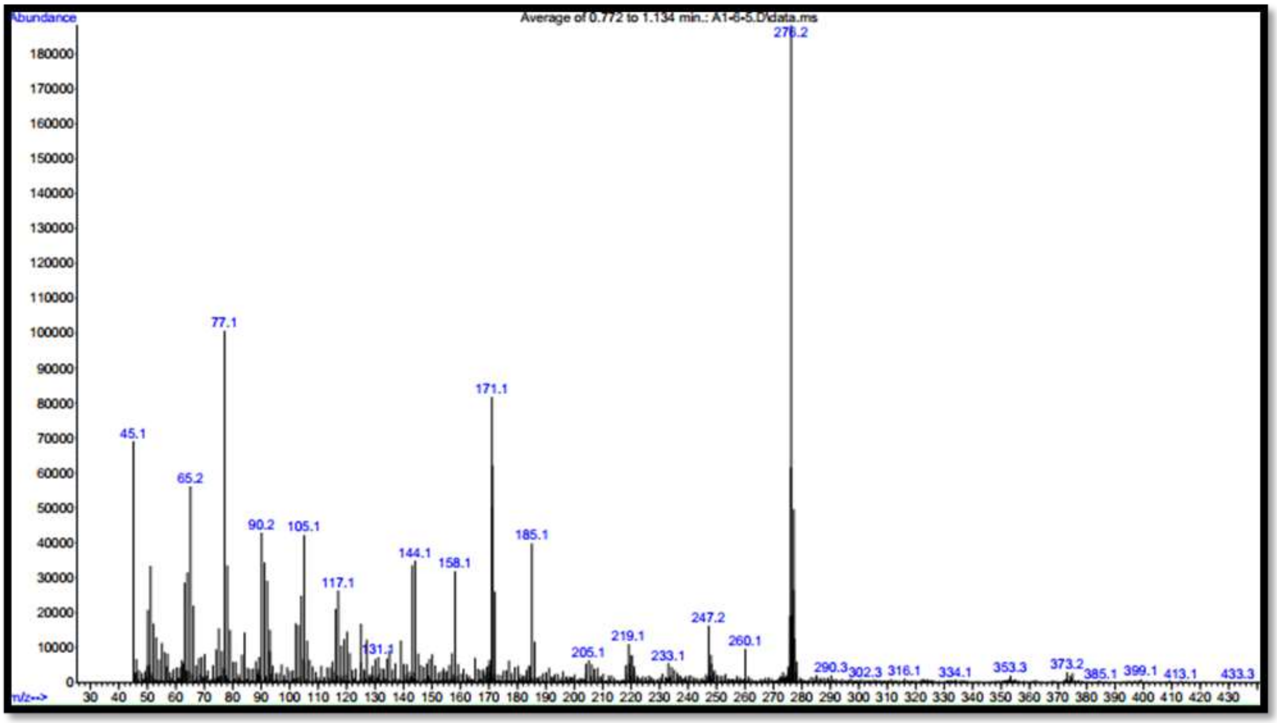

1-methyl-4-(2-phenylhydrazineyl)-[1,2,4]triazolo[4,3-a]quinoxaline:

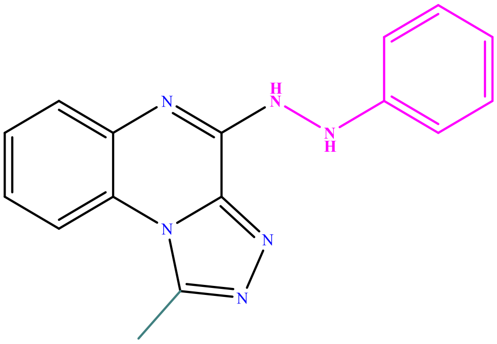

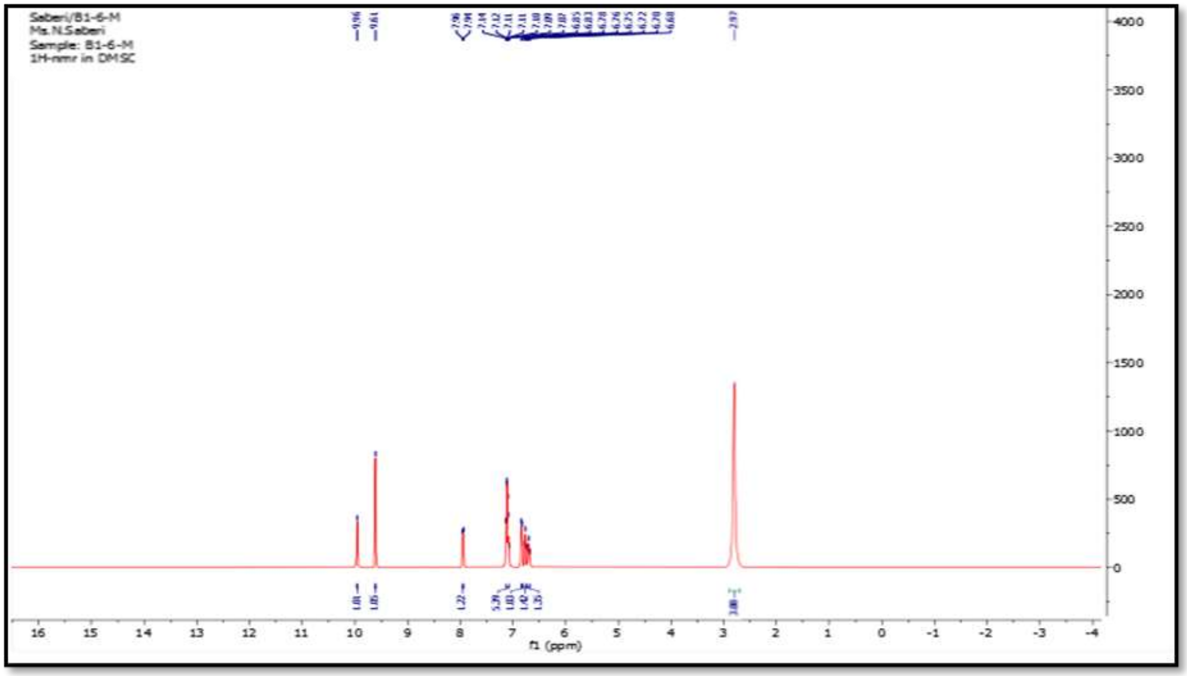
Saberi/B1-6-M
Ms.N.Saberi
Sample: B1-6-M
1H-nmr in DMSC
f1 (ppm)

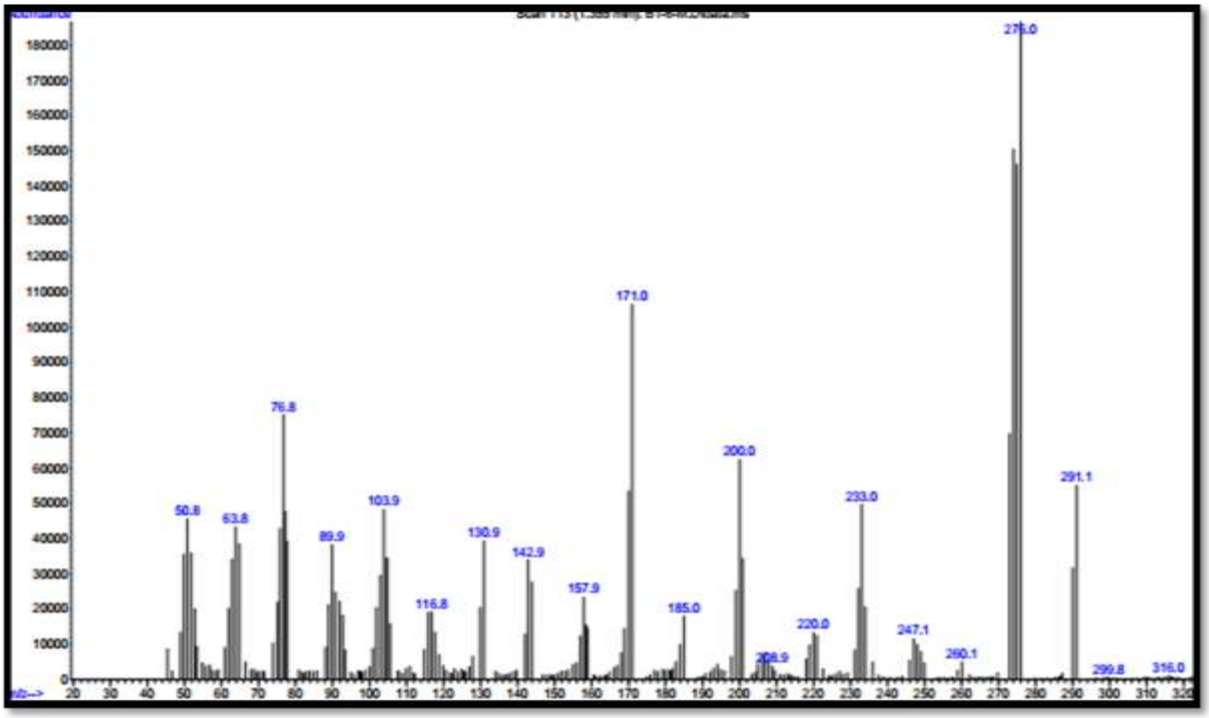
275.0
171.0
76.8
200.0
291.1
233.0
50.8
63.8
103.9
89.9
130.9
142.9
157.9
116.8
185.0
220.0
247.1
208.9
260.1
299.8
316.0

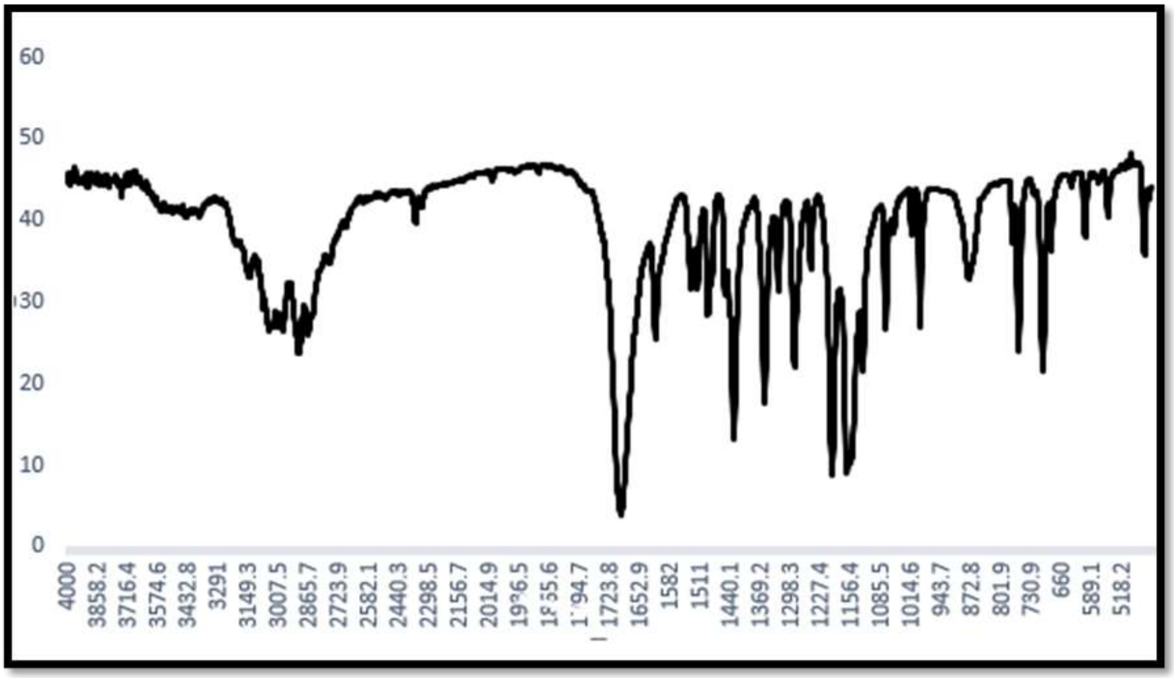

Result Table (ESTD - E:\results\140406\140406-Saberi\14040612-Saberi-B16M - INT7 - 1)

	Reten. Time [min]	Response	Weight [mg]	Weight [%]	Peak Type	Element Name	Carbon Response Ratio
1	1.100	81.114	0.148	29.00	Ordnr	N	???
2	2.097	2208.368	0.339	66.24	Ordnr	C	???
3	9.343	3500.304	0.025	4.76	Ordnr	H	???
	Total		0.512	100.00			
